# Stereochemical Reassignment by Total Synthesis of an Ocular Pyridinium Bisretinoid of Retinal Pigment Epithelium Lipofuscin: *iiso*-A2E Is *i*‑A2E

**DOI:** 10.1021/acs.orglett.5c04694

**Published:** 2025-12-04

**Authors:** Brais Vidal, Oscar Iglesias-Menduiña, Belén Vaz, Rosana Álvarez, Claudio Martínez, Ángel R. de Lera

**Affiliations:** CINBIO, Departamento de Química Orgánica, 16784 Universidade de Vigo, IBIV, Lagoas-Marcosende, 36310 Vigo, Spain

## Abstract

The stereoselective synthesis of recently discovered lipofuscin fluorophore *iiso*-A2E, a C3,C4-bis-polyenylpyridinium salt derived from all-*trans*-retinal, which has been detected in human, pig, mouse, and bovine eyes, has been completed. The Suzuki–Miyaura cross-coupling reaction and Horner–Wadsworth–Emmons condensation were the key synthetic steps for the construction of the tetraenyl and pentaenyl arms, respectively, starting from a properly functionalized pyridine-3,4-dialdehyde surrogate, followed by pyridine alkylation. Subtle changes in the ^1^H NMR and ^13^C NMR spectra of the synthetic compound and natural compound suggested the structural revision of the latter, for which the C13′=C14′ *E* isomer at the longer C3-unsaturated pentaenyl chain of the pyridinium ring was proposed. The synthesis confirmed the alternative stereostructure of the natural pigment, which should be named *i*-A2E, thus correcting the double bond geometry of the natural fluorophore at C13′=C14′.

## Graphical abstract

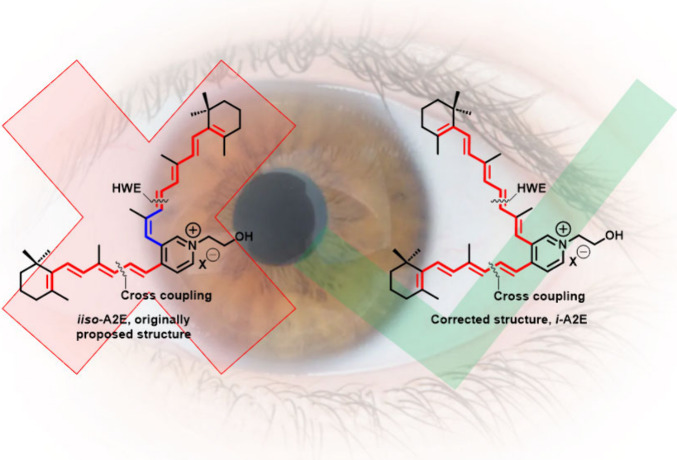

The absorption of light in rod and cone photoreceptors by visual pigments containing a protonated Schiff base derived from the condensation of 11-*cis*-retinal (**1** (Figure 1)) and a lysine group (Lys296) of the protein opsin is the first event in the complex visual process in vertebrates.^1–3^ Light-induced activation of the photoreceptors triggers the isomerization of the chromophore and the release of all-*trans*-retinal (**2**).^4^ The so-called retinoid cycle, the reverse conversion of all-*trans*-retinal (**2**) to regenerate visual chromophore **1**, ensures sustained vision. This process involves the reduction to all-*trans*-retinol (**3**) by retinol dehydrogenase, formation of all-*trans*-retinyl esters (**4** (Figure 1)) by lecithin retinol acyl transferase, conversion to 11-*cis*-retinol (**5**)^2^ promoted by retinal pigment epithelium-specific 65 kDa protein (RPE65) isomerohydrolase,^2,5–7^ and final oxidation by retinol dehydrogenase.^1–3^

**Figure 1:**
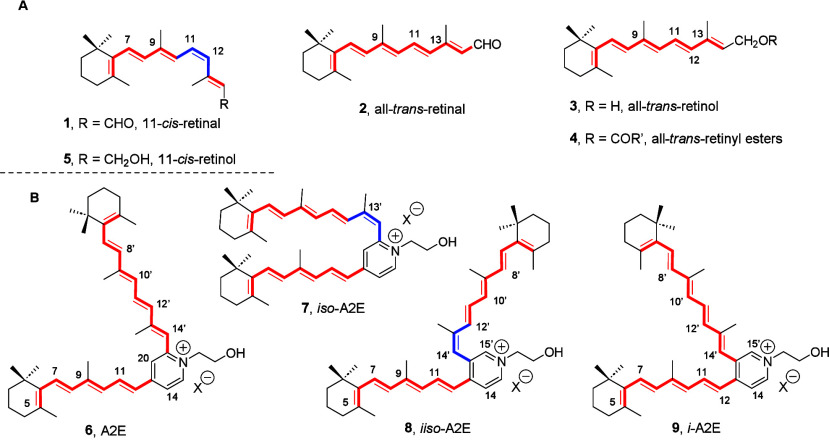
(A) Retinoids were implicated in the visual cycle. (B) Pyridinium bis-retinoids A2E (**6**), *iso*-A2E (**7**), and *iiso-*A2E (**8**)^14^ and structure of geometric isomer *i-*A2E (**9**).

When an excess of all-*trans*-retinal (**2**) accumulates in RPE phagolysosomes^8^ due to anomalies of the retinoid metabolic routes,^9^ lipid metabolism is perturbed, which produces the accumulation of pro-inflammatory lipid-containing granules of fluorescent retinoids, lipids, and protein debris called lipofuscin, one of the causative factors of blindness.^10^ Pyridinium ions substituted with unsaturated fragments (tetraenes and pentaenes (see Figure 1B)) derived from the excess of all-*trans*-retinal (**2**) are the best known of the structurally complex mixture of lipofuscin components.^7^ The most abundant of these fluorophores is *N*-retinylidene-*N*-retinylethanolamine or A2E (**6**),^7,8,11,12^ which stabilizes as a ca. 4:1 photostationary equilibrium mixture with its C13=C14 *cis* isomer, namely *iso*-A2E (**7**).^7,12,13^ Being a *cis* isomer at the long arm of the disubstituted pyridinium ion, *iso*-A2E (**7**) showed an absorbance maximum (λ_max_ = 430 nm)^13^ that is about 10–12 nm blue-shifted relative to that of A2E (λ_max_ = 440 nm).^13^

Among the ocular pigments of lipofuscin different from **6** and **7** that have been more recently isolated from chloroform/MeOH extracts of posterior eyecups harvested from C57BL/6 and BALB/cByJ albine mice,^14^ only *iiso*-A2E (**8** (Figure 1B)) could be fully characterized.^14^ Structural assignment was possible after discovering that pyridinium bisretinoid *iiso*-A2E (**8** (Figure 1B)) could be produced in larger amounts by light-mediated isomerization (for example, using sunlight for 35 min) of *iso*-A2E (**7** (Figure 1)),^14^ or as a minor component of the mixture generated upon treatment of all-*trans*-retinal with ethanolamine.^14^ *iiso*-A2E (**8**, also described as URP2) and so-called unrecognized pigment 1 (URP1) were also detected in human, pig, and bovine RPE, and in the human donor eye.^14^

^1^H and ^13^C NMR spectroscopic data analysis of solutions in CD_3_OD, with the aid of DEPT135, HSQC, and HMBC experiments, established the proposed structure for *iiso*-A2E (**8** (Figure 1B)) with unsaturated chains at vicinal carbons,^14^ and NOESY data further confirmed the unusual connection of the pentaenyl side chain to the pyridinium ion C3 position (C20 skeletal numbering (see refs 15 and 16)) instead of position C2 (C15′ skeletal numbering^15^) as present in A2E (**6**).

Interestingly, whereas the *E* geometry was assigned by ^1^H NMR spectroscopic analysis to most of the di- and trisubstituted double bonds of *iiso*-A2E (**8** (Figure 1B)) using the value of coupling constants and/or NOE correlations, the *Z* geometry attributed to the trisubstituted C13′=C14′ olefin was proposed by apparent NOESY correlations observed between C13′-CH_3_ and C14′-H.^14^

The structural proposal for *iiso*-A2E (**8** (Figure 1B))^14^ as a positional isomer of *iso*-A2E (**7**) caught our attention. As a follow-up to our contributions to the stereocontrolled synthesis of bioactive polyenes,^17–20^ we addressed its total synthesis with the aim to confirm the proposed structure of this structurally unusual pyridinium bisretinoid.^14^

Based on our prior experience on carotenoids,^18^ the classical Horner–Wadsworth–Emmons (HWE) condensation reaction^21–26^ was first envisaged as a reasonable approach to construct the vicinal polyenic substituents of the *iiso*-A2E pyridinium ion (Scheme 1). Sharing a common terminal cyclohexenetrienyl fragment on each branch, bis-polyenic pyridine precursor **10** was first considered to result from the two-directional *E*-selective HWE condensation reaction of the anion derived from conjugated heptatrienylphosphonate **11** ^18,27^ and (*Z*)-3-(2-methyl-3-oxoprop-1-en-1-yl)­isonicotinaldehyde (**12**).

**Scheme 1:**
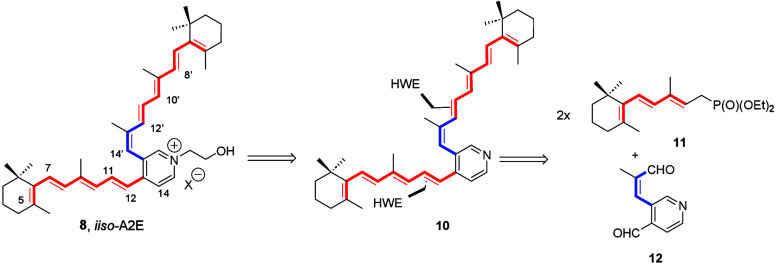
Retrosynthetic Analysis of *iiso*-A2E (**8**)

As a component for the synthesis of dialdehyde **12**, (*Z*)-alkenylstannane **15** ^28,29^ was obtained from propargylic alcohol **13** as previously described^28^ in a 39% combined yield. Stereoselective addition of lithium dimethylcuprate to **13**, trapping with I_2_,^30–32^ and protection afforded alkenyl iodide **14**,^28,33^ which was treated with *t*-BuLi^34^ and then with *n*-Bu_3_SnCl at −78 °C to provide **15**. Stille–Migita–Kosugi cross-coupling reaction^35–38^ with commercial 3-bromoisonicotinaldehyde **16** promoted by Pd­(PPh_3_)_2_Cl_2_ and PPh_3_ in toluene^15^ at 110 °C (Scheme 2) afforded (*Z*)-2-alkenyl-substituted isonicotinaldehyde **17** in 90% yield. Unfortunately, attempts to deprotect **17** with TBAF or acidic media prior to allylic oxidation on route to dialdehyde **12** led to undesired secondary products, and therefore, we selected instead the stepwise construction of the polyene fragments at positions C4 and C3 of the pyridine skeleton present in **8**.

**Scheme 2:**
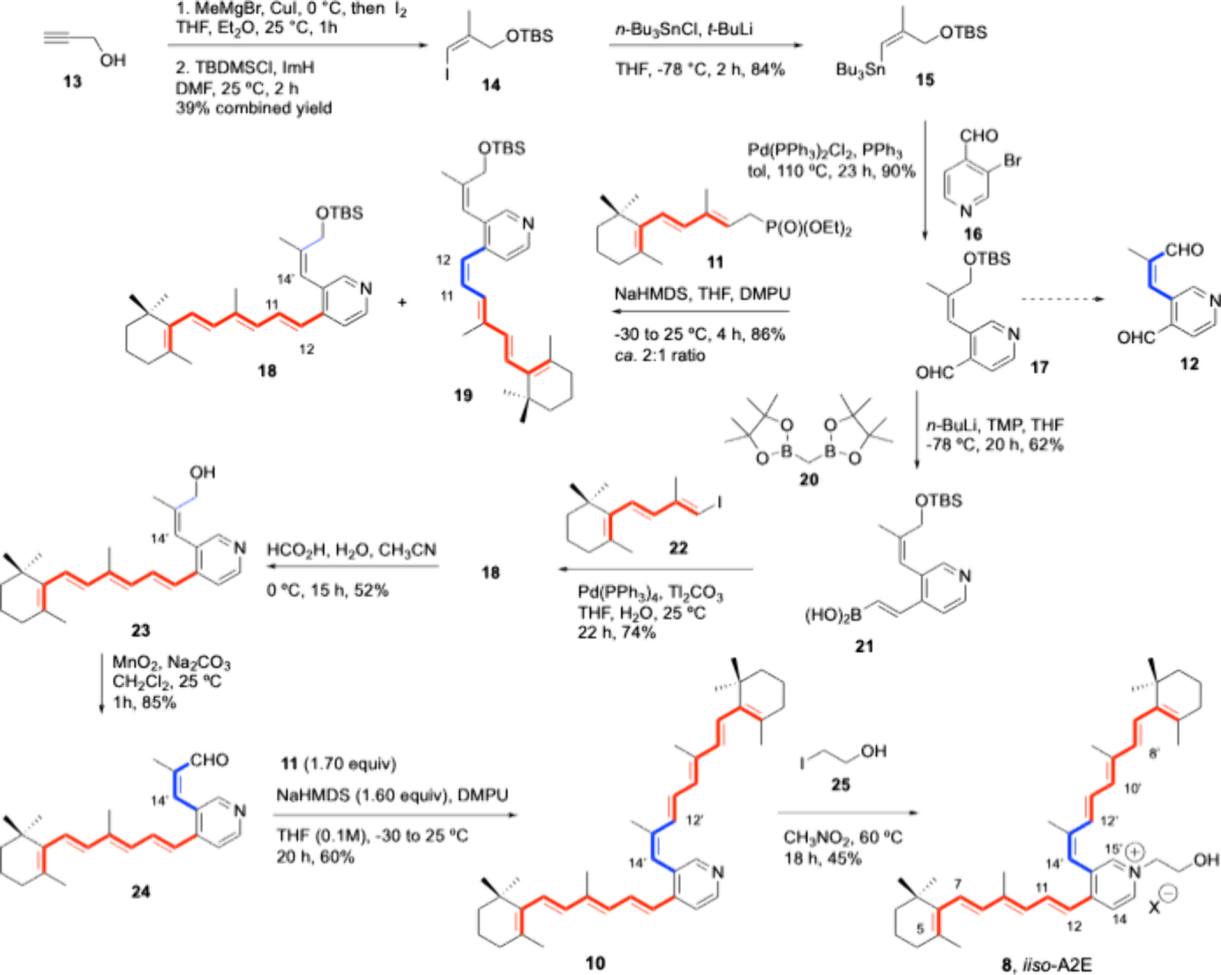
Stepwise Suzuki–Miyaura Cross-Coupling Reaction and HWE Condensation for the Total Synthesis of Proposed *iiso*-A2E (**8**)

The best reaction conditions for the HWE condensation reaction^21–26^ of **17** with heptatrienylphosphonate **11** ^18,27^ used an excess (1.7 molar equivalents) of the anion generated with NaHMDS (1.5 molar equivalents) in THF and DMPU and afforded the C4-tetraenyl-substituted pyridine as a ca. 2:1 mixture of *E* and *Z* isomers (**18** and **19**, respectively) in 86% yield (Scheme 2).^39^

As an alternative to overcome the low stereoselectivity of the HWE condensation, we turned our attention to the construction of the C10–C11 bond using Pd-catalyzed Suzuki–Miyaura cross-coupling reactions.^40–42^ Alkenyl extension at position C4 of the pyridine ring with functionalization as alkenylboronate was carried upon treatment of **17** with bis­[(pinacolato)­boryl]­methane **20** and lithium 2,2,6,6-tetramethylpiperidine using the boron-Wittig olefination variant.^43^ The reaction afforded alkenylboronic acid **21** in 62% yield and minor amounts of the expected alkenylpinacolboronate, as confirmed by NMR characterization after flash-column chromatography. Upon stirring solutions of **21** and trienyl iodide **22** ^44^ in THF/H_2_O mixtures with catalytic amounts of Pd­(PPh_3_)_4_ and promoted by Tl_2_CO_3_, targeted C3-alkenyl,C4-tetraenylpyridine **18** was formed in 74% yield. Deprotection of **18** (HCO_2_H, H_2_O, 0 °C, 15 h, 52%) was followed by allylic alcohol oxidation of **23** using MnO_2_ with Na_2_CO_3_ to afford *Z*-enal **24** in 85% yield.

The second HWE reaction^21–26^ of **24** with excess **11** under the same conditions described above was highly stereoselective for the construction of the pentaene branch and afforded **10** in 60% yield. Careful pyridine alkylation of **10** with 2-iodoethanol (**25**) in nitromethane upon heating to 60 °C for 18 h (to prevent the isomerization occurring when using refluxing conditions at 100 °C as reported by Nakanishi for the synthesis of A2E (**6**)^15^) afforded *iiso*-A2E (**8**) in 45% yield.

Comparison of the ^1^H NMR spectroscopic data for synthetic and reported *iiso*-A2E (**8**)^14^ revealed subtle differences, most noticeably at the pyridinium ion region (H15′, H14′, and vicinal hydrogens) and at the methyl groups attached to C­(sp)^2^ (Figure S1 and Tables S1 and S2). Moreover, the UV absorption maxima of synthetic *iiso*-A2E (**8**) (λ_max_ ≈ 340 and 430 nm) did not match the reported values for the natural product (λ_max_ ≈ 352 and 430 nm).^14^ Whereas a hypsochromic shift of about 12 nm (λ_max_ ≈ 430 nm) in the UV spectra of *iiso*-A2E (**8**)^14^ with respect to A2E (**6**; λ_max_ ≈ 442 nm)^15^ was suggestive of a double bond isomer on the long pentaenyl chain at C13′ (13*′Z* or *iso*-A2E (**7**)^14,15^ ), the bathochromic shift of about 16 nm reported^14^ for the shorter tetraenyl chain of *iiso*-A2E (**8**) (λ_max_ ≈ 352 nm; cf. λ_max_ ≈ 336 nm for A2E **6** ^15^) was surprising, unless the relative location of the polyenyl chains altered the absorption wavelength of the vicinal branches on the UV spectra of pyridinium ion positional isomers.

In view of the lack of correspondence of ^1^H NMR, ^13^C NMR, and UV data (Figure S1, Tables S1 and S2, and Figure S2) of synthetic and natural *iiso*-A2E (**8**), we considered instead to synthesize the C13′=C14′ *E* isomer. The proposal took into consideration that the assignment of the double bond geometry of **8** was based on NOE correlations of the C13′-Me substituent (δ ≈ 2.01 ppm) and H14′ substituent (δ ≈ 6.55 ppm) in the ^1^H NMR spectra. However, the chemical shift values of the signal assigned to C13′ (δ ≈ 12.80 ppm) on the ^13^C NMR spectra^14^ seem unlikely to correspond to the *Z* geometry at the trisubstituted olefin, which usually shows chemical shift values at around δ ≈ 20 ppm,^45^ and therefore, alternative methyl substituents were likely irradiated in the NOE correlations of the natural sample.

The approach to (*E*)-2-alkenylisonicotinaldehyde **29** differed from the above in the use of the Suzuki–Miyaura cross-coupling reaction^40–42^ of **16** with (*E*)-alkenylboronate **28**, which was promoted by Pd­(OAc)_2_, PPh_3_, and Na_2_CO_3_ in dioxane at 95 °C (Scheme 3) and afforded **29** in 66% yield. Alkenylboronate **28** was previously formed in 78% yield upon heating to 80 °C solutions of protected (*E*)-3-iodo-2-methylpropenol **26** ^46^ in DMSO with bis­(pinacolato)­diboron **27** under catalysis of Pd­(dppf)­Cl_2_·CH_2_Cl_2_ in the presence of KOAc.^47^

**Scheme 3:**
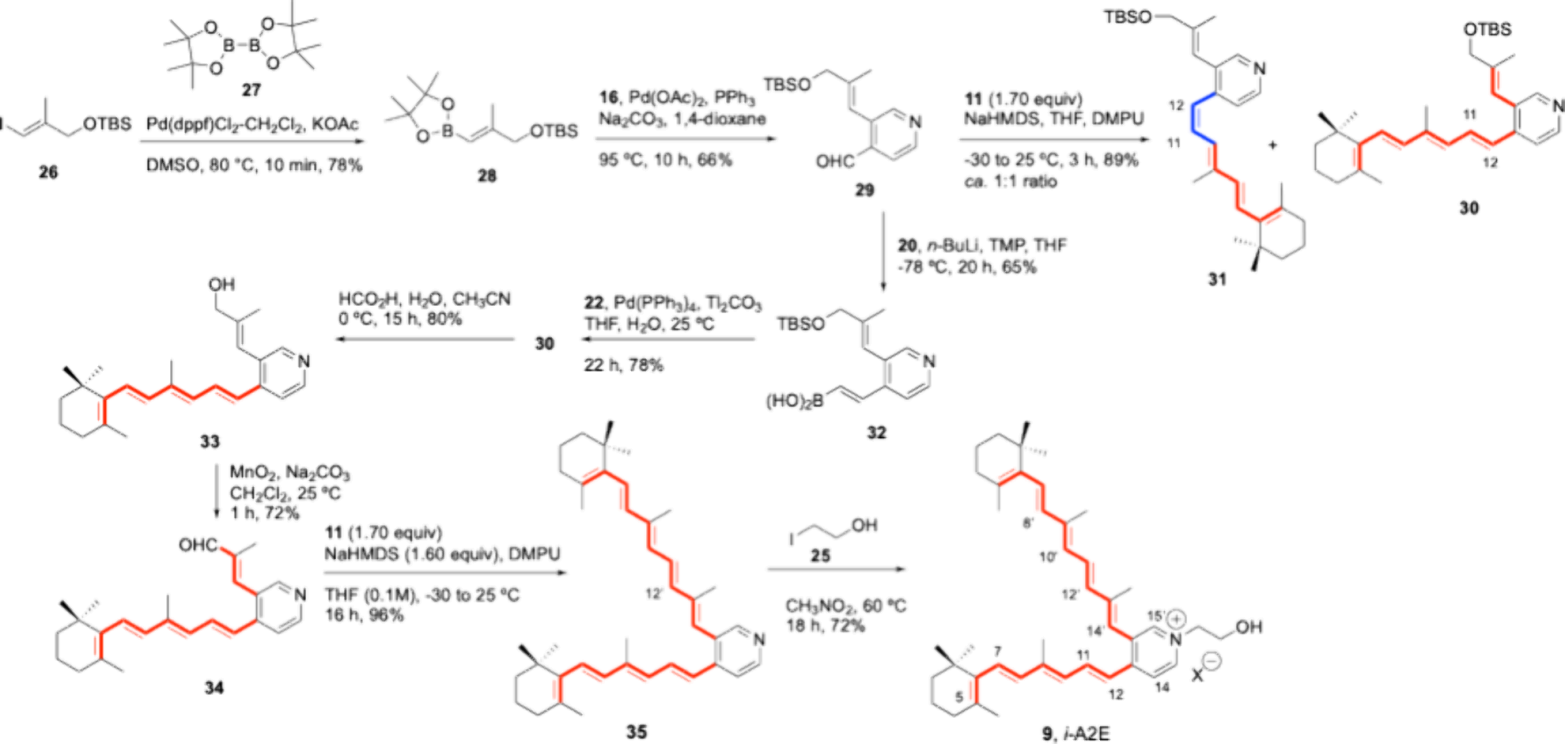
Stepwise Suzuki–Miyaura and HWE Reactions for the Synthesis of *i*-A2E (**9**)

The classical HWE condensation of stereoisomer **29** with the conjugated anion of trienylphosphonate **11** ^18,27,48,49^ took place in 89% yield but lacked stereoselectivity in this case, and a ca. 1:1 mixture of *E*/*Z* double bond isomers at the C11=C12 bond (**30** and **31**, respectively) was isolated. As described for the *Z* isomer in Scheme 2, the alkenyl–alkenyl Suzuki–Miyaura cross-coupling reaction^40–42^ of trienyliodide **22** ^44^ with alkenylboronic acid **32**, which was generated from **29** in 65% yield, led stereoselectively to **30** using catalysis of Pd­(PPh_3_)_4_ and Tl_2_CO_3_ at ambient temperature (Scheme 3) in 78% yield.

The construction of the longer pentaenyl branch of the target followed the same steps (deprotection, oxidation, and stereoselective HWE condensation) described for the *Z* isomer, using the reagents and reaction conditions described above, and afforded bis-polyenylpyridine **35** in higher yields. Alkylation of **35** with 2-iodoethanol **25** in nitromethane at 60 °C for 12 h generated targeted pyridinium salt **9** in 72% yield (Scheme 3).

Excellent superimposition was noted upon comparison of the ^1^H NMR (Figure S1 and Tables S1 and S2) and UV (Figure S2) spectroscopic data for synthetic pyridinium bis-retinoid **9** and those reported for *iiso*-A2E (**8**),^14^ which confirmed that the natural material is indeed the C13′=C14′ isomer of the proposed structure, namely *i*-A2E. The UV data for *i*-A2E (**9**) (λ_max_ ≈ 348 and 430 nm) further confirmed that the longer unsaturated arm exhibits a hypsochromic shift relative to A2E (**6**) (λ_max_ ≈ 440 nm),^15^ with absorption maxima similar to those of *Z* isomer *iso*-A2E (**7**)^15^ (λ_max_ ≈ 430 nm),^14^ which might have led to the incorrect assignment of its geometry. The results are an indication that these general principles^50^ of UV maxima values cannot be extended to the unsaturated branches of positional isomers on the pyridinium ion.

In retrospect, our synthetic work faced problems of purification and characterization when trying to identify *iiso*-A2E (**8**) by ^1^H NMR spectroscopy since these samples proved to be unstable in solution. Comparison of the ^1^H NMR spectra of both synthetic geometric isomers (Figure S1) confirmed that *iiso*-A2E (**8**) also undergoes isomerization to the most stable *i*-A2E (**9**), leading to an equilibrium mixture of ca. 4:1, like that of positional isomers A2E (**6**) and *iso*-A2E (**7**) (Figure 1).

The structural correction would now allow confirmation that *i*-A2E (**9**) derived from retinal (**6**) is the pigment detected in human RPE, where its intracellular accumulation causes *in vitro* membrane damage and loss of cell viability.^14^ *i*-A2E (**9**) is most likely generated *in vivo* from precursor *i*-A2PE and released, as occurs with A2PE, after enzymatic hydrolysis.^14,51^ Moreover, *i*-A2E (**9**) is indeed the pigment generated upon sunlight irradiation of *iso*-A2E (**7**) for 35 min, but not of A2E (**6**).^14^ The positional isomerization reaction was irreversible, since irradiation of *i*-A2E (**9**) did not form *iso*-A2E (**7**), but instead other isomeric products.^14^ Identification and structural determination of these additional eye pigments by chemical synthesis and confirmation of the proposed biogenetic route to *i*-A2E (**9**), wihch includes an unprecedented rearrangement of the unsaturated chain to the vicinal position of the pyridinium ion,^14^ are ongoing.

Age-related macular degeneration (AMD), the most common cause of acquired vision loss in the elderly, is characterized by the overload, in the RPE, of lipofuscin, a mixture of fluorescent retinoids, lipids, and protein debris, which causes photoreceptor cell loss in aging human eyes. During life, human adult RPE cells of the eyes accumulate fluorescent pyridinium bisretinoids as components of lipofuscin. The synthesis of the proposed structure of *iiso*-A2E (**8**), a pyridinium bisretinoid isolated from animal and human eyes and considered to be partially responsible for AMD, and that of its C13′=C14′ *E* isomer, namely *i*-A2E (**9**), has been completed starting from double bond isomers of a bis-functionalized pyridine precursor. The stepwise Suzuki–Miyaura cross-coupling of alkenylboronates **21** and **32** with trienyliodide **11** to afford tetraenes **18** and **31**, respectively, was followed by the HWE olefination of tetraenylisonicotinaldehyde derivatives **30** and **34** with the anion of phosphonate **11** as key steps in the sequence. Comparison of the spectroscopic data of synthetic **8** and **9** with those of the natural product confirmed the identity of the latter and corrected the proposed structure, paving the way to shed additional information about the putative role of these bisretinoids in ocular diseases.^52^

## Supplementary Material



## Data Availability

The data underlying this study are available in the published article and its Supporting Information.

## Notes

Experimental procedures, characterization data, and copies of NMR spectra (PDF)

## References

[1] Zhang J., Choi E. H., Tworak A., Salom D., Leinonen H., Sander C. L., Hoang T. V., Handa J. T., Blackshaw S., Palczewska G., Kiser P. D., Palczewski K. (2019). Photic generation
of 11-*cis*-retinal in bovine retinal pigment epithelium. J. Biol. Chem..

[2] Palczewski K., Kiser P. D. (2020). Shedding new light on the generation
of the visual
chromophore. Proc. Natl. Acad. Sci. U. S. A..

[3] Chen S., Getter T., Salom D., Wu D., Quetschlich D., Chorev D. S., Palczewski K., Robinson C. V. (2022). Capturing a rhodopsin
receptor signalling cascade across a native membrane. Nature.

[4] Gruhl T., Weinert T., Rodrigues M. J., Milne C. J., Ortolani G., Nass K., Nango E., Sen S., Johnson P. J. M., Cirelli C., Furrer A., Mous S., Skopintsev P., James D., Dworkowski F., Båth P., Kekilli D., Ozerov D., Tanaka R., Glover H., Bacellar C., Brünle S., Casadei C. M., Diethelm A. D., Gashi D., Gotthard G., Guixà-González R., Joti Y., Kabanova V., Knopp G., Lesca E., Ma P., Martiel I., Mühle J., Owada S., Pamula F., Sarabi D., Tejero O., Tsai C.-J., Varma N., Wach A., Boutet S., Tono K., Nogly P., Deupi X., Iwata S., Neutze R., Standfuss J., Schertler G., Panneels V. (2023). Ultrafast structural changes direct
the first molecular events of vision. Nature.

[5] Moiseyev G., Nikolaeva O., Chen Y., Farjo K., Takahashi Y., Ma J.-x. (2010). Inhibition of the visual cycle by A2E through direct interaction
with RPE65 and implications in Stargardt disease. Proc. Natl. Acad. Sci. U. S. A..

[6] Sparrow J. R., Gregory-Roberts E., Yamamoto K., Blonska A., Ghosh S. K., Ueda K., Zhou J. (2012). The bisretinoids of retinal pigment
epithelium. Prog. Ret. Eye Res..

[7] Kim H. J., Sparrow J. R. (2021). Bisretinoid phospholipid
and vitamin A aldehyde: shining
light. J. Lipid Res..

[8] Mata N. L., Weng J., Travis G. H. (2000). Biosynthesis
of a major lipofuscin
fluorophore in mice and humans with ABCR-mediated retinal and macular
degeneration. Proc. Natl. Acad. Sci. U. S. A..

[9] Yakovleva M. A., Radchenko A. S., Feldman T. B., Kostyukov A. A., Arbukhanova P. M., Borzenok S. A., Kuzmin V. A., Ostrovsky M. A. (2020). Fluorescence
characteristics of lipofuscin fluorophores from human retinal pigment
epithelium. Photochem. Photobiol. Sci..

[10] Getter T., Suh S., Hoang T., Handa J. T., Dong Z., Ma X., Chen Y., Blackshaw S., Palczewski K. (2019). The selective
estrogen receptor modulator raloxifene mitigates the effect of all-*trans*-retinal toxicity in photoreceptor degeneration. J. Biol. Chem..

[11] Sakai N., Decatur J., Nakanishi K., Eldred G. E. (1996). Ocular Eye Pigment
“A2E”: An Unprecedented Pyridinium Bisretinoid. J. Am. Chem. Soc..

[12] Sparrow J. R., Fishkin N., Zhou J., Cai B., Jang Y. P., Krane S., Itagaki Y., Nakanishi K. (2003). A2E, a byproduct
of the visual cycle. Vision Res..

[13] Parish C. A., Hashimoto M., Nakanishi K., Dillon J., Sparrow J. (1998). Isolation
and one-step preparation of A2E and iso-A2E, fluorophores from human
retinal pigment epithelium. Proc. Natl. Acad.
Sci. U. S. A..

[14] Li J., Yao K., Yu X., Dong X., Gan L., Luo C., Wu Y. (2013). Identification
of a Novel Lipofuscin Pigment (iisoA2E) in Retina
and Its Effects in the Retinal Pigment Epithelial Cells. J. Biol. Chem..

[15] Ren R. X.-F., Sakai N., Nakanishi K. (1997). Total Synthesis of the Ocular Age
Pigment A2E: A Convergent Pathway. J. Am. Chem.
Soc..

[16] The numbering of the unsaturated branches used in ref 14 is the opposite to those of refs 11–13.

[17] Alvarez R., de Lera A. R. (2021). Natural polyenic
macrolactams and polycyclic derivatives
generated by transannular pericyclic reactions: optimized biogenesis
challenging chemical synthesis. Nat. Prod. Rep..

[18] Rivas A., Castiñeira M., Álvarez R., Vaz B., de Lera A. R. (2022). Stereoselective
Synthesis of Bisfuranoxide (Aurochrome, Auroxanthin) and Monofuranoxide
(Equinenone 5′,8′-Epoxide) Carotenoids by Double Horner–Wadsworth–Emmons
Reaction. J. Nat. Prod..

[19] Iglesias-Menduiña O., Novegil D., Martínez C., Alvarez R., de Lera A. R. (2024). From Acyclic
Intramolecular-[4 + 2]- to Transannular Bis-[4 + 2]-Cycloaddition
of the Macrodiolide for the Stereoselective Synthesis of the Octahydronaphthalene
Core of Polyenic Macrolactam Sagamilactam. Org.
Lett..

[20] Iglesias-Menduiña O., Martínez C., Vaz B., Alvarez S., Alvarez R., de Lera A. R. (2025). DP4+-Based Stereochemical Reassignment and Total Synthesis
of Polyenic Macrolactam Muanlactam. J. Org.
Chem..

[21] Horner L. (1964). Darstellung
und Eigenschaften optisch aktiver, tertiärer Phosphine. Pure Appl. Chem..

[22] Wadsworth W. S. (1977). Synthetic
Applications of Phosphoryl-Stabilized Anions. Org. React..

[23] Nicolaou K. C., Härter M. W., Gunzner J. L., Nadin A. (1997). The Wittig and Related
Reactions in Natural Product Synthesis. Liebigs
Ann..

[24] Gu, Y. ; Tian, S.-K. Olefination Reactions of Phosphorus-Stabilized Carbon Nucleophiles. In Stereoselective Alkene Synthesis; Wang, J. , Ed.; Springer: Berlin, 2012; Vol. 327, pp 197–238. 10.1007/128_2012_314 22371171

[25] Kobayashi K., Tanaka K., Kogen H. (2018). Recent topics of the natural product
synthesis by Horner–Wadsworth–Emmons reaction. Tetrahedron Lett..

[26] Roman D., Sauer M., Beemelmanns C. (2021). Applications
of the Horner–Wadsworth–Emmons
Olefination in Modern Natural Product Synthesis. Synthesis.

[27] Azim E.-M., Auzeloux P., Maurizis J.-C., Braesco V., Grolier P., Veyre A., Madelmont J.-C. (1996). Synthesis of all-*trans-*beta-carotene, retinoids and derivatives labeled with ^14^C. J. Labelled Compd. Radiopharm..

[28] Bulger P. G., Moloney M. G., Trippier P. C. (2003). A multicomponent
coupling strategy
suitable for the synthesis of the triene component of the oxazolomycin
antibiotics. Org. Biomol. Chem..

[29] Zhang L.-Z., Zhang P.-C., Wang Q., Zhou M., Zhang J. (2025). Enantioselective
Heck/Tsuji–Trost reaction of flexible vinylic halides with
1,3-dienes. Nat. Commun..

[30] Hiroya K., Takuma K., Inamoto K., Sakamoto T. (2009). Synthesis
of 6z-pandanamine
by regioselective cyclization reaction of 2-en-4-ynoic acid derivatives
promoted by weak base. Heterocycles.

[31] Del
Valle D. J., Krische M. J. (2013). Total Synthesis of (+)-Trienomycins
A and F via C–C Bond-Forming Hydrogenation and Transfer Hydrogenation. J. Am. Chem. Soc..

[32] Setterholm N. A., McDonald F. E. (2018). Stereoselective Synthesis of Pyrans from Epoxyalkenes:
Dual Catalysis with Palladium and Brønsted Acid. J. Org. Chem..

[33] Duboudin J. G., Jousseaume B., Bonakdar A., Saux A. (1979). Reactifs de grignard
vinyliques γ fonctionnels: II. Iodolyse, alkylation et arylation
des iodo-alcools. J. Organomet. Chem..

[34] Rivas A., Pequerul R., Barracco V., Domínguez M., López S., Jiménez R., Parés X., Alvarez R., Farrés J., de Lera A. R. (2020). Synthesis of C11-to-C14
methyl-shifted all-*trans*-retinal analogues and their
activities on human aldo-keto reductases. Org.
Biomol. Chem..

[35] Farina, V. ; Roth, G. P. Recent Advances in the Stille Reaction; JAI Press: Greenwich, CT, 1996; Vol. 5, pp 1–53.

[36] Farina, V. ; Krishnamurthy, W. K. ; Scott, W. K. Organic Reactions; Wiley: New York, 1997; Vol. 50.

[37] Espinet P., Echavarren A. M. (2004). The Mechanisms
of the Stille Reaction. Angew. Chem., Int. Ed..

[38] Heravi M. M., Hashemi E., Azimian F. (2014). Recent developments
of the Stille
reaction as a revolutionized method in total synthesis. Tetrahedron.

[39] Alternative reaction conditions using anion generation with NaHMDS, DMPU, and THF or *n*-BuLi, DMPU, and THF and condensation with **17** afforded lower stereoselectivities (2:1 ratio).

[40] Miyaura N., Suzuki A. (1995). Palladium-Catalyzed Cross-Coupling Reactions of Organoboron
Compounds. Chem. Rev..

[41] Suzuki A. (2005). Carbon-carbon
bonding made easily. Chem. Commun..

[42] Suzuki A. (2011). Cross-Coupling
Reactions Of Organoboranes: An Easy Way To Construct C-C Bonds (Nobel
Lecture). Angew. Chem., Int. Ed..

[43] Coombs J. R., Zhang L., Morken J. P. (2015). Synthesis
of Vinyl Boronates from
Aldehydes by a Practical Boron–Wittig Reaction. Org. Lett..

[44] Rivas A., Pérez-Revenga V., Alvarez R., de Lera A. R. (2019). Bidirectional Hiyama–Denmark
Cross-Coupling Reactions of Bissilyldeca-1,3,5,7,9-pentaenes for the
Synthesis of Symmetrical and Non-Symmetrical Carotenoids. Chem. - Eur. J..

[45] Liu R. S. H., Asato A. E. (1984). Photochemistry and synthesis of stereoisomers of vitamin
A. Tetrafedron.

[46] Scarlato G. R., DeMattei J. A., Chong L. S., Ogawa A. K., Lin M. R., Armstrong R. W. (1996). Asymmetric
Synthesis of Calyculin C. 1. Synthesis of
the C1–C25 Fragment. J. Org. Chem..

[47] Jin B., Liu Q., Sulikowski G. A. (2005). Development
of an end-game strategy
towards apoptolidin: a sequential Suzuki coupling approach. Tetrahedron.

[48] Acemoglu M., Prewo R., Bieri J. H., Eugster C. H. (1984). (6′*RS*,8′*RS*,2*E*)- und
(6′*RS*,8′*SR*,2*E*)-3-Methyl-3-(2′,2′,6′-trimethyl-7′-oxabicyclo­[4.3.0]­non-9′-en-8′-yl)-2-propenal
([(5*RS*,8*RS*)- und (5*RS*,8*SR*)-5,8-Epoxy-5,8-dihydro-ionyliden]­acetaldehyd):
Synthese und Röntgenstrukturanalyse. Helv. Chim. Acta.

[49] Acemoglu M., Eugster C. H. (1984). (5*R*,6*S*,5′*R*,6′*S*)-5,6,5′,6′-Diepoxy-β,β-cartin:
Synthese, Spectroskopische, chiroptische und chromatographische Eigenschaften. Helv. Chim. Acta.

[50] Nandgaye D. C., Daf A. N., Lade U. B., Moharkar D. W. (2023). The Woodward
Fisher
Regulation for Calculating Absorption Maxima. Int. J. Pharm. Biomed. Sci..

[51] Wu Y., Jin Q., Yao K., Zhao J., Chen J., Wu X., Gan L., Li J., Song X., Liu X., Cai X. (2014). Retinal metabolism
in humans induces the formation of an unprecedented lipofuscin fluorophore
‘pdA2E’. Biochem. J..

[52] Cheng K.-J., Hsieh C.-M., Nepali K., Liou J.-P. (2020). Ocular Disease Therapeutics:
Design and Delivery of Drugs for Diseases of the Eye. J. Med. Chem..

