# Supplementary material for: Stereochemical Reassignment by Total Synthesis of an Ocular Pyridinium Bisretinoid of Retinal Pigment Epithelium Lipofuscin: iiso-A2E Is i‑A2E
Source: Org Lett. 2025 Dec 4;27(50):14070–5. doi: 10.1021/acs.orglett.5c04694 (PMC12723671; doi:10.1021/acs.orglett.5c04694)

Supporting information (S.I.)

Stereochemical Reassignment by Total Synthesis of an Ocular Pyridinium Bisretinoid of Retinal Pigment Epithelium Lipofuscin: *iiso*-A2E is *i*-A2E

Brais Vidal, Oscar Iglesias-Menduiña, Belén Vaz, Rosana Álvarez, * Claudio Martínez,* and Ángel R. de Lera*

CINBIO, Departamento de Química Orgánica, Universidade de Vigo, IBIV, Lagoas-Marcosende, 36310 Vigo, Spain

(qolera@uvigo.gal; rar@uvigo.gal; claudiom@uvigo.gal)

Table of Contents

1. General remarks 2
2. Experimental procedures 3
3. Comparison of the ^{1}H-NMR spectra of *iiso*-A2E (**8**) (natural),[4] *iiso*-A2E (**8**) (synthetic) and *i*-A2E **9**. . 21
4. UV spectra 25
5. References 26
6. Copies of NMR spectra 27

1. General remarks

Solvents were dried using a Puresolv^{TM} solvent purification system. All other reagents were commercial compounds of the highest purity available. If not specified, all reactions were carried out under an argon atmosphere. Those not involving aqueous reagents were carried out in oven dried glassware. For reactions that require heating, a metallic heating block was used and the indicated is the external temperature. All solvents and anhydrous solutions were transferred through syringes and cannulas previously dried in the oven for at least 12h and kept in a desiccator. Analytical TLC was performed on aluminium plates with Merck Kieselgel $60F_{254}$ and visualized by UV irradiation (254 nm) or by staining with a solution of phosphomolybdic acid in ethanol. Flash column chromatography was carried out using Merck Kieselgel 60 (230–400 mesh) with a CombiFlash® Rf Teledyne Isco.

HRMS (ESI$^+$) were measured with an FT-ICR-MS Solarix 7T mass spectrometer (Bruker Daltonics). ^{1}H-NMR spectra were recorded in $CDCl_3$, C_6D_6 and CD_3OD at 298 K with a Bruker AMX-400 spectrometer at 400.16 MHz with residual protic solvent as the internal reference [$CDCl_3$, δ= 7.26 ppm, C_6D_6, δ= 7.16 ppm; CD_3OD = 3.31 ppm]; chemical shifts (δ) are given in parts per million (ppm) and coupling constants (*J*) are given in Hertz (Hz). The proton spectra are reported as follows: δ (multiplicity, coupling constant *J*, number of protons). ^{13}C-NMR spectra were recorded in $CDCl_3$, C_6D_6, and CD_3OD at 298 K with the same spectrometer operating at 100.63 MHz with the central peak of $CDCl_3$ (δ = 77.26 ppm), C_6D_6 (δ = 128.06 ppm), and CD_3OD (δ = 49.0 ppm) as the internal reference. DEPT-135 pulse sequences and HSQC bidimensional NMR spectra were used to aid in the assignment of signals in the ^{13}C- and ^{1}H-NMR spectra. NOE-1D experiments were also performed in selected cases. UV/Vis spectra were recorded on a Cary 100 Bio spectrophotometer. Infrared spectra (IR) were obtained on a JASCO FT/IR-4200 infrared spectrometer, from a thin film deposited onto a NaCl glass. IR data include only characteristic absorptions. Peaks are quoted in wave numbers (cm^{-1}), and their relative intensities are reported as follows: s = strong, m = medium, w = weak. Specific optical rotations were measured on a JASCO P-1020 polarimeter with a Na lamp (glass cell, 3.5 x 100 mm). Fluorescence emission spectra were measured using a JASCO FP-8550 spectrofluorometer with the excitation wavelength (λ_{ex}) at 430 nm.

2. Experimental Procedures

(*Z*)-*tert*-Butyl((3-iodo-2-methylallyl)oxy)dimethylsilane 14.

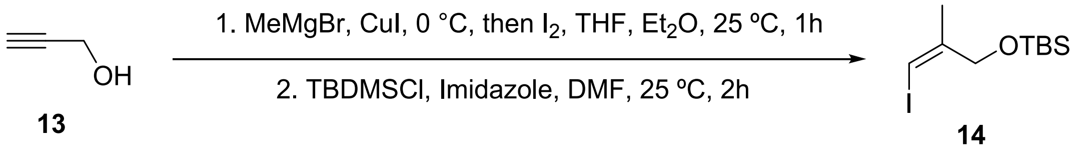

13 **14**

To a cooled (0 °C) stirred solution of CuI (0.29 g, 1.52 mmol) in THF (15 mL), MeMgBr (9.9 mL, 29.72 mmol) was added. Then, prop-2-yn-1-ol **13** (0.90 mL, 15.16 mmol) was added. The mixture was stirred at 0 °C for 45 min, whereupon a solution of I_2 (3.81 g, 15.01 mmol) in Et_2O (25 mL) was added. The mixture was stirred for 1h at 25 °C. A saturated aqueous solution of NH_4Cl was added and the mixture was extracted with Et_2O (3x). The combined organic layers were washed with a saturated aqueous solution of $Na_2S_2O_3$ and brine, dried over anhydrous Na_2SO_4, filtered and the solvent was evaporated.

Imidazole (1.29 g, 18.95 mmol) and TBDMSCl (1.71 g, 11.37 mmol) were added to a solution of the crude obtained above in DMF (14 mL) and the mixture was stirred for 2h at room temperature. A saturated aqueous solution of $NaHCO_3$ was added and the mixture was extracted with Et_2O (3x). The combined organic layers were washed with brine, dried over Na_2SO_4, filtered and the solvent was evaporated. The residue was purified by flash column chromatography (silica gel, 98:2 *v/v* *n*-hexane/Et_3N; then, from 100:0 to 95:5 *v/v* *n*-hexane/EtOAc) to afford 1.85 g (39% yield) of a colourless oil, which was identified as (*Z*)-*tert*-butyl((3-iodo-2-methylallyl)oxy)dimethylsilane **14**. The spectroscopic data of the obtained compound matched those for the same product previously reported in the literature.[1,2]

(*Z*)-*tert*-Butyldimethyl((2-methyl-3-(tributylstannyl)allyl)oxy)silane 15.

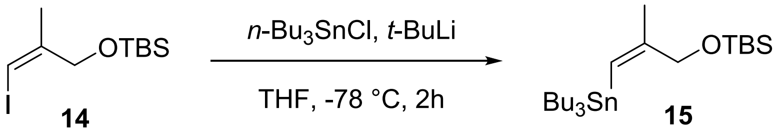

14 **15**

To a stirred solution of (*Z*)-*tert*-butyl((3-iodo-2-methylallyl)oxy)dimethylsilane **14** (1.85 g, 5.93 mmol) in THF (56 mL), *n*-Bu_3SnCl (2.5 mL, 8.90 mmol) was added. The reaction flask was cooled down to -78 °C, *t*-BuLi (7.0 mL, 11.86 mmol) was added and the mixture

was stirred for 2h. A saturated aqueous solution of $NaHCO_3$ was added and the mixture was extracted with Et_2O (3x). The combined organic layers were washed with brine, dried over Na_2SO_4, filtered and the solvent was evaporated. The residue was purified by flash column chromatography (C18 silica gel, from 100:0 to 70:30 *v/v* CH_3CN/CH_2Cl_2) to afford 2.29 g (84% yield) of a colourless oil, which was identified as (*Z*)-*tert*-butyldimethyl((2-methyl-3-(tributylstannyl)allyl)oxy)silane **15**. The spectroscopic data of the obtained compound matched those for the same product previously reported in the literature.[2]

(*Z*)-3-(3-((*tert*-Butyldimethylsilyl)oxy)-2-methylprop-1-en-1-yl)isonicotinaldehyde 17.

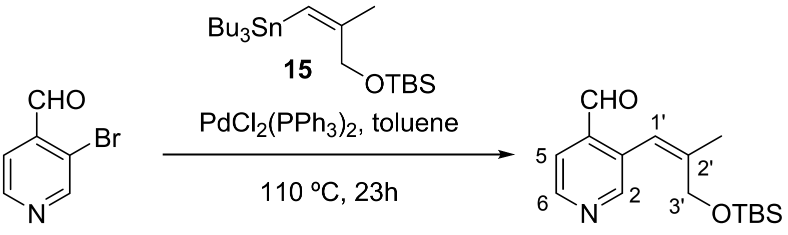

16 **17**

To a degassed solution of 3-bromoisonicotinaldehyde **16** (0.3 g, 1.61 mmol) and (*Z*)-*tert*-butyldimethyl((2-methyl-3-(tributylstannyl)allyl)oxy)silane **15** (1.15 g, 2.42 mmol) in toluene (10 mL), $PdCl_2(PPh_3)_2$ (45.3 mg, 0.06 mmol) was added. The mixture was stirred at 110 °C for 23h. A saturated aqueous solution of NH_4Cl was added and the mixture was extracted with EtOAc (3x). The combined organic layers were dried over Na_2SO_4, filtered and the solvent was evaporated. The residue was purified by flash column chromatography (silica gel, 98:2 *v/v* *n*-hexane/Et_3N; then, from 100:0 to 90:10 *v/v* *n*-hexane/EtOAc) to afford 0.42 g (90% yield) of a colourless oil, which was identified as **17**.

^{1}H-NMR (400.16 MHz, $CDCl_3$): δ 10.24 (s, 1H, CHO), 8.65 (d, *J* = 5.0 Hz, 1H, H_6), 8.57 (s, 1H, H_2), 7.61 (d, *J* = 5.0 Hz, 1H, H_5), 6.56 (s, 1H, $H_{1'}$), 4.04 (s, 2H, 2x$H_{3'}$), 2.00 (s, 3H, CH_3), 0.80 (s, 9H, SiC($\underline{CH_3}$)$_3$), -0.08 (s, 6H, 2xSi-CH_3) ppm. **^{13}C-NMR** (100.63 MHz, $CDCl_3$): δ 191.7 (d), 152.5 (d), 149.0 (d), 144.3 (s), 138.6 (s), 133.8 (s), 120.2 (d), 118.9 (d), 62.6 (t), 25.8 (q, 3x), 21.7 (q), 18.3 (s), -5.3 (q, 2x) ppm. **IR** (NaCl): υ 2954 (s, C-H), 2928 (s, C-H), 2884 (s, C-H), 2856 (s, C-H), 1707 (s, C=O), 1462 (w), 1253 (w), 1082 (s), 837 (s) cm^{-1}. **HRMS** (ESI$^+$): Calcd for $C_{16}H_{26}NO_2Si$ ($[M+H]^+$), 292.1727; found, 292.1726.

Compounds 18 and 19.

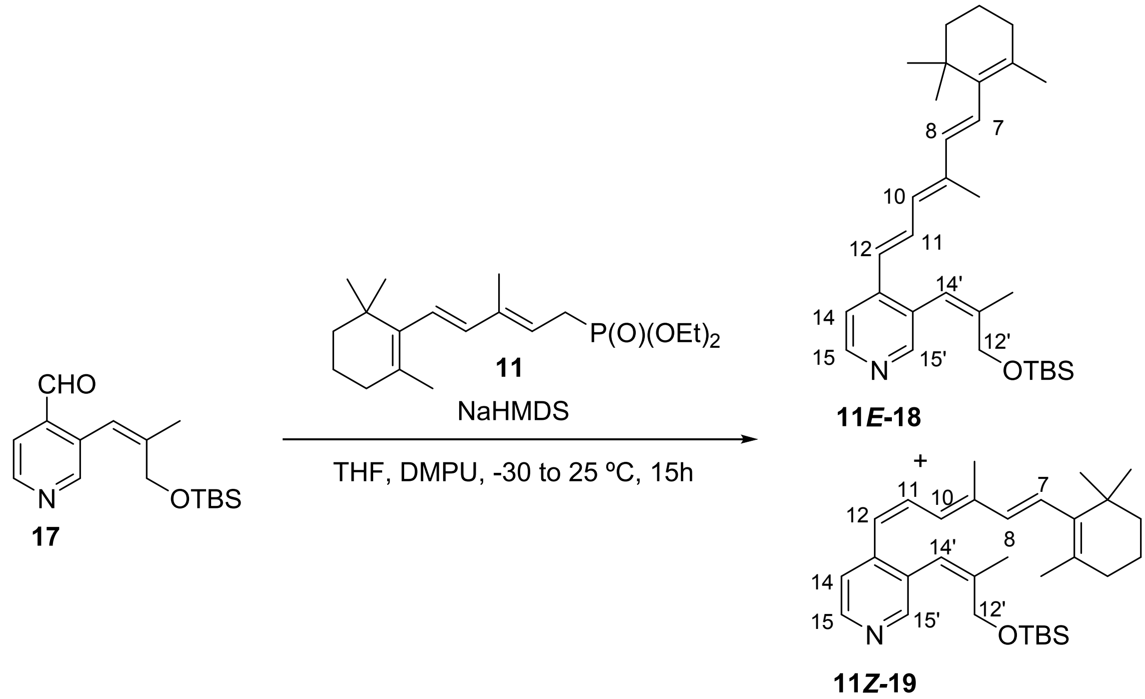

To a cooled (-30 °C) stirred solution of phosphonate **11** (0.18 g, 0.52 mmol) in THF (2 mL), DMPU (0.77 mL) and NaHMDS (0.5 mL, 1M in THF, 0.5 mmol) were added. Subsequently, **17** (90 mg, 0.31 mmol) in THF (2 mL) was added at -30 °C and the mixture was stirred at 25 °C for 3h. A saturated aqueous solution of NH_4Cl was added and the mixture was extracted with Et_2O (3x). The combined organic layers were washed with brine, dried over Na_2SO_4, filtered and the solvent was evaporated. The residue was purified by flash column chromatography (silica gel, 98:2 *v/v n*-hexane/Et_3N; then, from 100:0 to 90:10 *v/v n*-hexane/EtOAc) to afford 83.1 mg (57% yield) of a dark yellow oil, which was identified as **18** and 43.3 mg (29% yield) of a yellow oil, which was identified as **19**.

Data for **18**: **^{1}H-NMR** (400.16 MHz, C_6D_6): δ 8.64 (s, 1H, $H_{15'}$), 8.49 (d, *J* = 5.2 Hz, 1H, H_{15}), 7.23 (dd, *J* = 15.4, 11.4 Hz, 1H, H_{11}), 7.07 (d, *J* = 5.2 Hz, 1H, H_{14}), 6.67 (d, *J* = 15.4 Hz, 1H, H_{12}), 6.36 (d, *J* = 16.1 Hz, 1H, H_7), 6.28 (d, *J* = 16.1 Hz, 1H, H_8), 6.22 (s, 1H, $H_{14'}$), 6.18 (d, *J* = 11.3 Hz, 1H, H_{10}), 4.12 (s, 2H, 2x$CH_{12'}$), 2.03 – 1.94 (m, 5H, CH_2 + CH_3), 1.88 (d, *J* = 1.1 Hz, 3H, CH_3), 1.78 (d, *J* = 0.9 Hz, 3H, CH_3), 1.63 – 1.53 (m, 2H, CH_2), 1.51 – 1.42 (m, 2H, CH_2), 1.13 (s, 6H, 2xCH_3), 0.90 (s, 9H, SiC($\underline{CH_3}$)$_3$), -0.06 (s, 6H, 2xSi-CH_3) ppm. **^{13}C-NMR** (100.63 MHz, C_6D_6): δ 151.9 (d), 148.9 (d), 143.0 (s), 141.3 (s), 138.9 (s), 138.1 (s), 138.0

(d), 131.0 (s), 130.6 (d), 130.3 (d), 130.0 (s), 128.6 (d, 2x), 122.3 (d), 118.8 (d), 62.7 (t), 40.0 (t), 34.6 (s), 33.4 (t), 29.2 (q, 2x), 26.1 (q, 3x), 22.0 (q), 21.4 (q), 19.7 (t), 18.5 (s), 12.9 (q), -5.3 (q, 2x). **IR** (NaCl): υ 2954 (s, C-H), 2927 (s, C-H), 2857 (m, C-H), 1577 (m, C=C), 1252 (m, C-O), 1081 (m, Si-O-C) cm^{-1}. **HRMS** (ESI^+): calcd. for $C_{31}H_{48}NOSi$ ($[M+H]^+$), 478.3505; found, 478.3503.

Data for **19**: **^{1}H-NMR** (400.16 MHz, C_6D_6): δ 8.68 (s, 1H, $H_{15'}$), 8.42 (d, J = 5.0 Hz, 1H, H_{15}), 7.10 (d, J = 5.0 Hz, 1H, H_{14}), 6.66 – 6.49 (m, 2H, H_{12} + H_{11}), 6.32 (d, J = 16.1 Hz, 1H, H_7), 6.28 (d, J = 11.1 Hz, 1H, H_{10}), 6.21 – 6.15 (m, 2H, H_8 + $H_{14'}$), 4.12 (s, 2H, $2xH_{12'}$), 1.95 – 1.85 (m, 5H, CH_2 + CH_3), 1.84 (s, 3H, CH_3), 1.67 (s, 3H, CH_3), 1.60 – 1.52 (m, 2H, CH_2), 1.47 – 1.42 (m, 2H, CH_2), 1.07 (s, 6H, $2xCH_3$), 0.90 (s, 9H, $SiC(\underline{CH_3})_3$), -0.05 (s, $2xSi\text{-}CH_3$) ppm. **^{13}C-NMR** (100.63 MHz, C_6D_6): δ 151.3 (d), 148.5 (d), 143.6 (s), 141.2 (s), 139.7 (s), 138.3 (d), 138.1 (s), 132.1 (s), 129.7 (s), 129.1 (d), 128.7 (d), 126.4 (d), 125.9 (d), 123.8 (d), 122.4 (d), 62.8 (t), 39.9 (t), 34.6 (s), 33.2 (t), 29.2 (q), 26.1 (q, 3x), 21.9 (q), 21.6 (q), 19.7 (t), 18.5 (s), 12.6 (q), -5.3 (q, 2x) ppm. **IR** (NaCl): υ 2954 (s, C-H), 2927 (s, C-H), 2856 (s, C-H), 1579 (w), 1470 (w), 1401 (w), 1252 (w), 1081 (m) cm^{-1}. **HRMS** (ESI^+): calcd. for $C_{31}H_{48}NOSi$ ($[M+H]^+$), 478.3505; found, 478.3500.

((*E*)-2-(3-((*Z*)-3-((*tert*-Butyldimethylsilyl)oxy)-2-methylprop-1-en-1-yl)pyridin-4-yl)vinyl)boronic acid 21.

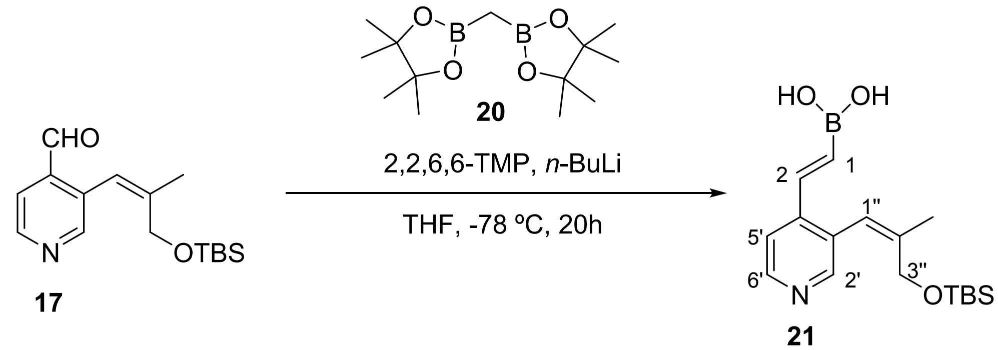

To a cooled (0 °C) solution of 2,2,6,6-tetramethylpiperidine (0.23 mL, 1.37 mmol) in THF (0.5 mL), *n*-BuLi (0.23 mL, 2.0 M in hexanes, 1.37 mmol) was added and the mixture was stirred for 30 min. Then, a solution of bis(pinacolatoboryl)methane **20** (0.37 g, 1.37 mmol) in THF (1 mL) was added and the reaction mixture was stirred for 10 min. The mixture was cooled down to -78 °C, a solution of **17** (0.1 g, 0.34 mmol) in THF (1 mL) was added dropwise, and the reaction mixture was stirred at -78 °C for 20h. A saturated

aqueous solution of NH_4Cl was added and the mixture was extracted with Et_2O (3x). The combined organic layers were washed with brine, dried over anhydrous Na_2SO_4, filtered and the solvent was evaporated. The residue was purified by flash column chromatography (C18 silica gel, from 50:50 to 100:0 *v/v* CH_3OH/H_2O) to afford 70.3 mg (62% yield) of a red foam, which was identified as **21**. **^{1}H-NMR** (400.16 MHz, C_6D_6): δ 8.72 (s, 1H, $H_{2'}$), 8.52 (d, *J* = 5.4 Hz, 1H, $H_{6'}$), 7.79 (d, *J* = 18.0 Hz, 1H, H_2), 7.29 (d, *J* = 5.4 Hz, 1H, $H_{5'}$), 6.83 (d, *J* = 18.0 Hz, 1H, H_1), 6.37 (s, 1H, $H_{1''}$), 4.14 (s, 2H, $2xH_{3''}$), 1.94 (s, 3H, CH_3), 0.87 (s, 9H, $SiC(\underline{CH_3})_3$), -0.06 (s, 6H, $2xSi-CH_3$) ppm. **^{13}C-NMR** (100.63 MHz, C_6D_6): δ 149.6 (d), 146.5 (d), 145.7 (s), 142.6 (s), 140.3 (d) (from HSQC experiments), 137.1 (d) (from HSQC experiments), 132.1 (s), 121.2 (d), 120.2 (d), 62.8 (t), 26.1 (q, 3x), 21.7 (q), 18.5 (s), -5.3 (q, 2x) ppm. **IR** (NaCl): υ 2953 (s, C-H), 2930 (s, C-H), 2856 (m, C-H), 1619 (m, C=C), 1587 (m, C=C), 1405 (s), 1254 (s, C-O), 1082 (s, Si-O-C) cm^{-1}. **HRMS** (ESI^+): calcd. for $C_{17}H_{29}BNO_3Si$ ($[M+H]^+$) 334.2010; found, 334.2005.

Compound 18.

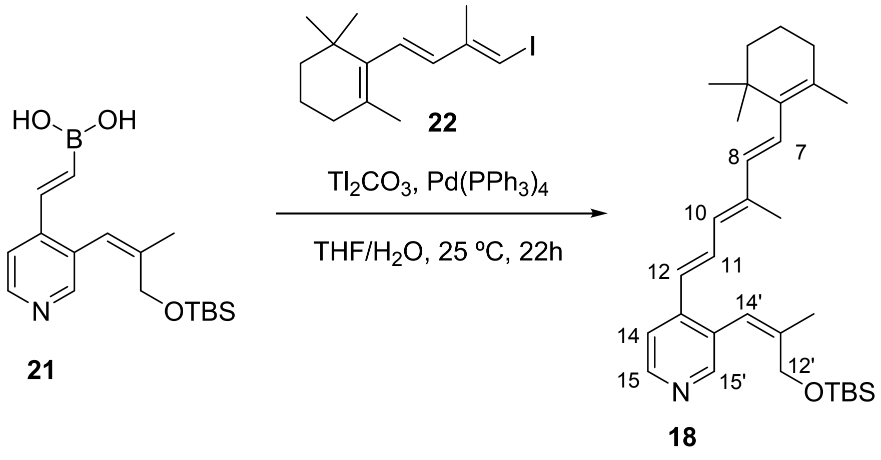

To a cooled (0 °C) solution of 2-((1*E*,3*E*)-4-iodo-3-methylbuta-1,3-dien-1-yl)-1,3,3-trimethylcyclohex-1-ene **22** (45.5 mg, 0.14 mmol) and alkenyl boronic acid **21** (40.0 mg, 0.12 mmol) in a THF-water mixture (4 mL, 4:1 *v/v*, Tl_2CO_3 (0.14 g, 0.30 mmol) and $Pd(PPh_3)_4$ (6.90 mg, 0.01 mmol) were added, and the reaction mixture was stirred at 25 °C for 22h. The mixture was filtered through Celite® and Na_2SO_4 washing with Et_2O and the solvent was evaporated. The residue was purified by flash column chromatography (silica gel, 98:2 *v/v* *n*-hexane/Et_3N; then, from 100:0 to 90:10 *v/v* *n*-hexane/EtOAc) to afford 42.3 mg (74% yield) of a dark yellow oil, which was identified as **18**.

Compound 23.

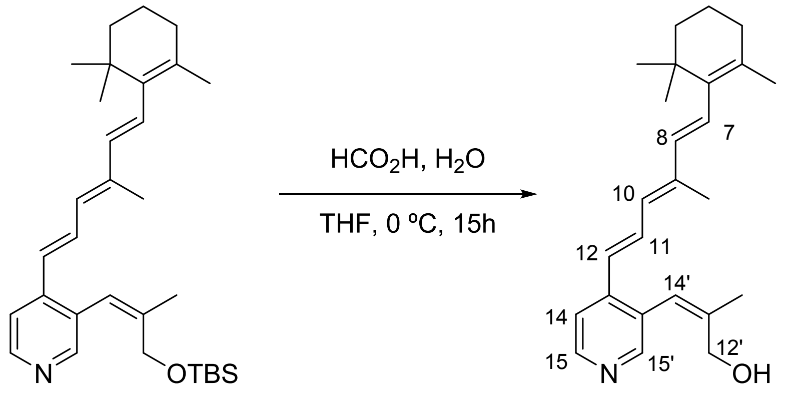

18 **23**

A cooled (0 °C) mixture of HCO_2H (4.8 mL, 126.28 mmol) and H_2O (1.6 mL, 86.52 mmol) was added to a cooled (0 °C) solution of **18** (170 mg, 0.37 mmol) in THF (10 mL). The mixture was stirred at 0 °C for 15h. A saturated aqueous solution of $NaHCO_3$ was then added and the mixture was extracted with EtOAc (3x). The combined organic layers were dried over Na_2SO_4, filtered and the solvent was evaporated. The residue was purified by flash column chromatography (silica gel, 98:2 *v/v* *n*-hexane/Et_3N; then, from 70:30 to 50:50 *v/v* *n*-hexane/EtOAc) to afford 68.2 mg (52% yield) of a dark red oil, which was identified as **23**. **^{1}H-NMR** (400.16 MHz, C_6D_6): δ 8.62 (br s, 1H, $H_{15'}$), 8.40 (br s, 1H, H_{15}), 7.19 (dd, *J* = 15.4, 11.4 Hz, 1H, H_{11}), 7.02 (d, *J* = 5.1 Hz, 1H, H_{14}), 6.60 (d, *J* = 15.4 Hz, 1H, H_{12}), 6.38 (d, *J* = 16.1 Hz, 1H, H_7), 6.27 (d, *J* = 16.1 Hz, 1H, H_8), 6.19 – 6.11 (m, 2H, $H_{14'}$ + H_{10}), 4.00 (s, 2H, CH_2), 2.06 – 1.93 (m, 5H, CH_2 + CH_3), 1.87 (d, *J* = 1.1 Hz, 3H, CH_3), 1.78 (d, *J* = 1.1 Hz, 3H, CH_3), 1.65 – 1.55 (m, 2H, CH_2), 1.52 – 1.44 (m, 2H, CH_2), 1.14 (s, 6H, 2xCH_3) ppm. **^{13}C-NMR** (100.63 MHz, C_6D_6): δ 151.2 (d), 147.9 (d), 143.7 (s), 142.3 (s), 139.3 (s), 138.1 (s), 138.0 (d), 130.7 (d), 130.5 (d), 130.1 (s), 128.8 (d), 128.0 (s), 127.7 (d), 122.0 (d), 119.1 (d), 61.8 (t), 39.9 (t), 34.6 (s), 33.4 (t), 29.2 (q, 2x), 22.0 (q), 21.5 (q), 19.7 (t), 12.9 (q) ppm. **IR** (NaCl): υ 3500 – 3200 (br, O-H), 2924 (s, C-H), 2858 (m, C-H), 1581 (s, C=C) cm^{-1}. **HRMS** (ESI$^+$): calcd. for $C_{25}H_{34}NO$ ($[M+H]^+$) 364.2640; found, 364.2635.

Compound 24.

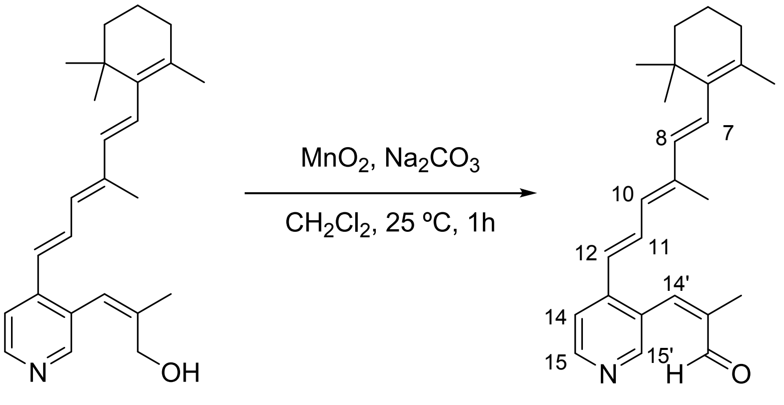

To a stirred solution of **23** (65 mg, 0.18 mmol) in CH_2Cl_2 (5 mL) at room temperature, Na_2CO_3 (341 mg, 3.22 mmol) and MnO_2 (280 mg, 3.22 mmol) were added. The resulting reaction mixture was stirred at room temperature for 1h. The mixture was filtered through Celite® washing with CH_2Cl_2 to afford 55 mg (85% yield) of a dark red oil, which was identified as **24**. **^{1}H-NMR** (400.16 MHz, C_6D_6): δ 9.72 (s, 1H, CHO), 8.45 (d, *J* = 5.3 Hz, 1H, H_{15}), 8.34 (s, 1H, $H_{15'}$), 7.12 (dd, *J* = 15.3, 11.3 Hz, 1H, H_{11}), 7.01 – 6.89 (m, 2H, H_{14} + $H_{14'}$), 6.40 (d, *J* = 16.1 Hz, 1H, H_7), 6.35 (d, *J* = 15.3 Hz, 1H, H_{12}), 6.28 (d, *J* = 16.1 Hz, 1H, H_8), 6.12 (d, *J* = 11.3 Hz, 1H, H_{10}), 1.96 (t, *J* = 6.0 Hz, 2H, CH_2), 1.86 (d, *J* = 1.1 Hz, 3H, CH_3), 1.80 (d, *J* = 1.5 Hz, 3H, CH_3), 1.78 (d, *J* = 1.0 Hz, 3H, CH_3), 1.63 – 1.55 (m, 2H, CH_2), 1.50 – 1.44 (m, 2H, CH_2), 1.13 (s, 6H, 2xCH_3) ppm. **^{13}C-NMR** (100.63 MHz, C_6D_6): δ 190.9 (d), 152.0 (d), 150.2 (d), 143.6 (s), 140.5 (d), 140.2 (s), 139.9 (s), 138.1 (s, 2x), 137.7 (d), 131.8 (d), 130.3 (s), 130.0 (d), 129.5 (d), 126.8 (d), 119.2 (d), 39.9 (t), 34.6 (s), 33.4 (t), 29.2 (q, 2x), 22.0 (q), 19.6 (t), 16.6 (q), 12.9 (q) ppm. **IR** (NaCl): υ 2924 (s, C-H), 2854 (m, C-H), 1679 (s, C=O), 1574 (m, C=C) cm^{-1}. **HRMS** (ESI$^+$): calcd. for $C_{25}H_{32}NO$ ($[M+H]^+$) 362.2484; found, 362.2479.

Compound 10.

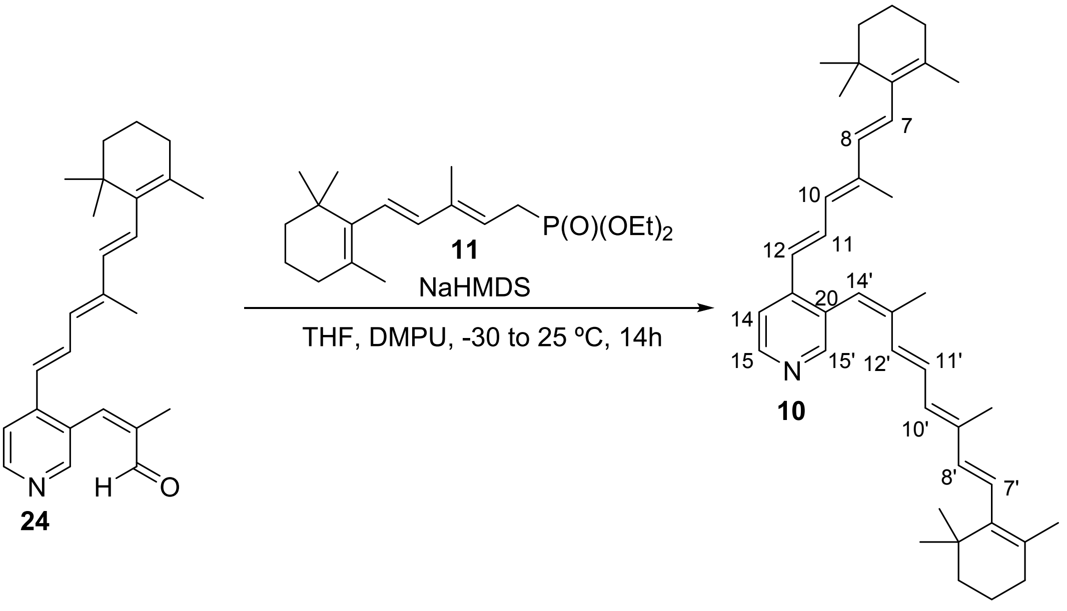

To a cooled (-30 °C) stirred solution of phosphonate **11** (88.0 mg, 0.26 mmol) in THF (0.8 mL), DMPU (0.4 mL) and NaHMDS (0.24 mL, 1M in THF, 0.24 mmol) were added. Subsequently, **24** (55.0 mg, 0.15 mmol) in THF (0.8 mL) was added and the mixture was allowed to warm up to room temperature for 14h. A saturated aqueous solution of NH_4Cl was added and the mixture was extracted with Et_2O (3x). The combined organic layers were washed with brine, dried over Na_2SO_4, filtered and the solvent was evaporated. The residue was purified by flash column chromatography (silica gel, 98:2 *v/v* *n*-hexane/Et_3N; then, from 100:0 to 90:10 *v/v* *n*-hexane/EtOAc) to afford 49.2 mg (60% yield) of a dark red foam, which was identified as **10**. **^{1}H-NMR** (400.16 MHz, C_6D_6): δ 8.81 (s, 1H, $H_{15'}$), 8.54 (d, *J* = 5.3 Hz, 1H, H_{15}), 7.24 (dd, *J* = 15.3, 11.4 Hz, 1H, H_{11}), 7.12 (d, *J* = 5.3 Hz, 1H, H_{14}), 6.81 – 6.72 (m, 2H, $H_{12'}$ + $H_{11'}$), 6.69 (d, *J* = 15.4 Hz, 1H, H_{12}), 6.36 (d, *J* = 16.1 Hz, 1H, H_7), 6.33 – 6.23 (m, 3H, H_8 + $H_{14'}$ + $H_{7'}$), 6.20 – 6.13 (m, 2H, H_{10} + $H_{8'}$), 5.95 (d, *J* = 10.4 Hz, 1H, $H_{10'}$), 2.02 – 1.92 (m, 5H, CH_2 + CH_3), 1.87 (d, *J* = 1.1 Hz, 3H, CH_3), 1.84 (d, *J* = 1.1 Hz, 3H, CH_3), 1.78 (d, *J* = 1.0 Hz, 3H, CH_3), 1.76 (d, *J* = 0.7 Hz, 3H, CH_3), 1.63 – 1.56 (m, 4H, 2xCH_2), 1.51 – 1.43 (m, 4H, 2xCH_2), 1.40 – 1.27 (m, 2H, CH_2), 1.13 (s, 6H, 2xCH_3), 1.12 (s, 6H, 2xCH_3) ppm. **^{13}C-NMR** (100.63 MHz, C_6D_6): δ 152.7 (d), 148.8 (d), 143.3 (s), 139.0 (s), 138.5 (d), 138.2 (s), 138.1 (s), 138.0 (d), 137.8 (s), 136.9 (s), 131.4 (d), 130.6 (d), 130.5 (d), 130.4 (d), 130.0 (s), 129.5 (s), 128.6 (d), 128.5 (d), 128.2 (d), 127.9 (s), 127.2 (d), 125.1 (d), 119.0 (d), 40.0 (t), 39.9 (t), 34.6 (s), 34.5 (s), 33.4 (t), 33.3 (t),

29.2 (q, 4x), 22.0 (q, 2x), 20.7 (q), 19.7 (t), 19.6 (t), 12.9 (q), 12.7 (q) ppm. **IR** (NaCl): υ 2960 (m, C-H), 2926 (m, C-H), 2856 (w, C-H), 1453 (m, C=C) cm^{-1}. **HRMS** (ESI^+): calcd. for $C_{40}H_{54}N$ ($[M+H]^+$) 548.4256; found, 548.4251. **UV** (CH_3OH): λ_{max} 368 nm (ε = 22,000).

***Iiso*-A2E (8).**

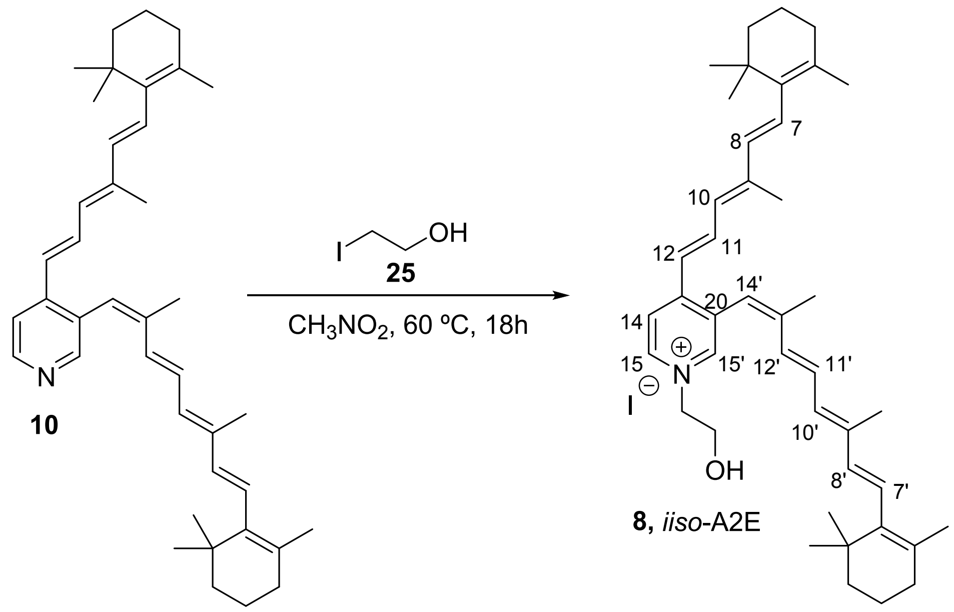

To a solution of **10** (10.0 mg, 0.02 mmol) in CH_3NO_2 (0.3 mL), 2-iodoethanol **25** (0.01 mL, 0.18 mmol) was added. The resulting solution was heated at 60 °C for 18h. The solvent was evaporated under vacuum, the residue was triturated with *n*-hexane and Et_2O mixtures and the solvents were removed to afford 5.7 mg (45% yield) of a dark red solid, which was identified as *iiso*-A2E (**8)**. **^{1}H-NMR** (400.16 MHz, CD_3OD): δ 8.51 (d, *J* = 6.8 Hz, 1H, H_{15}), 8.48 (s, 1H, $H_{15'}$), 8.30 (d, *J* = 6.8 Hz, 1H, H_{14}), 7.97 (dd, *J* = 15.1, 11.6 Hz, 1H, H_{11}), 7.01 (dd, *J* = 15.0, 11.4 Hz, 1H, $H_{11'}$), 6.79 (d, *J* = 15.1 Hz, 1H, H_{12}), 6.55 (d, *J* = 16.1 Hz, 1H, H_7), 6.48 (d, *J* = 15.1 Hz, 1H, $H_{12'}$), 6.45 – 6.41 (m, 2H, H_{10} + $H_{14'}$), 6.34 – 6.25 (m, 2H, H_8 + $H_{8'}$), 6.13 – 6.06 (m, 2H, $H_{7'}$ + $H_{10'}$), 4.55 (t, *J* = 5.4 Hz, 2H, CH_2), 3.98 (t, *J* = 5.4 Hz, 2H, CH_2), 2.23 (d, *J* = 1.4 Hz, 3H, CH_3), 2.17 (s, 3H, CH_3), 2.13 – 2.02 (m, 4H, 2xCH_2), 2.00 (s, 3H, CH_3), 1.74 (d, *J* = 0.9 Hz, 3H, CH_3), 1.67 (s, 3H, CH_3), 1.66 – 1.57 (m, 4H, 2xCH_2), 1.59 – 1.44 (m, 2H, CH_2), 1.34 – 1.27 (m, 2H, CH_2), 1.06 (s, 6H, 2xCH_3), 1.01 (s, 6H, 2xCH_3) ppm. **^{13}C-NMR** (100.63 MHz, CD_3OD): δ 153.6 (s), 147.0 (s), 146.7 (d), 143.1 (s), 141.8 (d), 139.9 (s), 139.8 (d), 139.1 (s), 139.0 (d), 138.9 (s), 138.4 (d), 134.9 (s), 133.1 (d), 132.3 (s), 131.9 (d), 131.2 (d), 130.9 (d), 130.6 (s), 129.2 (d), 129.1 (d), 124.9 (d), 123.0 (d), 120.6 (d), 63.6 (t), 61.8 (t), 40.8 (t), 40.7 (t), 35.3 (s), 35.2 (s), 34.1 (t), 34.0 (t), 29.4 (q,

4x), 22.0 (q), 21.9 (q), 20.8 (q), 20.3 (t), 20.2 (t), 13.2 (q), 12.8 (q) ppm. **HRMS** (ESI^+): calcd. for $C_{42}H_{58}NO^+$ ([M^+]) 592.4513; found, 592.4512. **UV** (CH_3OH): λ_{max} 340 nm (ε = 36,200), 430 nm (ε = 37,200). **FES** (430 nm) = λ_{max} 532 nm.

(*E*)-*tert*-Butyldimethyl((2-methyl-3-(4,4,5,5-tetramethyl-1,3,2-dioxaborolan-2-yl)allyl)oxy)silane 28.

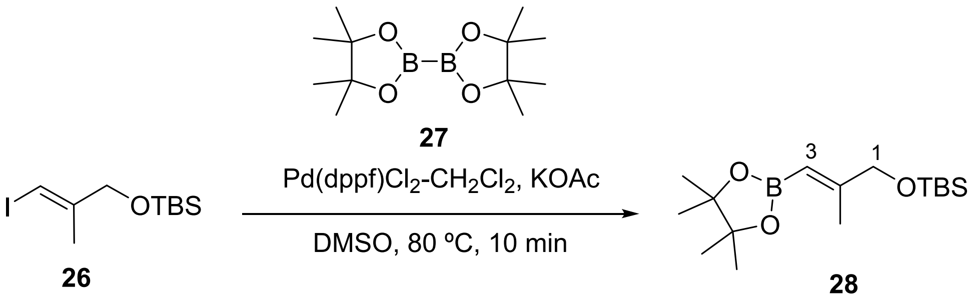

26 **28**

To a mixture of $Pd(dppf)Cl_2{\cdot}CH_2Cl_2$ (0.08 g, 0.1 mmol), KOAc (0.94 g, 9.6 mmol) and bis(pinacolato)diboron **27** (2.4 g, 9.6 mmol), (*E*)-*tert*-butyl-(3-iodo-2-methylallyl)oxy-dimethylsilane **26** [3] (1.0 g, 3.2 mmol) in DMSO (11 mL) was added and the solution was stirred at 80 °C for 10 min. The reaction mixture was cooled down to room temperature and then extracted with Et_2O (3x). The combined organic layers were washed with brine, dried over Na_2SO_4, filtered and the solvent was evaporated. The residue was purified by flash column chromatography (silica gel, from 100:0 to 90:10 *v/v* *n*-hexane/EtOAc) to afford 0.8 g (78% yield) of a colourless oil, which was identified as (*E*)-*tert*-butyldimethyl((2-methyl-3-(4,4,5,5-tetramethyl-1,3,2-dioxaborolan-2-yl)allyl)oxy)silane **28**. **^{1}H-NMR** (400.16 MHz, C_6D_6): δ 6.02 (s, 1H, H_3), 4.00 (s, 2H, $2H_1$), 2.10 (s, 3H, CH_3), 1.09 (s, 12H, 2xO-$C(CH_3)_2$), 0.95 (s, 9H, $SiC(\underline{CH_3})_3$), 0.03 (s, 6H, 2xSi-CH_3) ppm. **^{13}C-NMR** (100.63 MHz, C_6D_6): δ 160.2 (s), 111.0 (d) (from HSQC experiments), 82.6 (d), 68.7 (t), 26.1 (q, 3x), 25.0 (q, 4x), 18.6 (s, 2x), 17.8 (q), -5.3 (q, 2x) ppm. **IR** (NaCl): υ 2978 (w, C-H), 2956 (w, C-H), 2930 (w, C-H), 2857 (w, C-H), 1650 (m, C=C), 1335 (s, B-O), 1258 (m, C-O), 1115 (s, Si-O-C) cm^{-1}. **HRMS** (ESI^+): calcd. for $C_{16}H_{33}BO_3SiNa$ ($[M+Na]^+$), 335.2190; found, 335.2186.

(*E*)-3-(3-((*tert*-Butyldimethylsilyl)oxy)-2-methylprop-1-en-1-yl)isonicotinaldehyde 29.

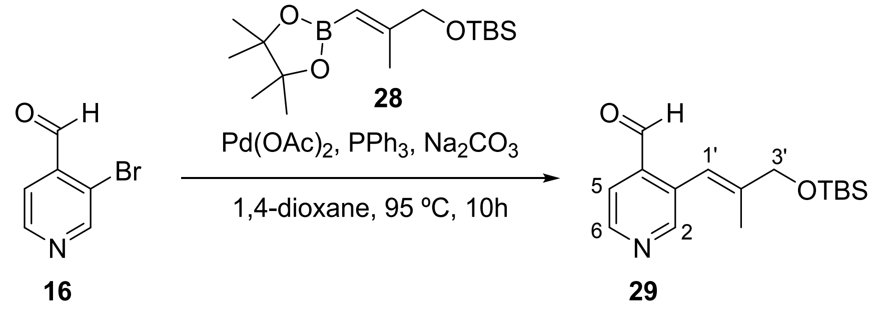

$Pd(OAc)_2$ (24.1 mg, 0.11 mmol), PPh_3 (77.6 mg, 0.30 mmol) and 3-bromoisonicotinaldehyde **16** (0.22 g, 1.18 mmol) were added to a sealing tube. Then, **28** (0.63 g, 2.01 mmol) in 1,4-dioxane (11 mL) and Na_2CO_3 (1.2 mL, 2M, 2.42 mmol) were added, the tube was sealed and the reaction mixture was stirred at 95 °C for 10h. The flask was cooled down to room temperature and the mixture was extracted with CH_2Cl_2 (3x). The combined organic layers were dried over Na_2SO_4, filtered and the solvent was evaporated. The residue was purified by flash column chromatography (silica gel, 98:2 *v/v* *n*-hexane/Et_3N; then, from 100:0 to 90:10 *v/v* *n*-hexane/EtOAc) to afford 0.23 g (66% yield) of a white solid, which was identified as **29**. **^{1}H-NMR** (400.16 MHz, C_6D_6): δ 9.95 (s, 1H, CHO), 8.63 (s, 1H, H_2), 8.45 (d, *J* = 4.9 Hz, 1H, H_6), 7.27 (d, *J* = 4.9 Hz, 1H, H_5), 6.71 (s, 1H, $H_{1'}$), 3.92 (d, *J* = 1.8 Hz, 2H, 2x$H_{3'}$), 1.59 – 1.49 (m, 3H, CH_3), 0.99 (s, 9H, SiC($\underline{CH_3}$)$_3$), 0.06 (s, 6H, 2xSi-CH_3) ppm. **^{13}C-NMR** (100.63 MHz, C_6D_6): δ 190.9 (d), 152.7 (d), 149.2 (d), 143.3 (s), 138.8 (s), 134.0 (s), 120.6 (d), 116.7 (d), 67.5 (t), 26.1 (q, 3x), 18.5 (s), 14.6 (q), -5.23 (q, 2x) ppm. **IR** (NaCl): υ 2953 (m, C-H), 2887 (m, C-H), 2856 (m, C-H), 1706 (s, C=O), 1468 (w, C=C), 1254 (m, C-O), 1109 (m, Si-O-C) cm^{-1}. **HRMS** (ESI$^+$): calcd. for $C_{16}H_{26}NO_2Si$ ($[M+H]^+$), 292.1733; found, 292.1735.

Compounds 30 and 31.

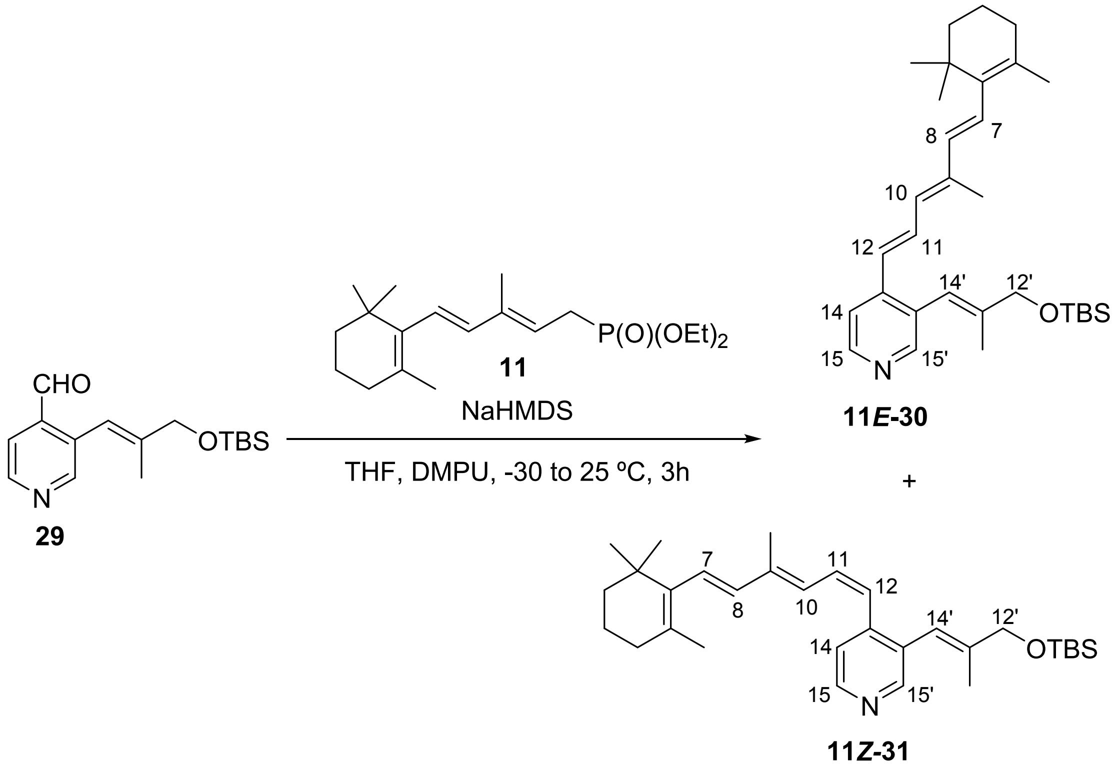

To a cooled (-30 °C) stirred solution of phosphonate **11** (0.17 g, 0.50 mmol) in THF (2 mL), DMPU (0.73 mL) and NaHMDS (0.47 mL, 1M in THF, 0.47 mmol) were added. Subsequently, **29** (85.0 mg, 0.29 mmol) in THF (2 mL) was added at -30 °C and the mixture was stirred for 3h at room temperature. A saturated aqueous solution of NH_4Cl was added and the mixture was extracted with Et_2O (3x). The combined organic layers were washed with brine, dried over Na_2SO_4, filtered and the solvent was evaporated. The residue was purified by flash column chromatography (silica gel, 98:2 *v/v n*-hexane/Et_3N; then, from 100:0 to 90:10 *v/v n*-hexane/EtOAc) to afford 62.1 mg (45% yield) of a yellow oil, which was identified as **30** and 61.8 mg (44% yield) of a yellow oil, which was identified as **31**.

Data for **30**: **^{1}H-NMR** (400.16 MHz, C_6D_6): δ 8.68 (s, 1H, $H_{15'}$), 8.53 (d, J = 5.0 Hz, 1H, H_{15}), 7.24 (dd, J = 15.4, 11.3 Hz, 1H, H_{11}), 7.12 (d, J = 5.0 Hz, 1H, H_{14}), 6.75 (d, J = 15.4 Hz, 1H, H_{12}), 6.65 (d, J = 1.6 Hz, 1H, $H_{14'}$), 6.36 (d, J = 16.0 Hz, 1H, H_7), 6.27 (d, J = 16.0 Hz, 1H, H_8), 6.22 (d, J = 11.3 Hz, 1H, H_{10}), 4.04 (d, J = 0.9 Hz, 2H, 2x$H_{12'}$), 1.96 (t, J = 6.3 Hz, 2H, CH_2), 1.89 (d, J = 1.1 Hz, 3H, CH_3), 1.77 (d, J = 1.0 Hz, 3H, CH_3), 1.63 – 1.56 (m, 2H, CH_2), 1.53 (d, J = 1.3 Hz, 3H, CH_3), 1.50 – 1.45 (m, 2H, CH_2), 1.12 (s, 6H, 2xCH_3), 0.98 (s, 9H,

$SiC(\underline{CH_3})_3$), 0.07 (s, 6H, 2xSi-CH_3) ppm. **^{13}C-NMR** (100.63 MHz, C_6D_6): δ 151.8 (d), 148.6 (d), 143.2 (s), 140.8 (s), 138.8 (s), 138.2 (s), 138.1 (d), 131.5 (s), 130.6 (d), 130.1 (d), 129.9 (s), 128.6 (d), 128.5 (d), 119.4 (d), 118.9 (d), 67.9 (t), 39.9 (t), 34.6 (s), 33.3 (t), 29.2 (q, 2x), 26.1 (q, 3x), 22.0 (q), 19.7 (t), 18.6 (s), 14.8 (q), 12.9 (q), -5.1 (q, 2x) ppm. **IR** (NaCl): υ 2954 (s, C-H), 2929 (s, C-H), 2857 (m, C-H), 1577 (m, C=C), 1254 (w, C-O), 1082 (m, Si-O-C) cm^{-1}. **HRMS** (ESI^+): calcd. for $C_{31}H_{48}NOSi$ ($[M+H]^+$), 478.3505; found, 478.3499.

Data for **31**: **^{1}H-NMR** (400.16 MHz, C_6D_6): δ 8.75 (s, 1H, $H_{15'}$), 8.46 (d, *J* = 5.0 Hz, 1H, H_{15}), 7.13 (d, *J* = 5.0 Hz, 1H, H_{14}), 6.64 (t, *J* = 11.6 Hz, 1H, H_{11}), 6.60 (s, 1H, $H_{14'}$), 6.51 (d, *J* = 12.0 Hz, 1H, H_{12}), 6.37 (d, *J* = 11.4 Hz, 1H, H_{10}), 6.30 (d, *J* = 16.0 Hz, 1H, H_7), 6.16 (d, *J* = 16.0 Hz, 1H, H_8), 4.02 (d, *J* = 0.9 Hz, 2H, 2x$H_{12'}$), 1.91 (t, *J* = 6.1 Hz, 2H, CH_2), 1.83 (d, *J* = 0.7 Hz, 3H, CH_3), 1.66 (d, *J* = 1.0 Hz, 3H, CH_3), 1.63 – 1.51 (m, 5H, CH_2 + CH_3), 1.48 – 1.42 (m, 2H, CH_2), 1.07 (s, 6H, 2xCH_3), 0.99 (s, 9H, $SiC(\underline{CH_3})_3$), 0.07 (s, 6H, 2xSi-CH_3) ppm. **^{13}C-NMR** (100.63 MHz, C_6D_6): δ 151.3 (d), 148.1 (d), 143.8 (s), 140.3 (s), 139.5 (s), 138.4 (d), 138.0 (s), 132.5 (s), 129.7 (s), 129.0 (d), 128.6 (d), 128.5 (d), 126.4 (d), 126.1 (d), 123.9 (d), 119.5 (d), 68.0 (t), 39.8 (t), 34.5 (s), 33.2 (t), 29.2 (q, 2x), 26.1 (q, 3x), 21.9 (q), 19.7 (t), 18.6 (s), 15.0 (q), 12.6 (q), -5.1 (q, 2x) ppm. **IR** (NaCl): υ 2953 (s, C-H), 2928 (s, C-H), 2857 (m, C-H), 1579 (m, C=C), 1253 (w, C-O), 1081 (m, Si-O-C) cm^{-1}. **HRMS** (ESI^+): calcd. for $C_{31}H_{48}NOSi$ ($[M+H]^+$), 478.3505; found, 478.3499.

((*E*)-2-(3-((*E*)-3-((*tert*-Butyldimethylsilyl)oxy)-2-methylprop-1-en-1-yl)pyridin-4-yl)vinyl)boronic Acid 32.

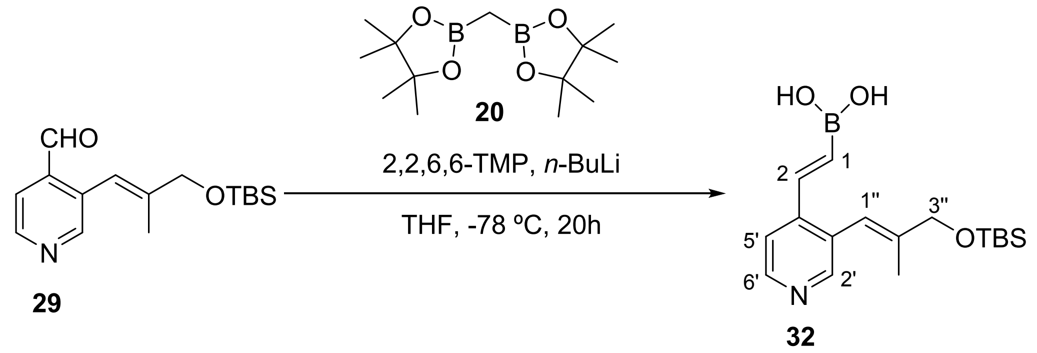

To a cooled (0 °C) solution of 2,2,6,6-tetramethylpiperidine (0.23 mL, 1.37 mmol) in THF (0.5 mL), *n*-BuLi (0.23 mL, 2.0M in hexanes, 1.37 mmol) was added and the mixture was stirred for 30 min. Then, a solution of bis(pinacolatoboryl)methane **20** (0.37 g, 1.37 mmol) in THF (1 mL) was added and the reaction mixture was stirred for 10 min. The

mixture was cooled down to -78 °C, a solution of **29** (0.1 g, 0.34 mmol) in THF (1 mL) was added dropwise, and the reaction mixture was stirred at -78 °C for 20h. A saturated aqueous solution of NH_4Cl was added and the mixture was extracted with Et_2O (3x). The combined organic layers were washed with brine, dried over anhydrous Na_2SO_4, filtered and the solvent was evaporated. The residue was purified by flash column chromatography (C18 silica gel, from 50:50 to 100:0 *v/v* CH_3OH/H_2O) to afford 73.2 mg (65% yield) of a red foam, which was identified as **32**. **^{1}H-NMR** (400.16 MHz, C_6D_6): δ 8.74 (s, 1H, $H_{2'}$), 8.56 (d, *J* = 5.4 Hz, 1H, $H_{6'}$), 7.79 (d, *J* = 17.9 Hz, 1H, H_2), 7.40 (d, *J* = 5.4 Hz, 1H, $H_{5'}$), 6.81 (d, *J* = 17.9 Hz, 1H, H_1), 6.77 (s, 1H, $H_{1''}$), 4.01 (s, 2H, 2x$H_{3''}$), 1.56 (s, 3H, CH_3), 0.94 (s, 9H, SiC($\underline{CH_3}$)$_3$), 0.07 (s, 6H, 2xSi-CH_3) ppm. **^{13}C-NMR** (100.63 MHz, C_6D_6): δ 149.5 (d), 146.1 (d), 146.0 (s), 141.9 (s), 139.6 (d) (from HSQC experiments), 137.1 (d) (from HSQC experiments), 132.5 (s), 120.4 (d), 118.1 (d), 67.7 (t), 26.2 (q, 3x), 18.5 (s), 15.0 (q), -5.1(q, 2x) ppm. **IR** (NaCl): υ 2953 (s, C-H), 2930 (s, C-H), 2856 (m, C-H), 1588 (m, C=C), 1255 (m, C-O), 1083 (m, Si-O-C) cm^{-1}. **HRMS** (ESI^+): calcd. for $C_{17}H_{29}BNO_3Si$ ($[M+H]^+$) 334. 2010; found, 334.2005.

Compound 30.

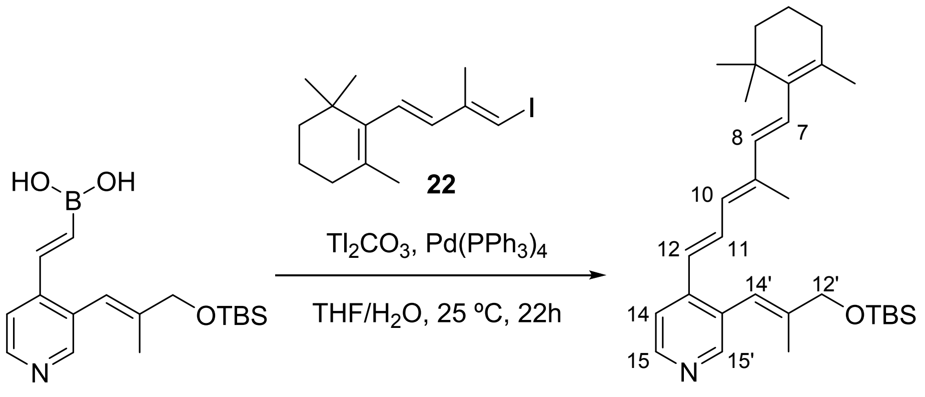

32

30

To a cooled (0 °C) solution of 2-((1*E*,3*E*)-4-iodo-3-methylbuta-1,3-dien-1-yl)-1,3,3-trimethylcyclohex-1-ene **22** (68.3 mg, 0.22 mmol) and **32** (60.0 mg, 0.18 mmol) in a THF-water mixture (6 mL, 4:1 v/v), Tl_2CO_3 (0.21 g, 0.45 mmol) and $Pd(PPh_3)_4$ (10.4 mg, 0.01 mmol) were added, and the reaction mixture was stirred at 25 °C for 22h. The mixture was filtered through Celite® and Na_2SO_4 washing with Et_2O and the solvent was evaporated. The residue was purified by flash column chromatography (silica gel, 98:2

v/v n-hexane/Et_3N; then, from 100:0 to 90:10 *v/v n*-hexane/EtOAc) to afford 67.1 mg (78% yield) of a dark yellow oil, which was identified as **30**.

Compound 33.

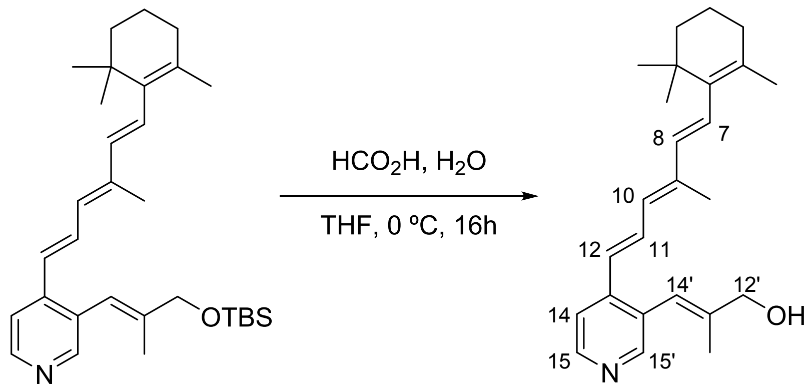

30 **33**

A cooled (0 °C) mixture of HCO_2H (1.6 mL, 43.30 mmol) and H_2O (0.5 mL, 29.66 mmol) was added to a cooled (0 °C) solution of **30** (60.0 mg, 0.13 mmol) in THF (3 mL). The mixture was stirred at 0 °C for 16h. A saturated aqueous solution of $NaHCO_3$ was then added and the mixture was extracted with EtOAc (3x). The combined organic layers were dried over Na_2SO_4, filtered and the solvent was evaporated. The residue was purified by flash column chromatography (silica gel, 98:2 *v/v n*-hexane/Et_3N; then, from 70:30 to 50:50 *v/v n*-hexane/EtOAc) to afford 36.3 mg (80% yield) of a dark orange oil, which was identified as **33**. **^{1}H-NMR** (400.16 MHz, C_6D_6): δ 8.62 (s, 1H, $H_{15'}$), 8.42 (d, *J* = 5.3 Hz, 1H, H_{15}), 7.23 (dd, *J* = 15.4, 11.4 Hz, 1H, H_{11}), 7.09 (d, *J* = 5.3 Hz, 1H, H_{14}), 6.70 (d, *J* = 15.4 Hz, 1H, H_{12}), 6.63 (s, 1H, $H_{14'}$), 6.36 (d, *J* = 16.1 Hz, 1H, H_7), 6.25 (d, *J* = 16.1 Hz, 1H, H_8), 6.14 (d, *J* = 11.4 Hz, 1H, H_{10}), 4.02 (s, 2H, 2x$H_{12'}$), 1.97 (t, *J* = 6.3 Hz, 2H, CH_2), 1.88 (d, *J* = 1.1 Hz, 3H, CH_3), 1.78 (s, 3H, CH_3), 1.64 – 1.56 (m, 2H, CH_2), 1.52 (d, *J* = 1.3 Hz, 3H, CH_3), 1.50 – 1.46 (m, 2H, CH_2), 1.13 (s, 6H, 2xCH_3) ppm. **^{13}C-NMR** (100.63 MHz, C_6D_6): δ 151.5 (d), 148.0 (d), 143.6 (s), 142.3 (s), 139.1 (s), 138.1 (s), 138.0 (d), 131.8 (s), 130.6 (d), 130.4 (d), 130.0 (s), 128.7 (d), 128.6 (d), 119.1 (d), 119.0 (d), 67.4 (t), 39.9 (t), 34.6 (s), 33.4 (t), 29.2 (q, 2x), 22.0 (q), 19.7 (t), 15.0 (q), 12.9 (q) ppm. **IR** (NaCl): υ 3500 – 3200 (br, O-H), 2954 (s, C-H), 2928 (s, C-H), 2858 (m, C-H), 1580 (s, C=C) cm^{-1}. **HRMS** (ESI$^+$): calcd. for $C_{25}H_{34}NO$ ([M+H]$^+$), 364.2640; found, 364.2635.

Compound 34.

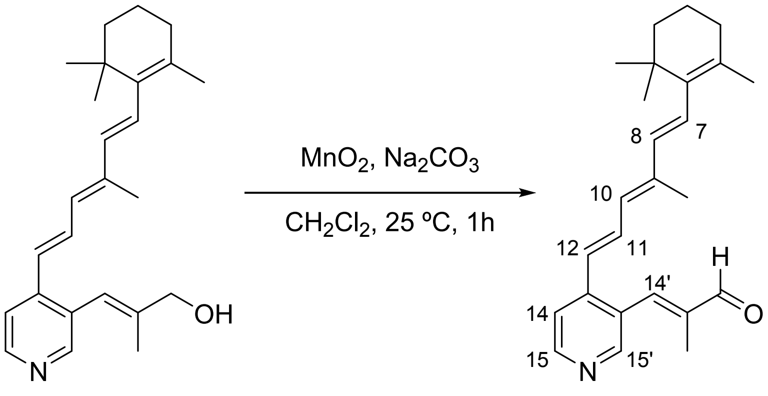

33 **34**

To a solution of **33** (36 mg, 0.10 mmol) in CH_2Cl_2 (3 mL) at room temperature, Na_2CO_3 (0.190 g, 1.80 mmol) and MnO_2 (0.156 g, 1.80 mmol) were added. The resulting reaction mixture was stirred at room temperature for 1h. The mixture was filtered through Celite® washing with CH_2Cl_2 to afford 26 mg (72% yield) of a dark red oil, which was identified as **34**. **^{1}H-NMR** (400.16 MHz, C_6D_6): δ 9.37 (s, 1H, CHO), 8.51 – 8.44 (m, 2H, $H_{15'}$ + H_{15}), 7.16 (dd, *J* = 15.2, 11.3 Hz, 1H, H_{11}), 7.01 (d, *J* = 5.3 Hz, 1H, H_{14}), 6.82 (s, 1H, $H_{14'}$), 6.41 (d, *J* = 16.1 Hz, 1H, H_7), 6.35 – 6.28 (m, 2H, H_{12} + H_8), 6.16 (d, *J* = 11.3 Hz, 1H, H_{10}), 1.96 (t, *J* = 6.4 Hz, 2H, CH_2), 1.87 (d, *J* = 1.1 Hz, 3H, CH_3), 1.78 (d, *J* = 0.9 Hz, 3H, CH_3), 1.70 (d, *J* = 1.4 Hz, 3H, CH_3), 1.63 – 1.54 (m, 2H, CH_2), 1.51 – 1.45 (m, 2H, CH_2), 1.13 (s, 6H, 2xCH_3) ppm. **^{13}C-NMR** (100.63 MHz, C_6D_6): δ 193.6 (d), 150.7 (d), 150.1 (d), 143.7 (d), 143.2 (s), 141.7 (s), 140.2 (s), 138.0 (s), 137.7 (d), 131.6 (d), 130.4 (s), 130.0 (d), 129.5 (d), 128.5 (s), 126.6 (d), 119.1 (d), 39.9 (t), 34.6 (s), 33.4 (t), 29.2 (q, 2x), 22.0 (q), 19.6 (t), 12.9 (q), 10.9 (q) ppm. **IR** (NaCl): υ 2954 (s, C-H), 2925 (s, C-H), 2857 (m, C-H), 1687 (s, C=O), 1576 (m, C=C) cm^{-1}. **HRMS** (ESI^+): calcd. for $C_{25}H_{32}NO$ ($[M+H]^+$) 362.2484; found, 362.2478.

Compound 35.

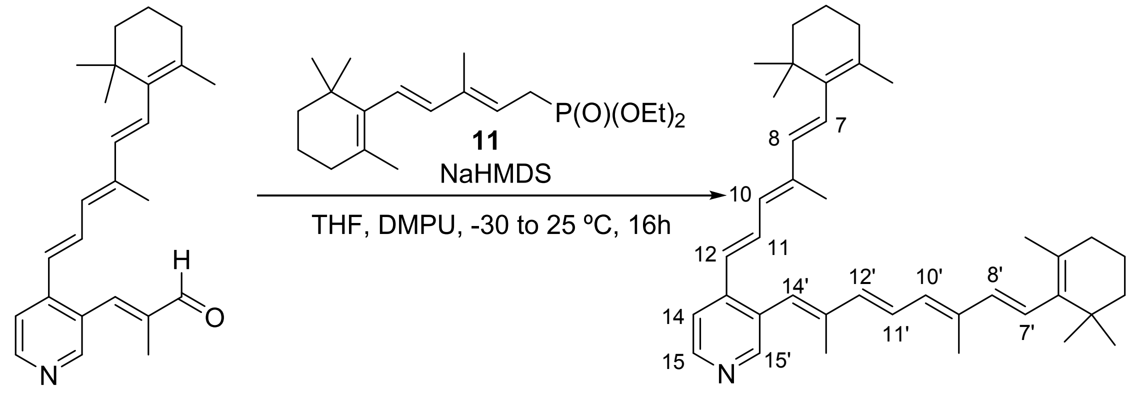

34 **35**

To a cooled (-30 °C) stirred solution of phosphonate **11** (41.6 mg, 0.12 mmol) in THF (0.4 mL), DMPU (0.2 mL) and NaHMDS (0.12 mL, 1M in THF, 0.12 mmol) were added. Subsequently, **34** (26.0 mg, 0.07 mmol) in THF (0.4 mL) was added and the mixture was allowed to warm up to room temperature for 16h. A saturated aqueous solution of NH_4Cl was added and the mixture was extracted with Et_2O (3x). The combined organic layers were washed with brine, dried over Na_2SO_4, filtered and the solvent was evaporated. The residue was purified by flash column chromatography (silica gel, 98:2 *v/v* *n*-hexane/Et_3N; then, from 100:0 to 90:10 *v/v* *n*-hexane/EtOAc) to afford 37.7 mg (96% yield) of a dark yellow oil, which was identified as **35**. **^{1}H-NMR** (400.16 MHz, C_6D_6): δ 8.67 (s, 1H, $H_{15'}$), 8.51 (d, J = 5.3 Hz, 1H, H_{15}), 7.24 (dd, J = 15.3, 11.4 Hz, 1H, H_{11}), 7.10 (d, J = 5.3 Hz, 1H, H_{14}), 6.75 (dd, J = 15.1, 11.2 Hz, 1H, $H_{11'}$), 6.63 (d, J = 15.3 Hz, 1H, H_{12}), 6.51 – 6.43 (m, 2H, $H_{12'}$ + $H_{14'}$), 6.41 – 6.25 (m, 5H, H_7 + $H_{7'}$ + H_8 + $H_{8'}$ + $H_{10'}$), 6.17 (d, J = 11.4 Hz, 1H, H_{10}), 2.01 – 1.93 (m, 4H, 2xCH_2), 1.90 (d, J = 1.1 Hz, 3H, CH_3), 1.88 (d, J = 1.1 Hz, 3H, CH_3), 1.80 (d, J = 0.9 Hz, 3H, CH_3), 1.78 (d, J = 1.1 Hz, 3H, CH_3), 1.77 (d, J = 0.9 Hz, 3H, CH_3), 1.64 – 1.54 (m, 4H, 2xCH_2), 1.49 (m, 4H, 2xCH_2), 1.14 (s, 6H, 2xCH_3), 1.13 (s, 6H, 2xCH_3) ppm. **^{13}C-NMR** (100.63 MHz, C_6D_6): δ 151.9 (d), 148.6 (d), 143.2 (s), 139.3 (s), 139.1 (s), 138.6 (d), 138.3 (s), 138.1 (s), 138.0 (d), 137.2 (d), 136.7 (s), 131.1 (d), 130.6 (d), 130.4 (d), 130.0 (s), 129.4 (s), 128.7 (d), 128.6 (d), 127.9 (s), 127.3 (d), 126.8 (d), 126.4 (d), 119.1 (d), 39.9 (t, 2x), 34.6 (s), 34.5 (s), 33.3 (t, 2x), 29.2 (q, 4x), 22.0 (q), 21.9 (q), 19.7 (t), 19.6 (t), 14.1 (q), 12.9 (q), 12.8 (q) ppm. **IR** (NaCl): υ 2954 (m, C-H), 2924 (s, C-H), 2856 (m, C-H), 1455 (m, C=C) cm^{-1}. **HRMS** (ESI^+): calcd. for $C_{40}H_{54}N$ ($[M+H]^+$) 548.4256; found, 548.4250. **UV** (CH_3OH): λ_{max} 338 nm (ε = 27,100).

***i*-A2E (9).**

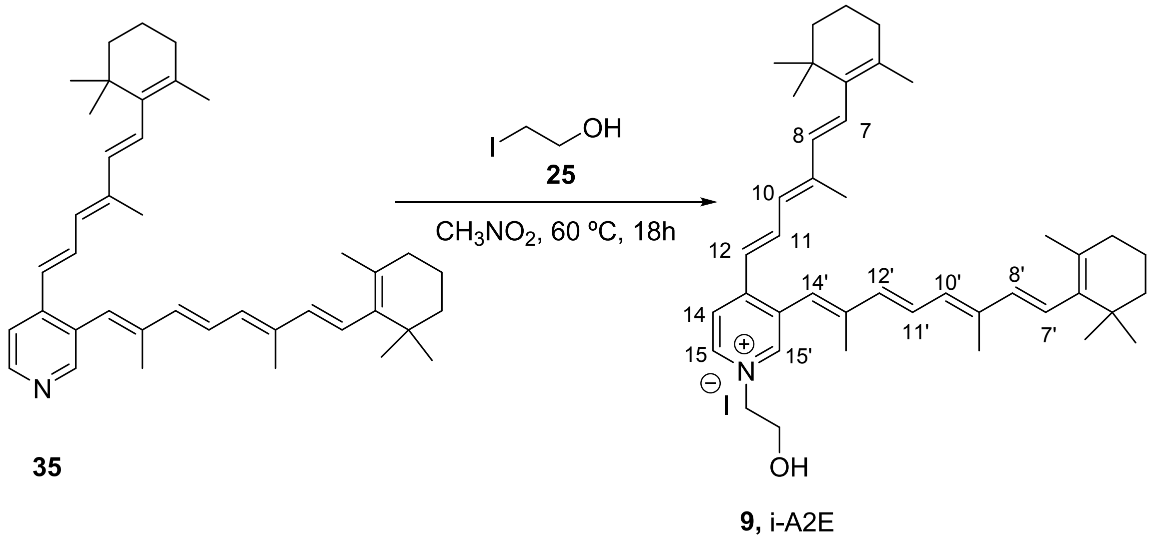

To a solution of **35** (8.0 mg, 0.02 mmol) in CH_3NO_2 (0.2 mL), 2-iodoethanol **25** (0.01 mL, 0.15 mmol) was added, and the resulting solution was heated at 60 °C for 18h. The solvent was evaporated under vacuum, the residue was triturated with *n*-hexane and Et_2O mixtures and the solvents were removed to afford 7.6 mg (72% yield) of a dark red solid, which was identified as *i*-A2E (**9**). **^{1}H-NMR** (400.16 MHz, CD_3OD): δ 8.55 (s, 1H, $H_{15'}$), 8.51 (d, J = 6.7 Hz, 1H, H_{15}), 8.30 (d, J = 6.7 Hz, 1H, H_{14}), 7.99 (dd, J = 15.1, 11.5 Hz, 1H, H_{11}), 6.96 (dd, J = 15.2, 11.3 Hz, 1H, $H_{11'}$), 6.78 (d, J = 15.1 Hz, 1H, H_{12}), 6.62 (d, J = 15.2 Hz, 1H, $H_{12'}$), 6.58 – 6.53 (m, 2H, H_7 + $H_{14'}$), 6.45 (d, J = 11.5 Hz, 1H, H_{10}), 6.32 – 6.26 (m, 2H, H_8 + $H_{7'}$), 6.23 (d, J = 11.3 Hz, 1H, $H_{10'}$), 6.16 (d, J = 16.1 Hz, 1H, $H_{8'}$), 4.57 (t, J = 4.9 Hz, 2H, CH_2), 3.99 (t, J = 4.9 Hz, 2H, CH_2), 2.18 (d, J = 1.1 Hz, 3H, CH_3), 2.08 – 2.03 (m, 4H, 2xCH_2), 2.01 (d, J = 1.1 Hz, 3H, CH_3), 2.00 (d, J = 1.2 Hz, 3H, CH_3), 1.74 (s, 3H, CH_3), 1.72 (s, 3H, CH_3), 1.69 – 1.62 (m, 4H, 2xCH_2), 1.56 – 1.44 (m, 4H, 2xCH_2), 1.06 (s, 6H, 2xCH_3), 1.05 (s, 6H, 2xCH_3) ppm. **^{13}C-NMR** (100.63 MHz, CD_3OD): δ 153.5 (s), 147.1 (s), 145.9 (d), 144.1 (s), 142.0 (d), 139.9 (d), 139.2 (s), 139.1 (s), 139.0 (d), 138.9 (s), 138.4 (d), 136.4 (d), 135.3 (s), 133.1 (d), 132.3 (s), 131.1 (d), 130.9 (d), 130.5 (s), 129.6 (d), 128.9 (d), 124.7 (d), 122.9 (d), 122.4 (d), 63.6 (t), 61.8 (t), 40.8 (t, 2x), 35.3 (s), 35.2 (s), 34.1 (t), 34.0 (t), 29.4 (q, 4x), 22.0 (q), 21.9 (q), 20.3 (t), 20.2 (t), 14.3 (q), 13.2 (q), 12.8 (q) ppm. **HRMS** (ESI$^+$): calcd. for $C_{42}H_{58}NO^+$ ([M$^+$]) 592.4513; found, 592.4512. **UV** (CH_3OH): λ_{max} 348 nm (ε = 27,300), 432 nm (ε = 23,800). **FES** (430 nm) = λ_{max} 580 nm.

3. **Comparison of the ^{1}H-NMR spectra of *iiso*-A2E (8) (natural),[4] *iiso*-A2E (8) (synthetic) and *i*-A2E 9.**

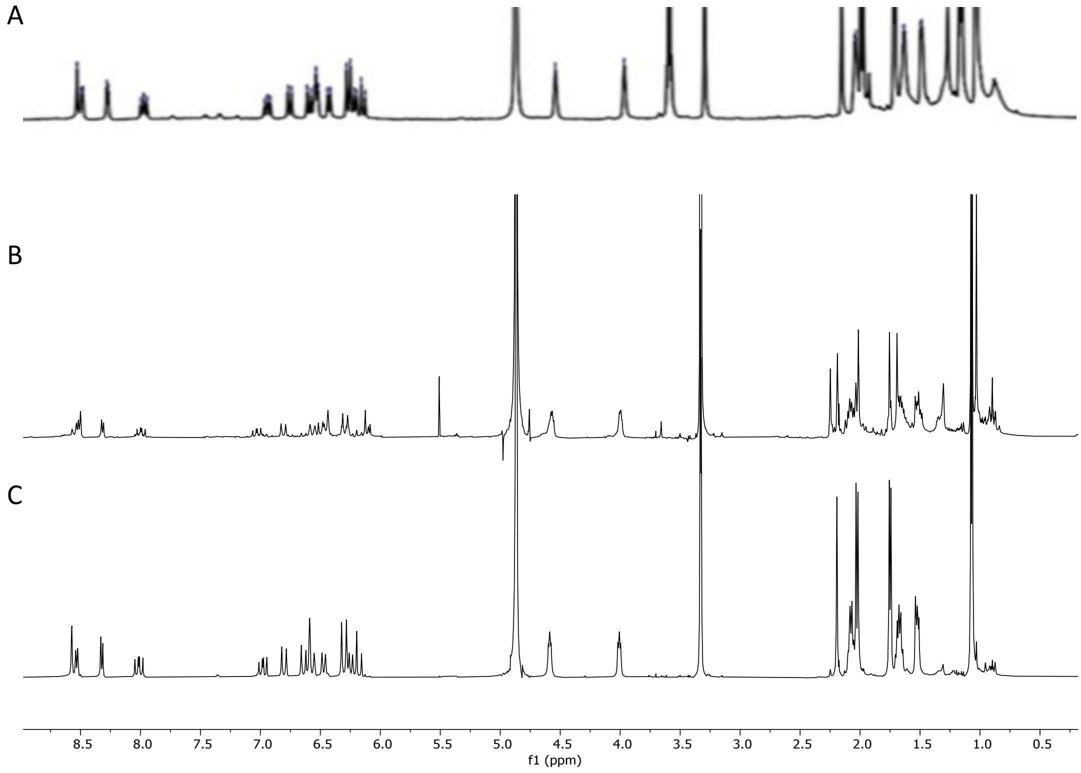

Figure S1. (**A**) ^{1}H-NMR (500 MHz, CD_3OD) of *iiso*-A2E (**8**) (natural).[4] (**B**) ^{1}H-NMR (400 MHz, CD_3OD) of *iiso*-A2E (**8**) (synthetic). (**C**) ^{1}H-NMR (400 MHz, CD_3OD) of *i*-A2E (**9**).Haga clic o pulse aquí para escribir texto.

Table S1. Comparison of the ^{1}H-NMR spectra (500 MHz, CD_3OD for **8** and 400.16 MHz, CD_3OD for **8** and **9**) of *iiso*-A2E (**8**) (natural)[4], *iiso*-A2E (**8**) (synthetic), and *i*-A2E (**9**).

	δ **8** (natural)	δ **8** (synthetic)	δ **9** (synthetic)
1-$(CH_3)_2$	1.05 (s)	1.06 (s)	1.06 (s)
1’-$(CH_3)_2$	1.04 (s)	1.01 (s)	1.05 (s)
2	1.51 (m)	1.59 – 1.44 (m)	1.56 – 1.44 (m)
2’	1.51 (m)	1.34 – 1.27 (m)	1.56 – 1.44 (m)
3	1.66 (m)	1.66 – 1.57 (m)	1.69 – 1.62 (m)
3’	1.66 (m)	1.66 – 1.57 (m)	1.69 – 1.62 (m)
4	2.06 (m)	2.13 – 2.02 (m)	2.08 – 2.03 (m)
4’	2.06 (m)	2.13 – 2.02 (m)	2.08 – 2.03 (m)
5-CH_3	1.73 (s)	1.74 (d, *J* = 0.9 Hz)	1.74 (s)
5’-CH_3	1.72 (s)	1.67 (s)	1.72 (s)
6	-	-	-
6’	-	-	-
7	6.56 (d, *J* = 15.5 Hz)	6.55 (d, *J* = 16.1 Hz)	6.58 – 6.53 (m)
7’	6.26 (d, *J* = 16.1 Hz)	6.13 – 6.06 (m)	6.32 – 6.26 (m)
8	6.29 (d, *J* = 16.1 Hz)	6.34 – 6.25 (m)	6.32 – 6.26 (m)
8’	6.17 (d, *J* = 16.0 Hz)	6.34 – 6.25 (m)	6.16 (d, *J* = 16.1 Hz)
9-CH_3	2.17 (s)	2.17 (s)	2.18 (d, *J* = 1.1 Hz)
9’-CH_3	1.99 (s)	2.00 (s)	2.00 (d, *J* = 1.2 Hz)
10	6.45 (d, *J* = 11.4 Hz)	6.45 – 6.41 (m)	6.45 (d, *J* = 11.5 Hz)
10’	6.23 (d, *J* = 11.4 Hz)	6.13 – 6.06 (m)	6.23 (d, *J* = 11.3 Hz)
11	8.00 (dd, *J* = 11.8, 14.9 Hz)	7.97 (dd, *J* = 15.1, 11.6 Hz)	7.99 (dd, *J* = 15.1, 11.5 Hz)
11’	6.98 (dd, *J* = 11.5, 14.9 Hz)	7.01 (dd, *J* = 15.0, 11.4 Hz)	6.96 (dd, *J* = 15.2, 11.3 Hz)
12	6.78 (d, *J* = 15.0 Hz)	6.79 (d, *J* = 15.1 Hz)	6.78 (d, *J* = 15.1 Hz)
12’	6.62 (d, *J* = 15.1 Hz)	6.48 (d, *J* = 15.1 Hz	6.62 (d, *J* = 15.2 Hz)
13	-	-	-
13’-CH_3	2.01 (s)	2.23 (d, *J* = 1.4 Hz)	2.01 (d, *J* = 1.1 Hz)
14	8.29 (d, *J* = 6.6 Hz)	8.30 (d, *J* = 6.8 Hz)	8.30 (d, *J* = 6.7 Hz)
14’	6.55 (s)	6.45 – 6.41 (m)	6.58 – 6.53 (m)
15	8.50 (d, *J* = 6.4 Hz)	8.51 (d, *J* = 6.8 Hz)	8.51 (d, *J* = 6.7 Hz)
15’	8.54 (s)	8.48 (s)	8.55 (s)
20	-	-	-
N-CH_2	4.55 (t)	4.55 (t, *J* = 5.4 Hz)	4.57 (t, *J* = 4.9 Hz)
CH_2-O	3.98 (t)	3.98 (t, *J* = 5.4 Hz)	3.99 (t, *J* = 4.9 Hz)

Table S2. Comparison of the ^{13}C-NMR spectra (125 MHz, CD_3OD for **8** and 100.63 MHz, CD_3OD for **8** and **9**) of *iiso*-A2E (**8**) (natural),[4] *iiso*-A2E (**8**) (synthetic), and *i*-A2E (**9**).

	δ **8** (natural)	δ **8** (synthetic)	δ **9** (synthetic)
1		35.3	35.3
1′		35.2	35.2
1-$(CH_3)_2$	29.4	29.4	29.4
1′-$(CH_3)_2$	29.4	29.4	29.4
2	34.1	34.1	34.1
2′	34.0	34.0	34.0
3	20.3	20.3	20.3
3′	20.2	20.2	20.2
4	40.8	40.8	40.8
4′	40.8	40.7	40.8
5		139.1	139.2
5′		130.6	132.3
5-CH_3	22.0	22.0	22.0
5′-CH_3	21.9	21.9	21.9
6		147.0	147.1
6′		132.3	130.5
7	133.1	133.1	133.1
7′	128.9	139.0	128.9
8	138.4	138.4	134.4
8′	139.1	129.2	139.1
9		138.9	139.1
9′		139.9	138.9
9-CH_3	13.2	13.2	13.2
9′-CH_3	14.3	12.8	14.3
10	130.9	130.9	130.9
10′	131.1	131.2	131.1
11	139.8	139.8	139.9
11′	129.6	131.9	129.6
12	124.8	124.9	124.7
12′	136.4	129.1	136.4
13		153.6	153.5
13′		143.1	144.1
13′-CH_3	12.8	20.8	12.8
14	122.9	123.0	122.9

14’	122.4	120.5	122.4
15	142.0	141.8	142.0
15’	145.9	146.7	145.9
20		134.9	135.3
N-CH_2	61.8	63.6	61.8
CH_2-O	65.6	61.8	63.6

4. UV spectra

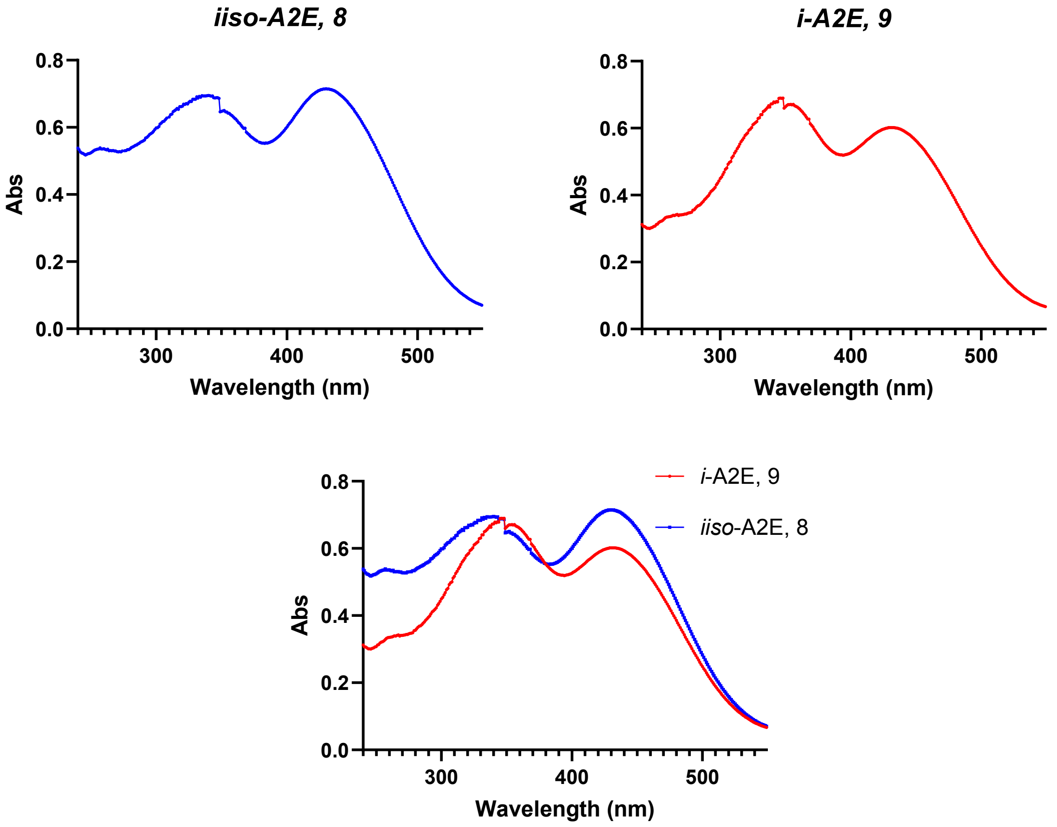

Figure S2. Comparison of UV-Vis spectra of *iiso*-A2E (**8**) (synthetic), and *i*-A2E (**9**).

5. References

(1) Duboudin, J. G.; Jousseaume, B.; Bonakdar, A.; Saux, A. *J. Organomet. Chem.* **1979**, *168*, 227–232.

(2) Bulger, P. G.; Moloney, M. G.; Trippier, P. C. *Org. Biomol. Chem* **2003**, *1*, 3726–3737.

(3) Vaz, B.; Fontán, N.; Castiñeira, M.; Álvarez, R.; de Lera, Á. R. *Org. Biomol. Chem.* **2015**, *13*, 3024–3031.

(4) Li, J.; Yao, K.; Yu, X.; Dong, X.; Gan, L.; Luo, C.; Wu, Y. *J. Biol. Chem.* **2013**, *288*, 35671–35682.

6. Copies of NMR spectra

^{1}H-NMR (400.16 MHz, CD_3Cl_3)

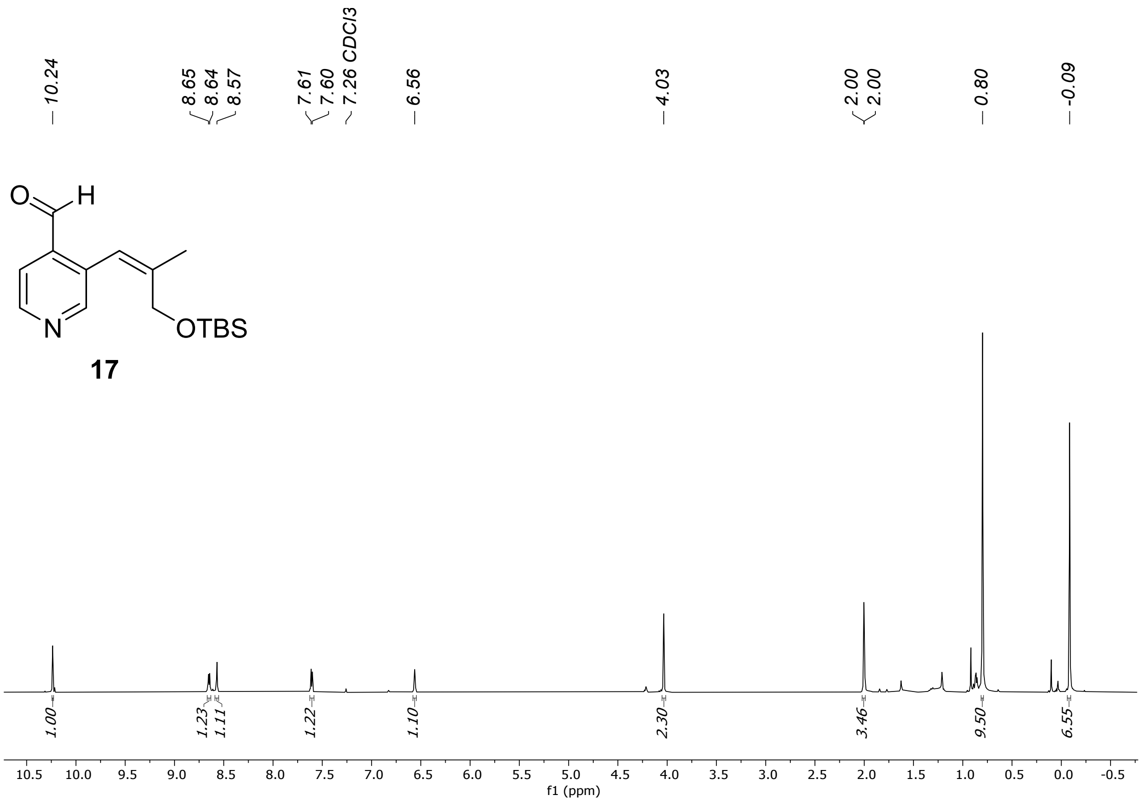

^{13}C-NMR (100.63 MHz, CD_3Cl_3)

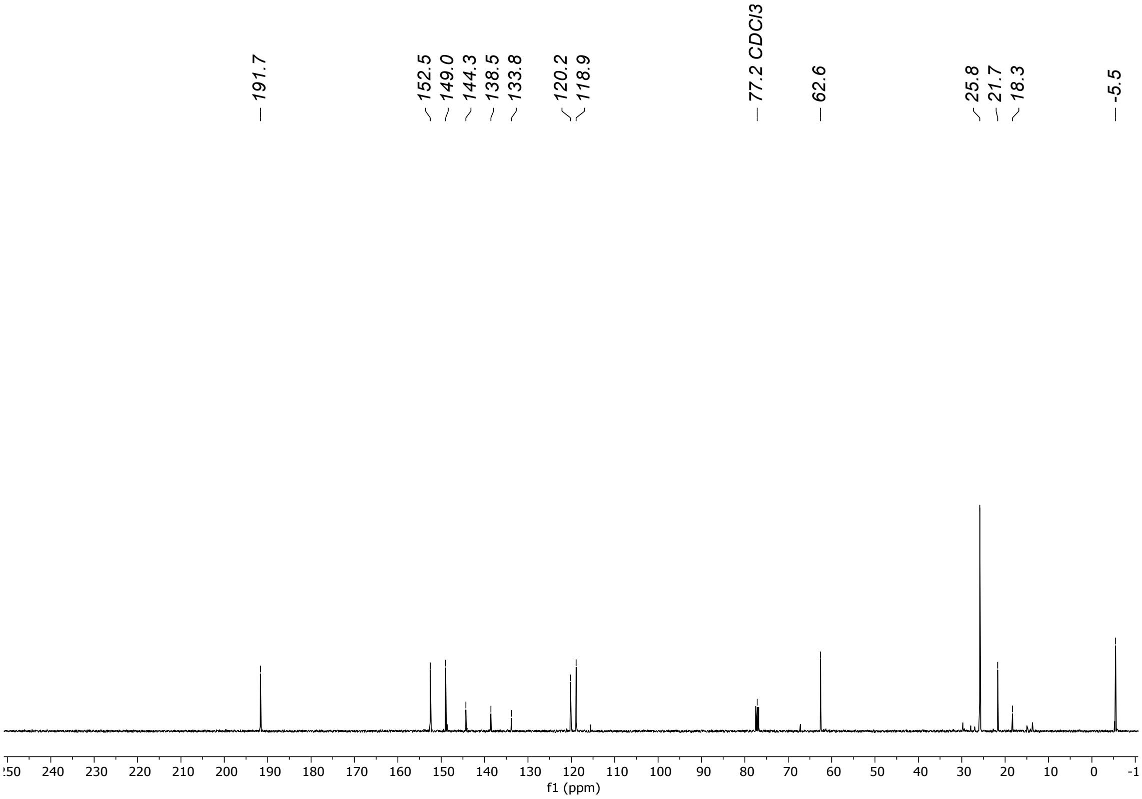

^{1}H-NMR (400.16 MHz, C_6D_6)

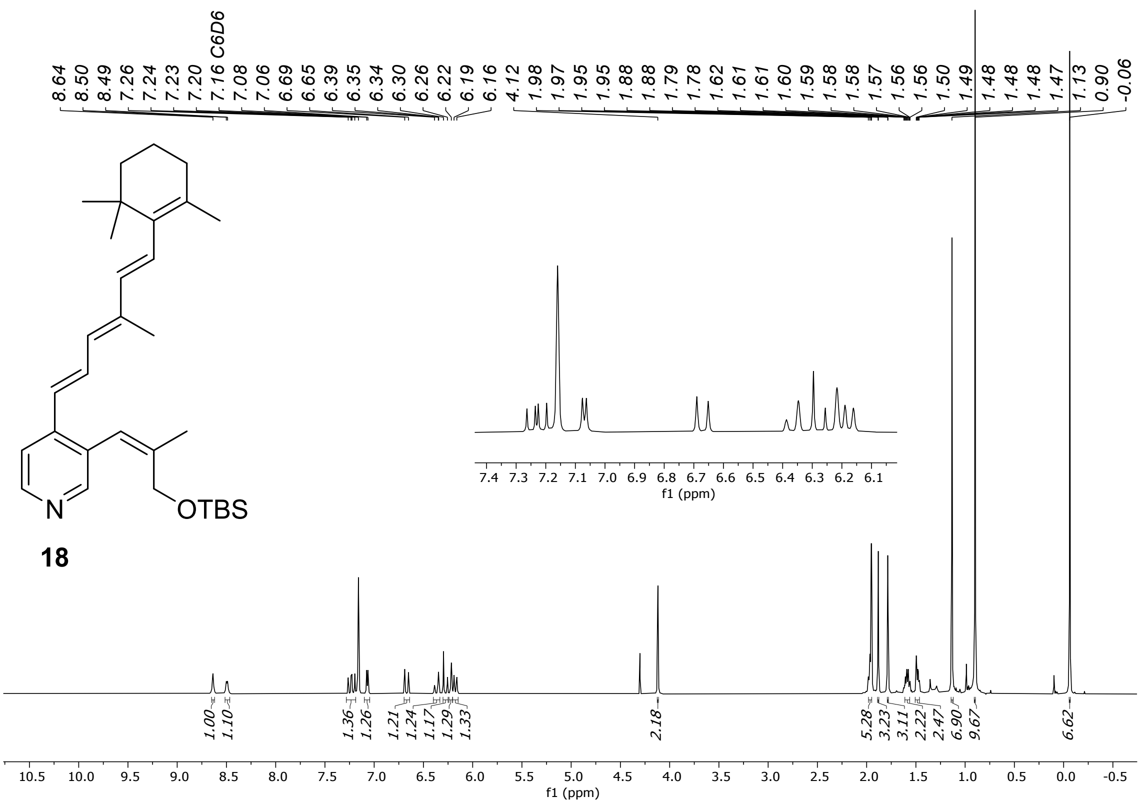

18

^{13}C-NMR (100.63 MHz, C_6D_6)

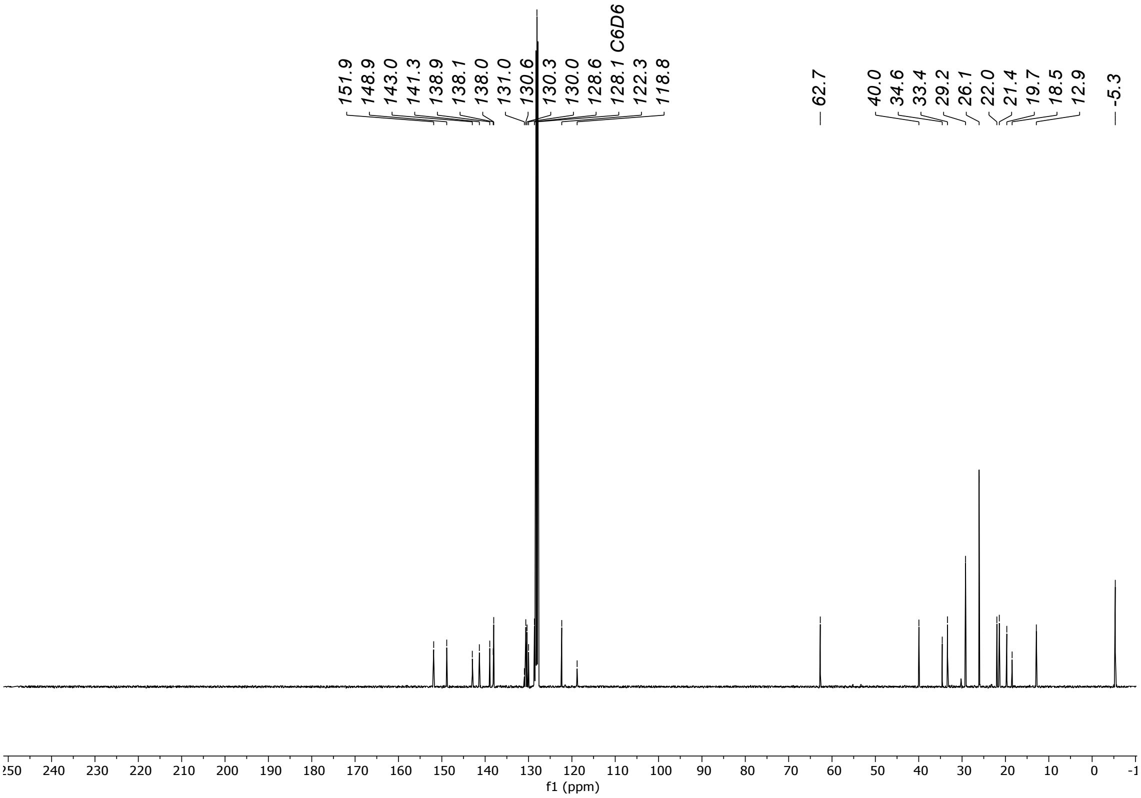

COSY (C_6D_6)

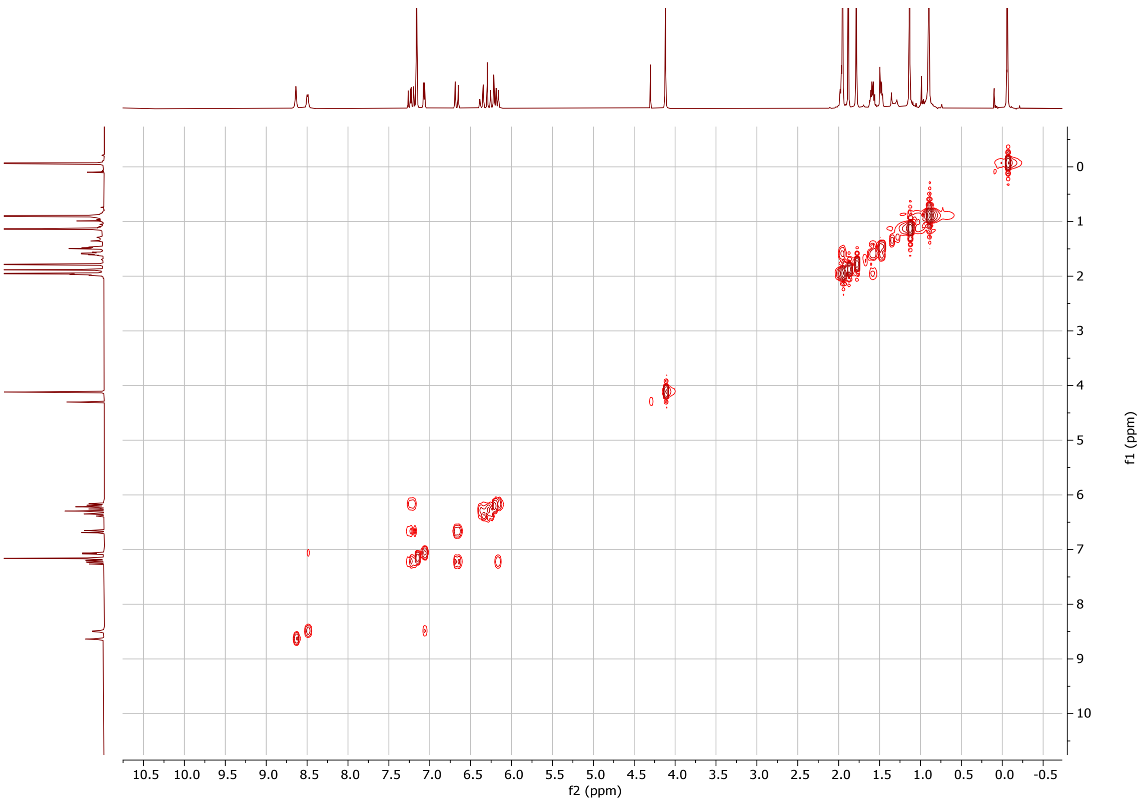

HSQC (C_6D_6)

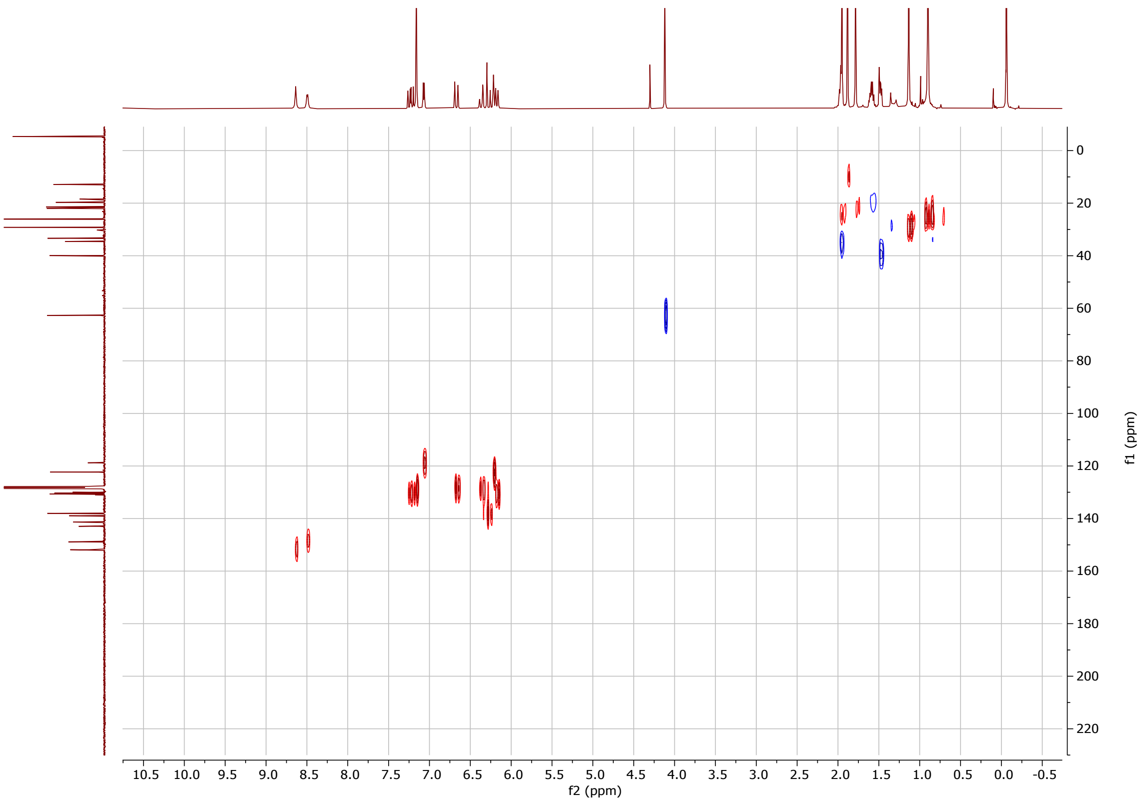

HMBC (C_6D_6)

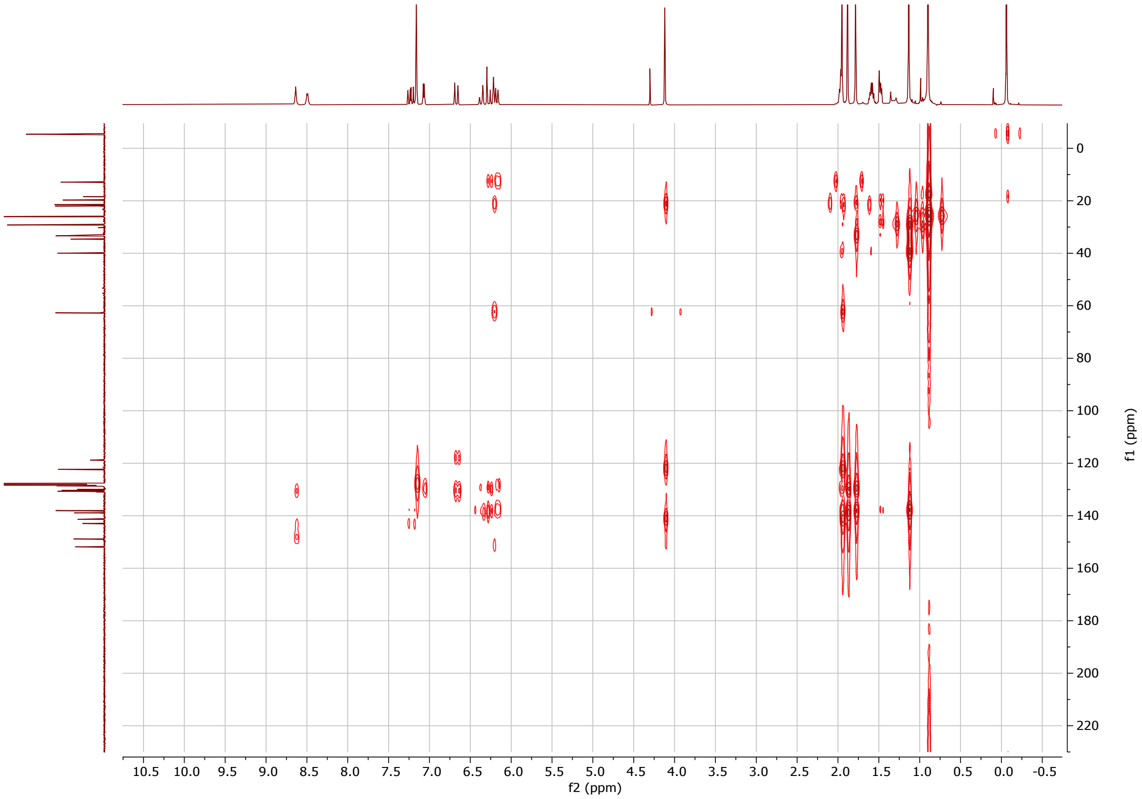

^{1}H-NMR (400.16 MHz, C_6D_6)

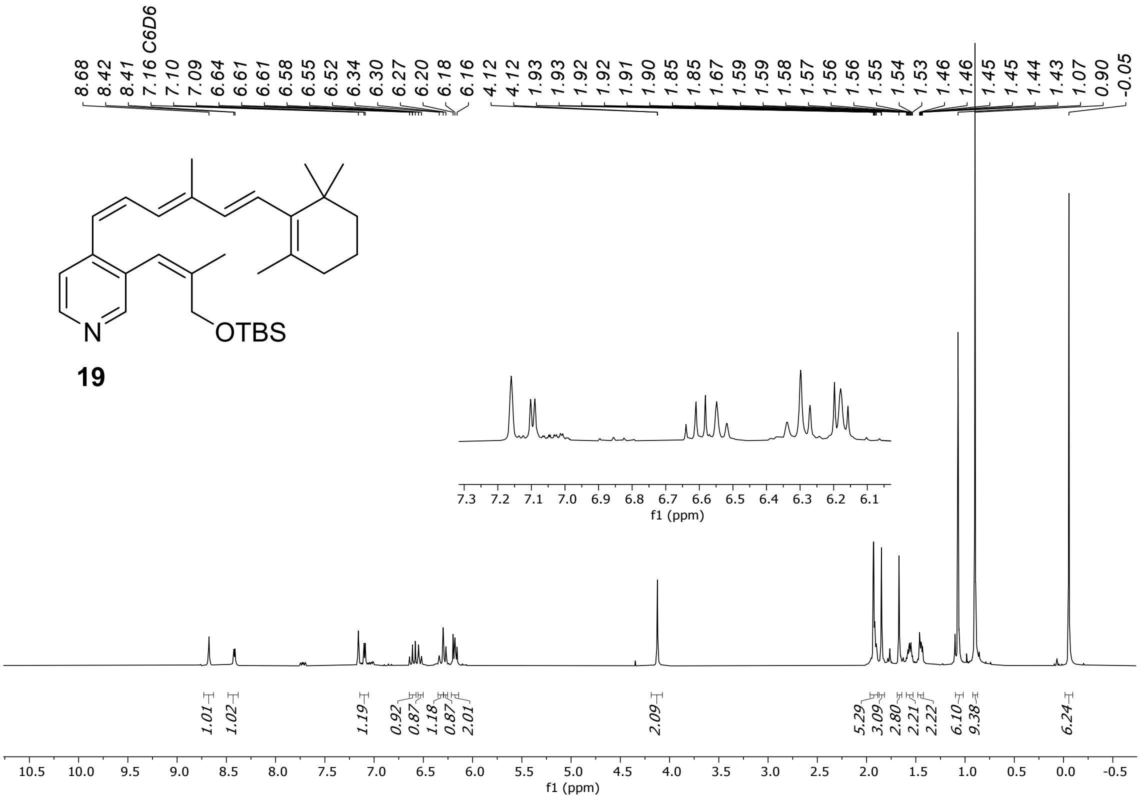

^{13}C-NMR (100.63 MHz, C_6D_6)

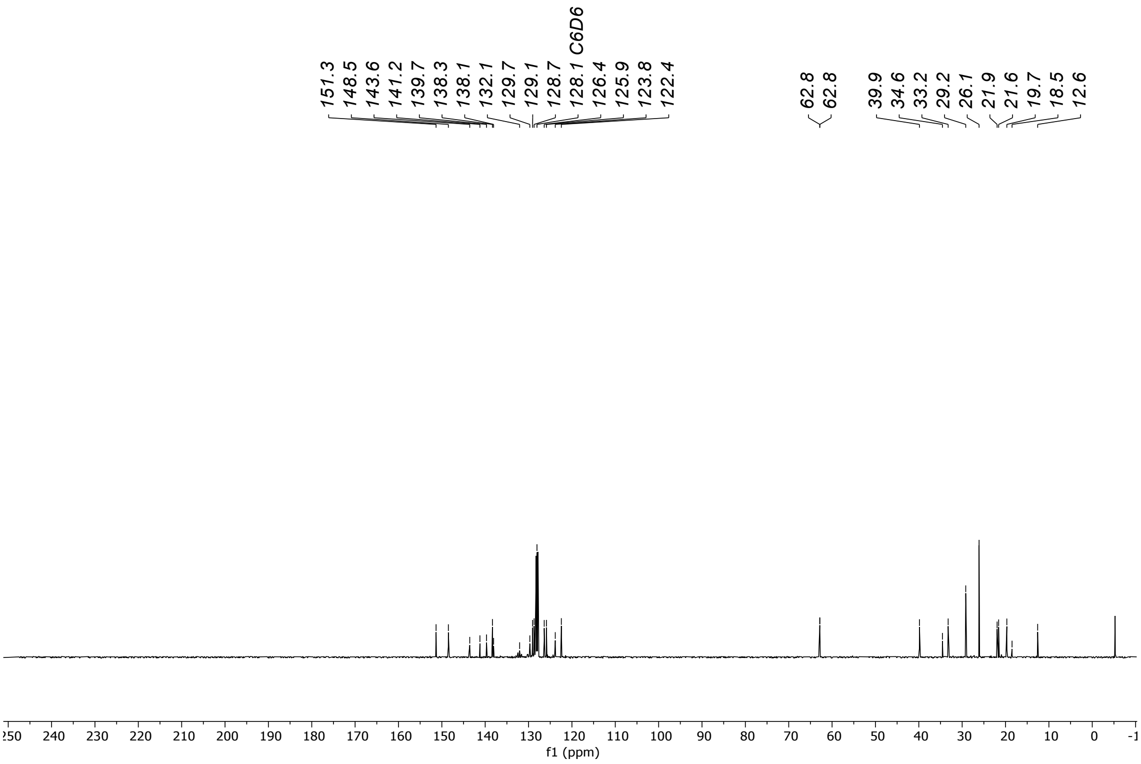

^{1}H-NMR (400.16 MHz, C_6D_6)

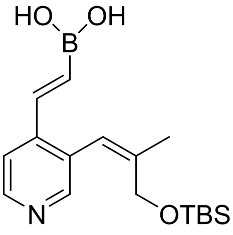

21

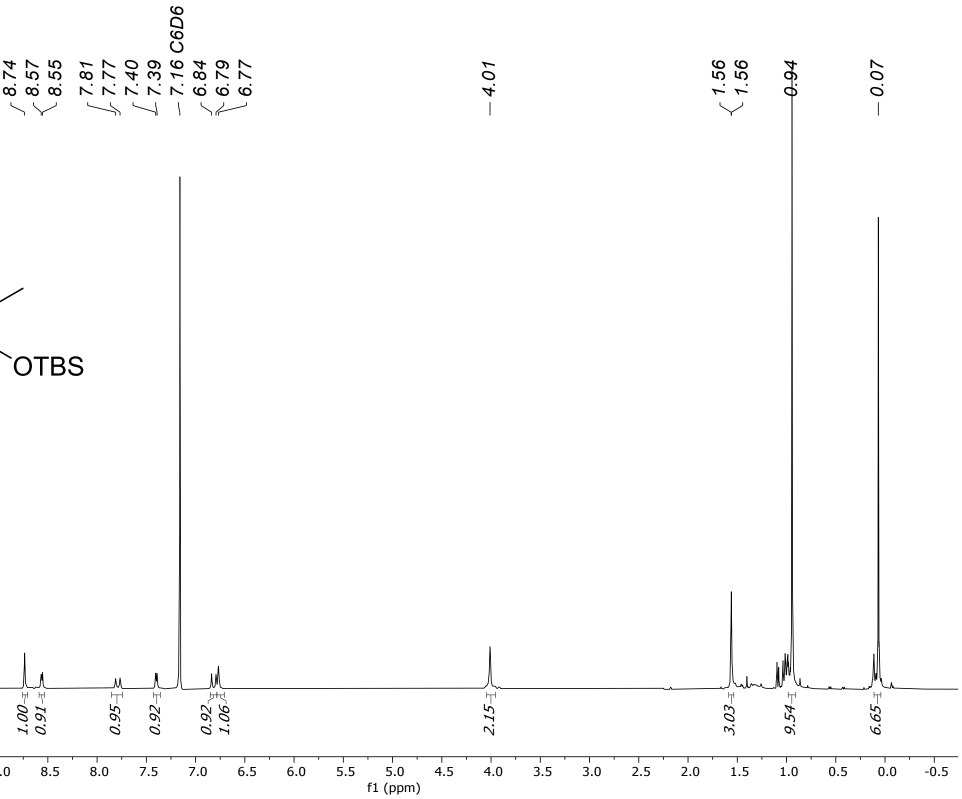

^{13}C-NMR (100.63 MHz, C_6D_6)

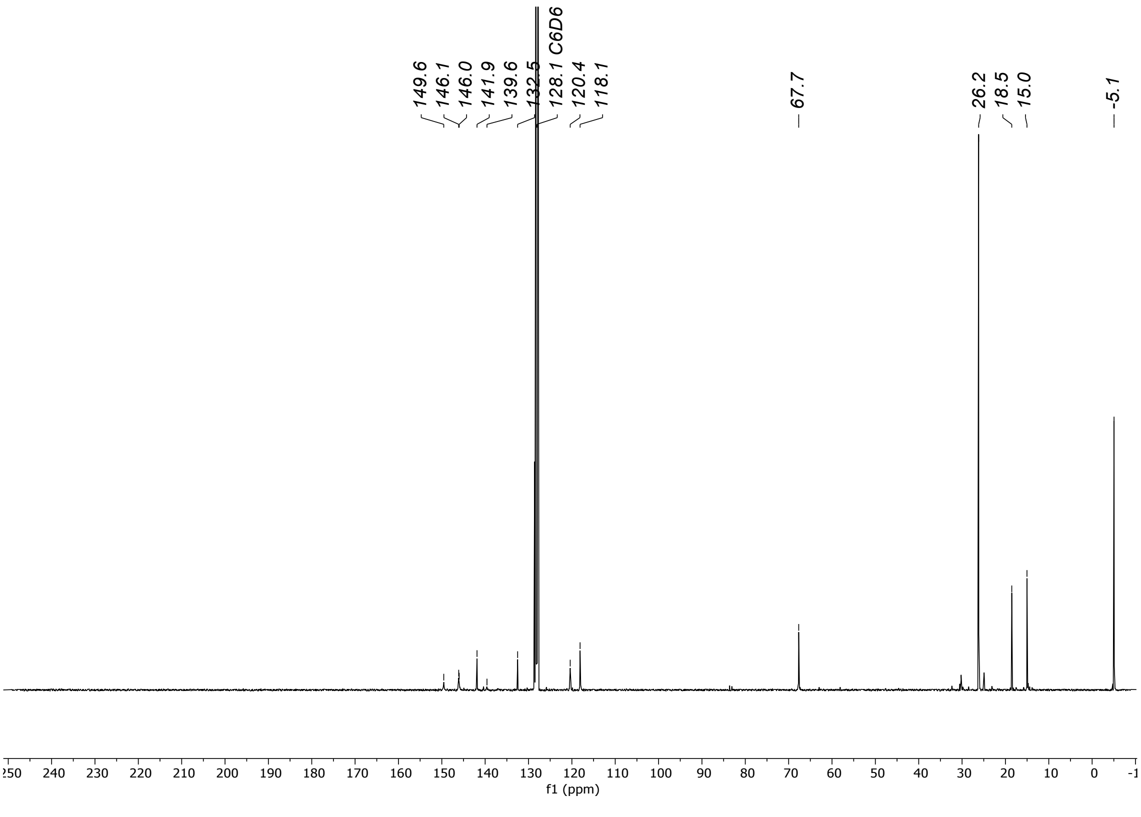

COSY (C_6D_6)

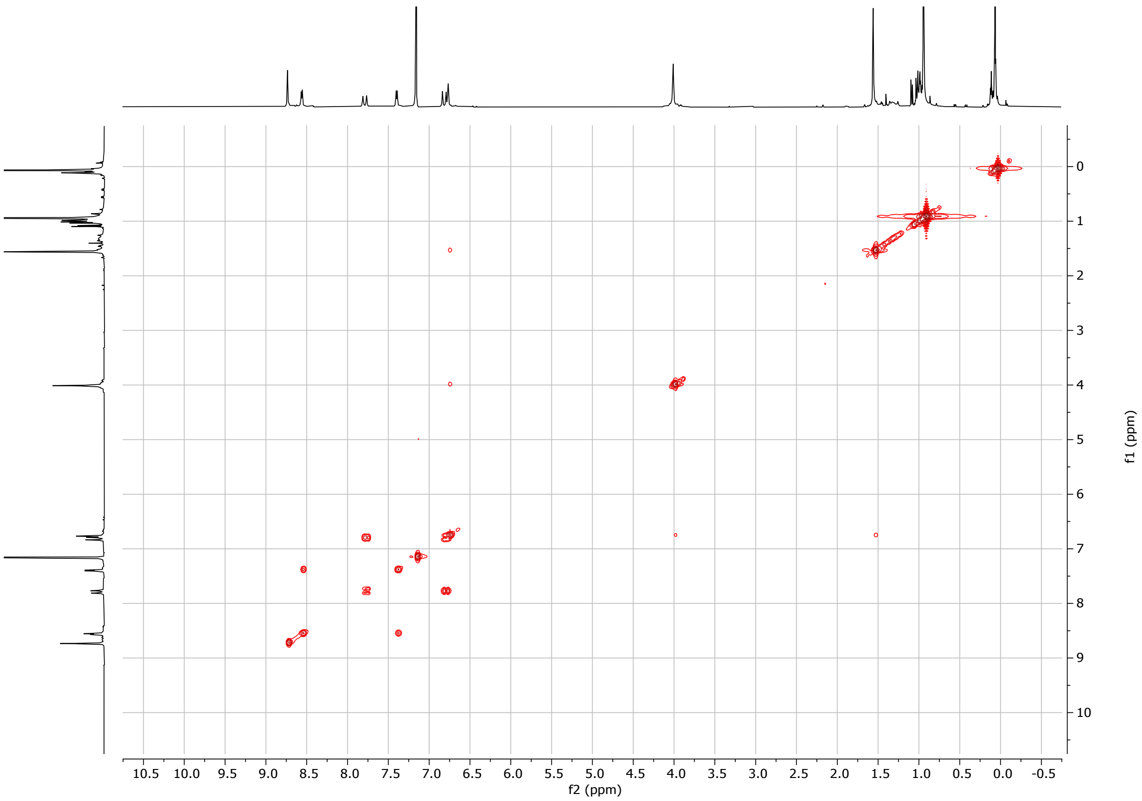

HSQC (C_6D_6)

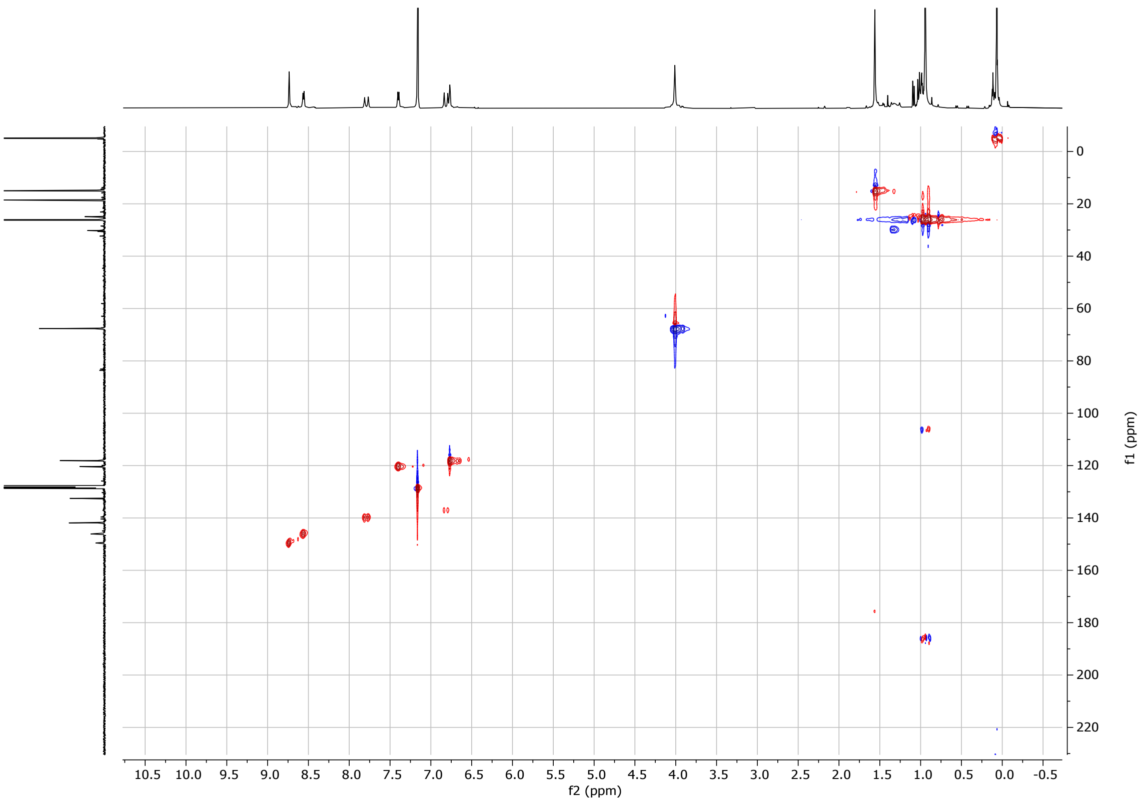

HMBC(C_6D_6)

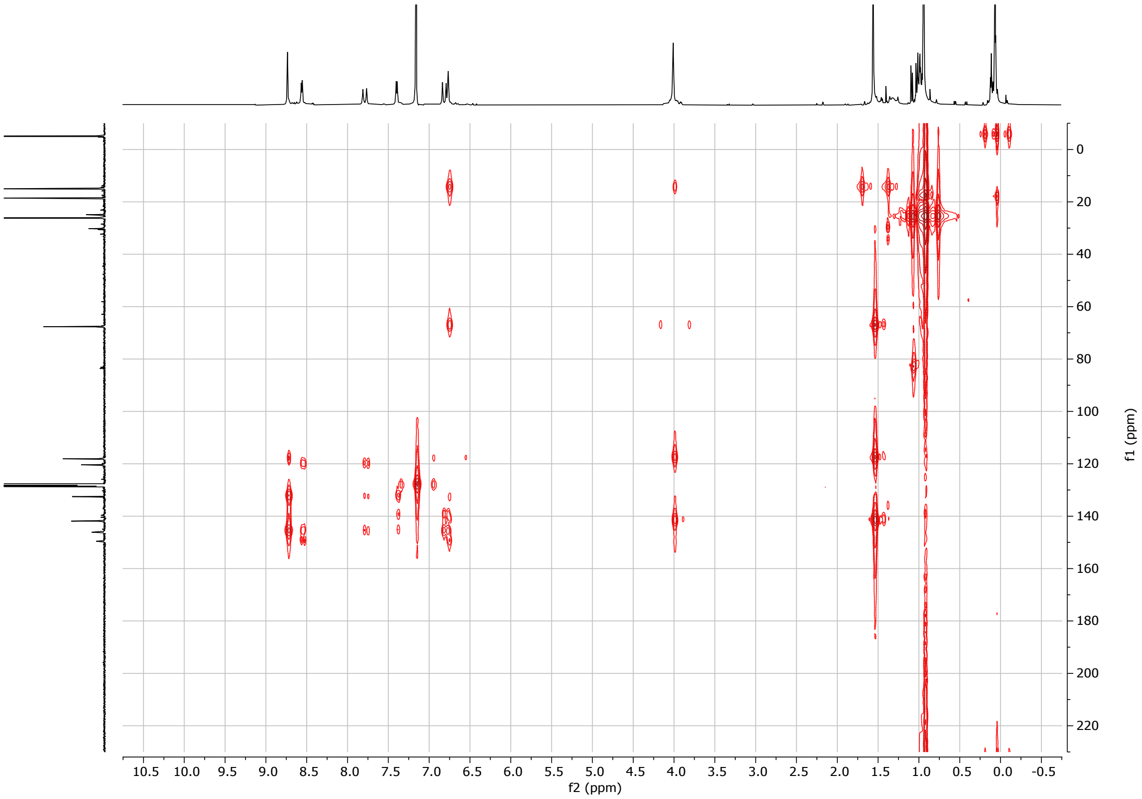

^{1}H-NMR (400.16 MHz, C_6D_6)

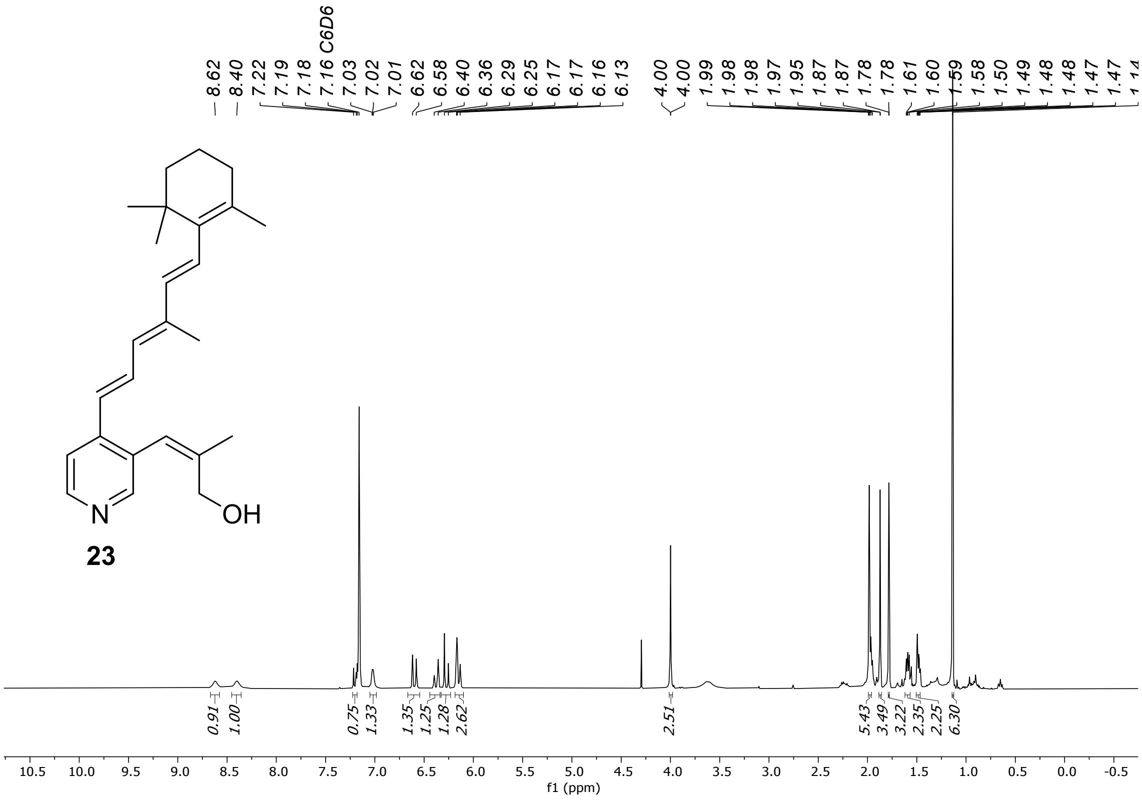

^{13}C-NMR (100.63 MHz, C_6D_6)

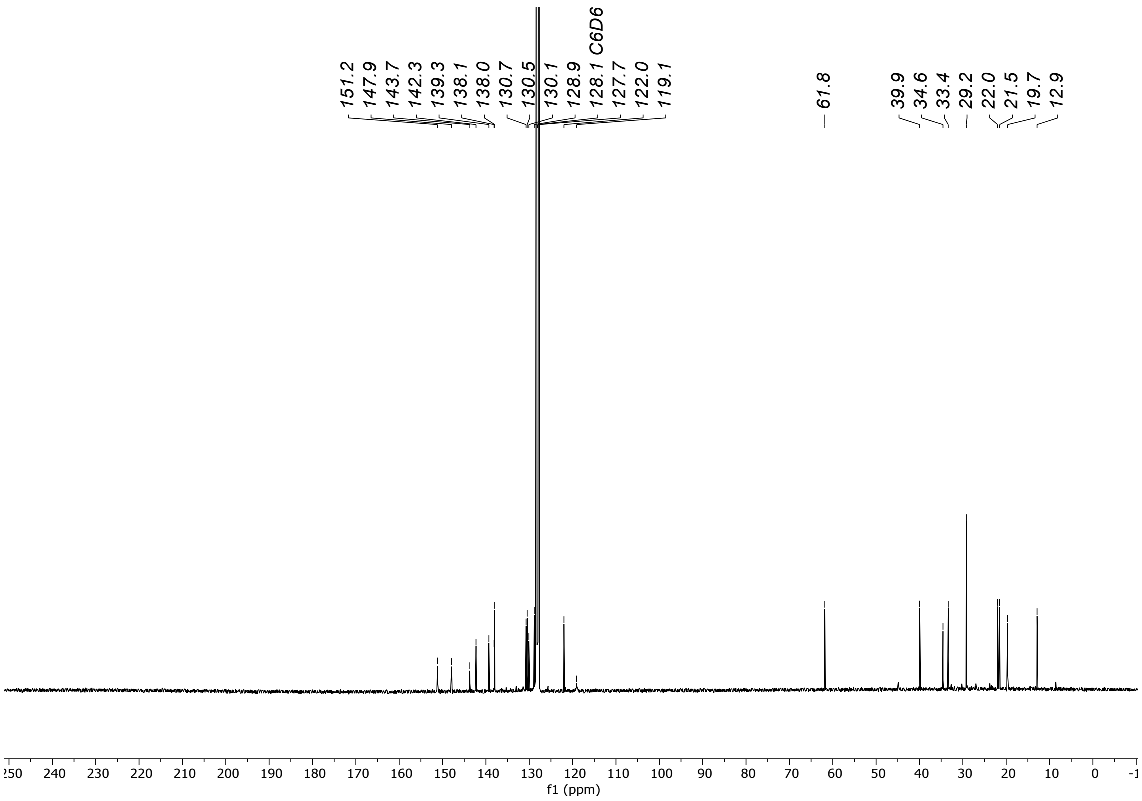

COSY (C_6D_6)

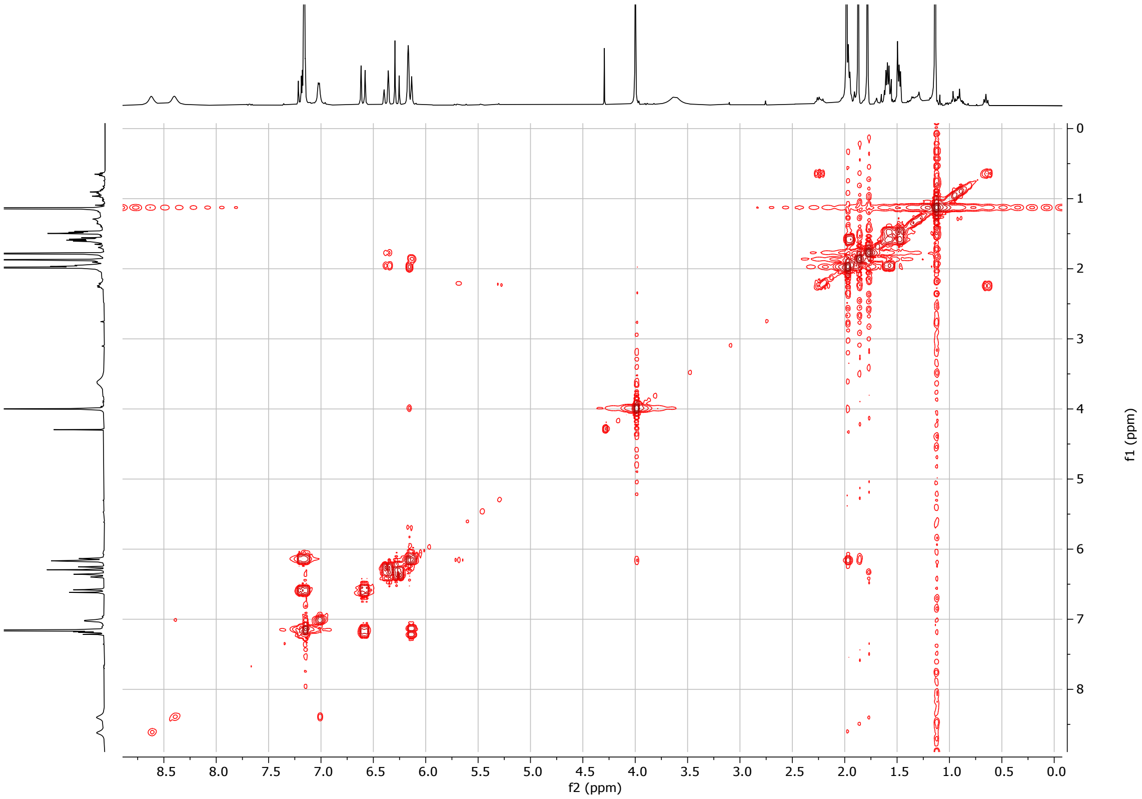

HSQC(C_6D_6)

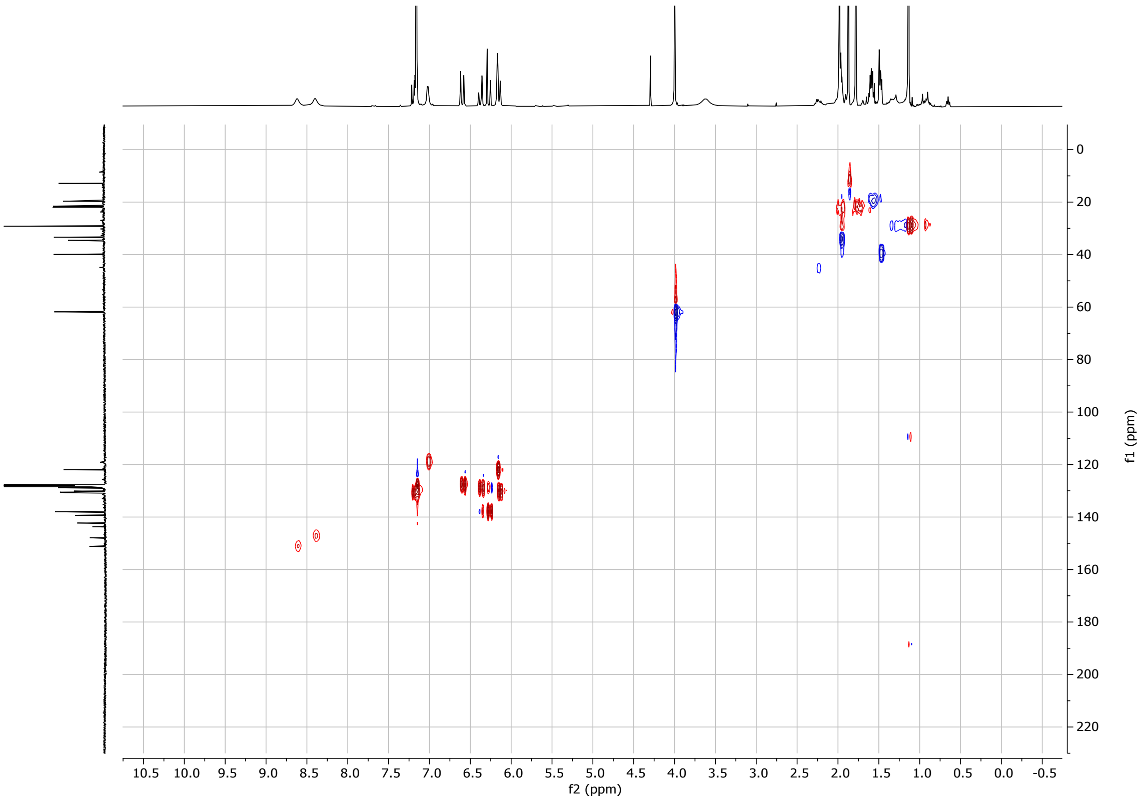

HMBC(C_6D_6)

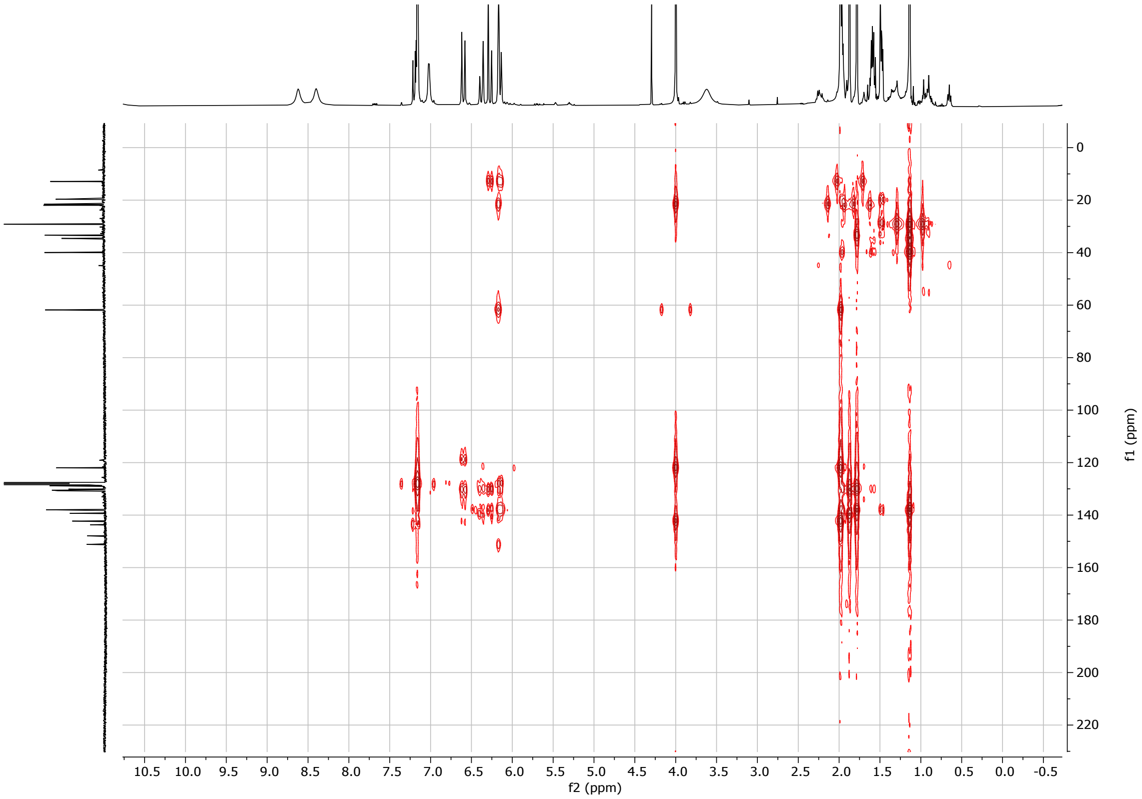

^{1}H-NMR (400.16 MHz, C_6D_6)

24

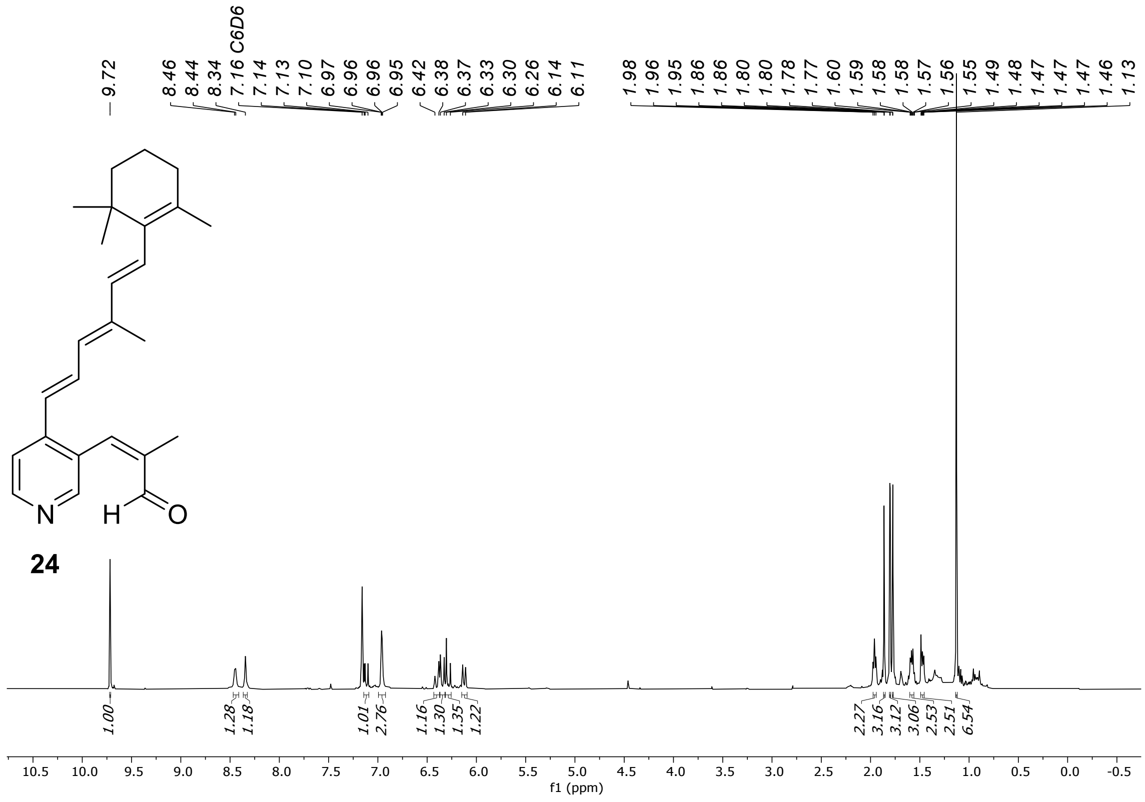

^{13}C-NMR (100.63 MHz, C_6D_6)

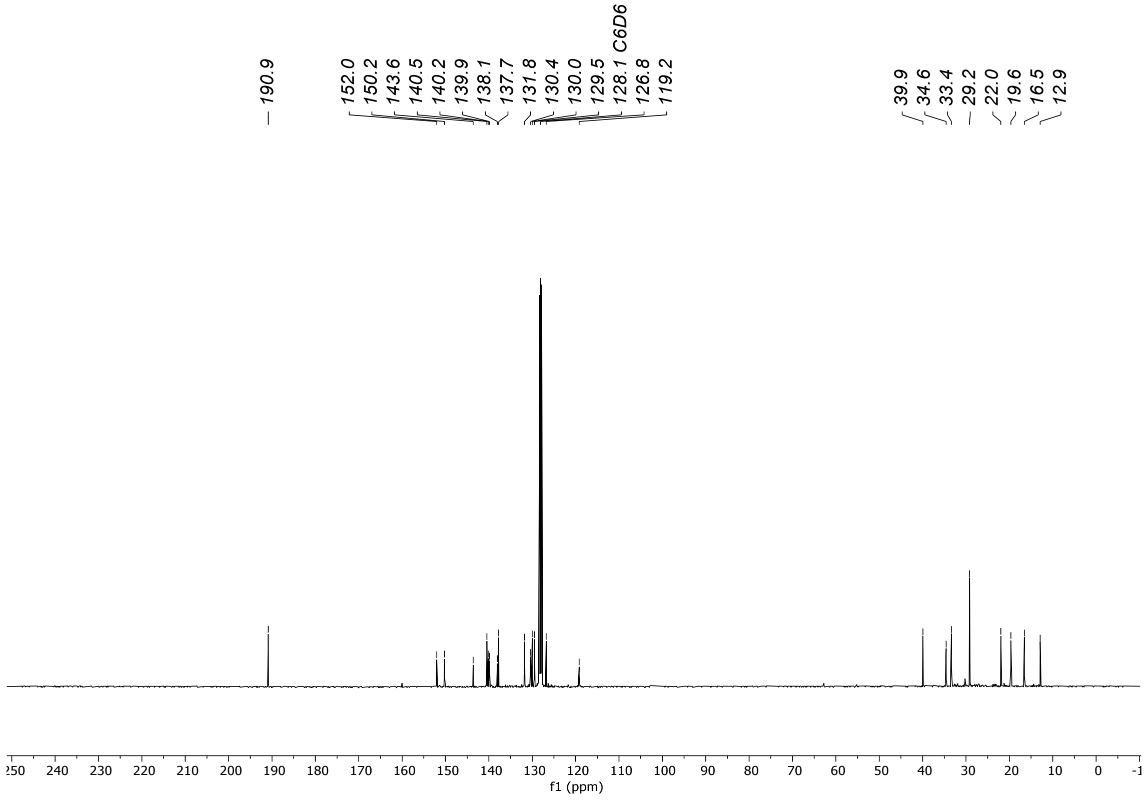

COSY (C_6D_6)

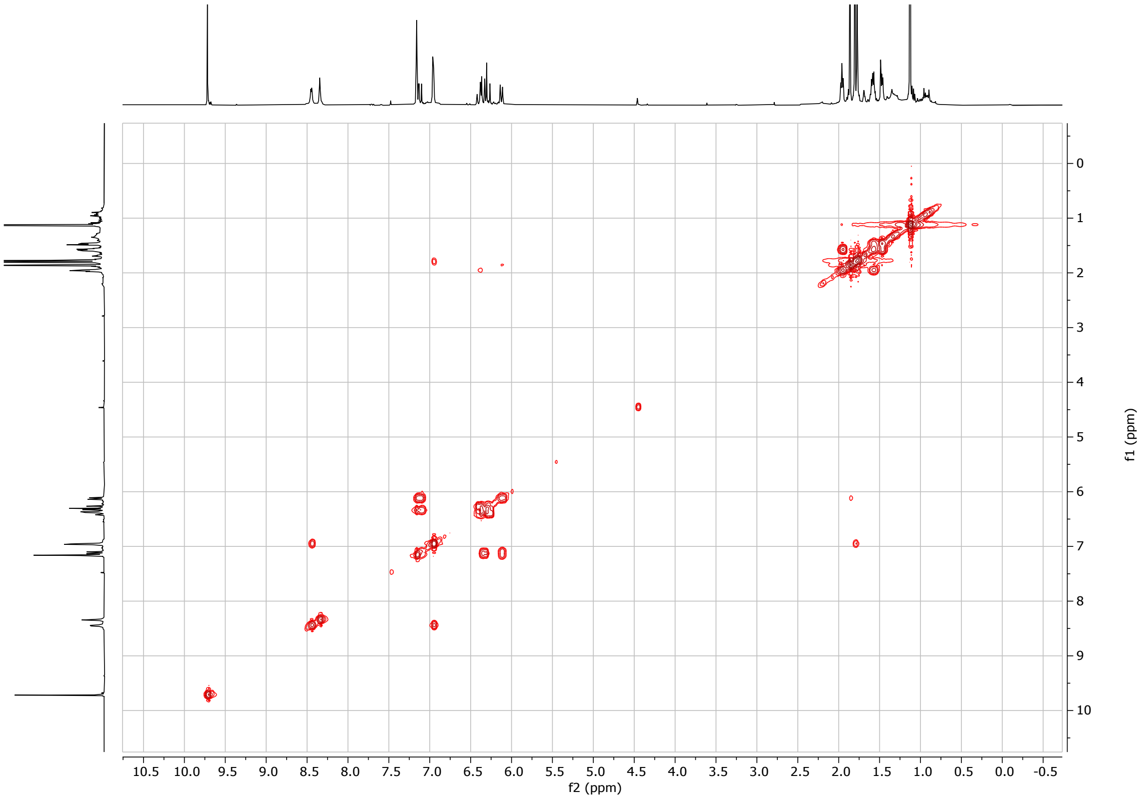

HSQC (C_6D_6)

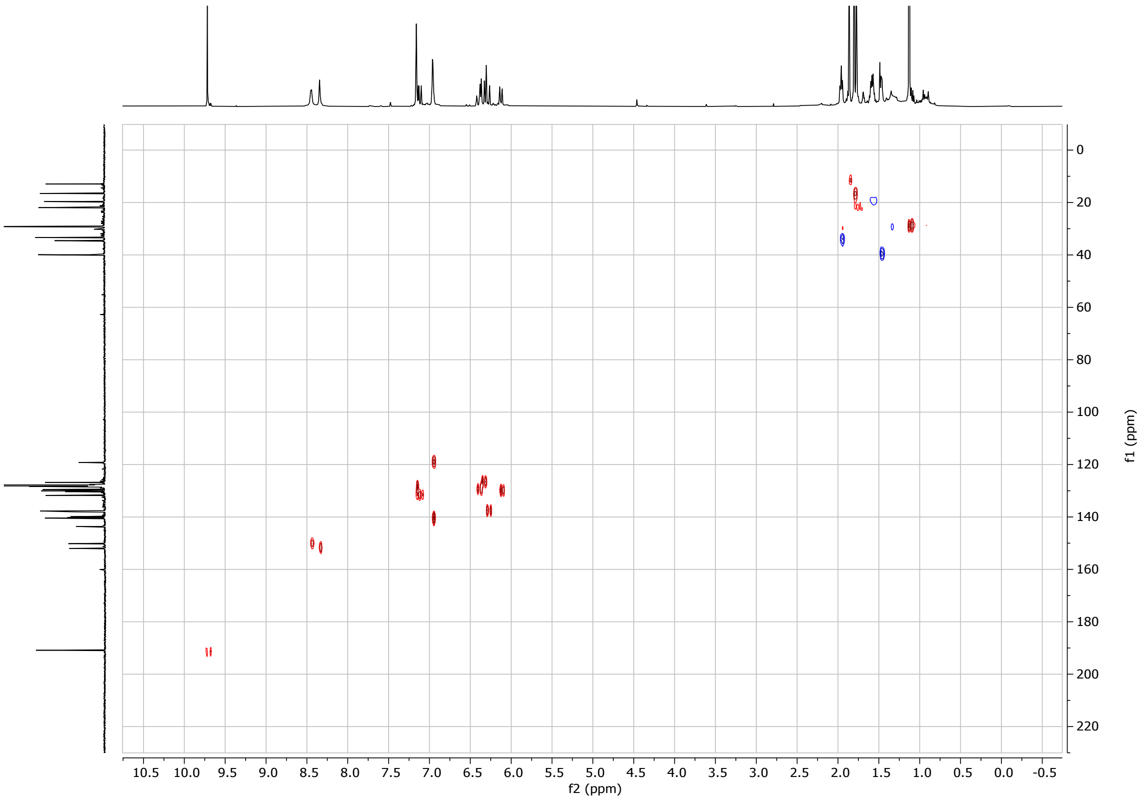

HMBC (C_6D_6)

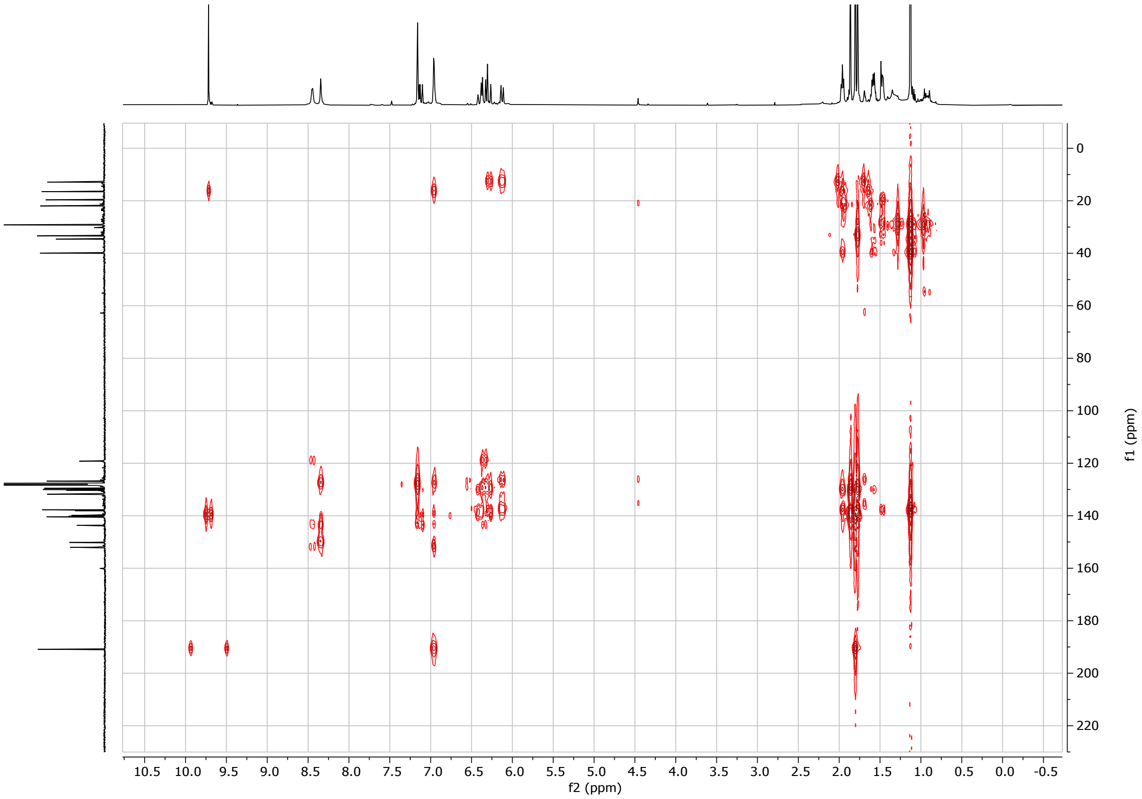

^{1}H-NMR (400.16 MHz, C_6D_6)

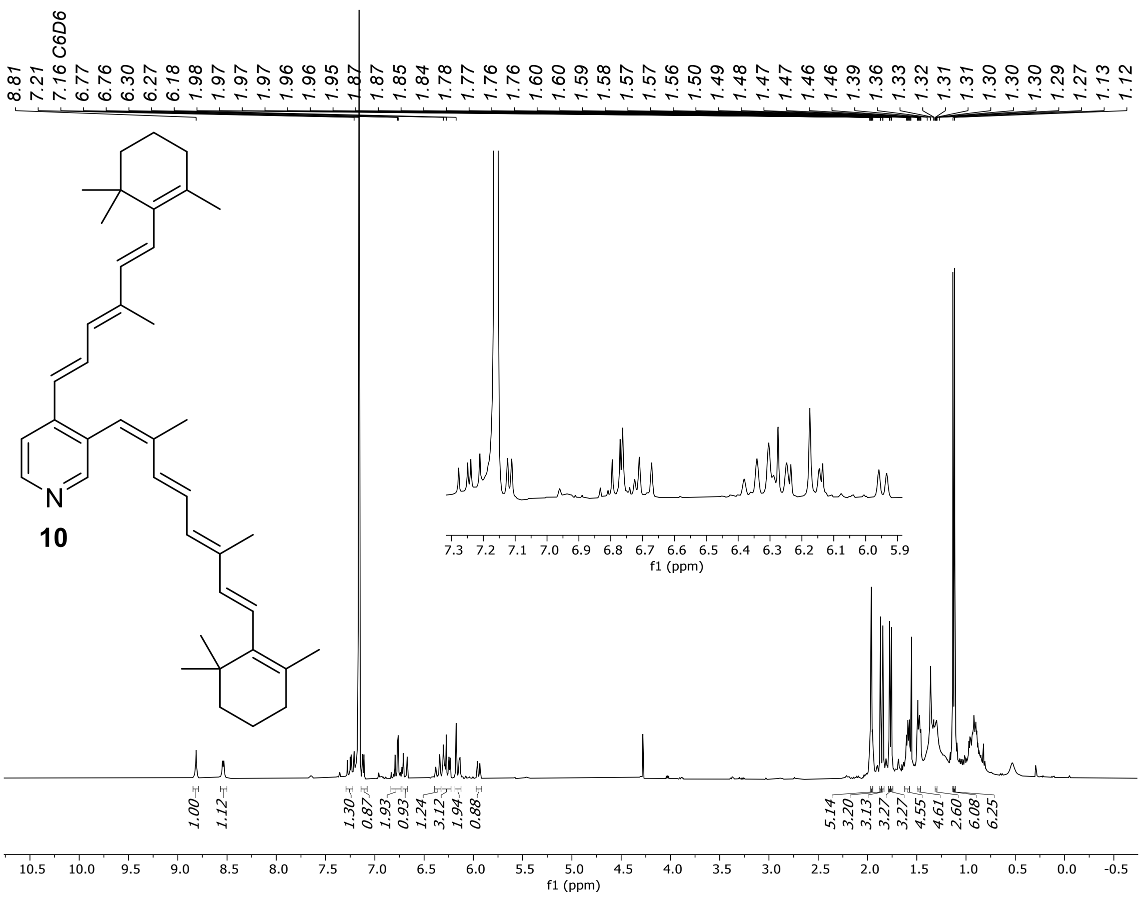

^{13}C-NMR (100.63 MHz, C_6D_6)

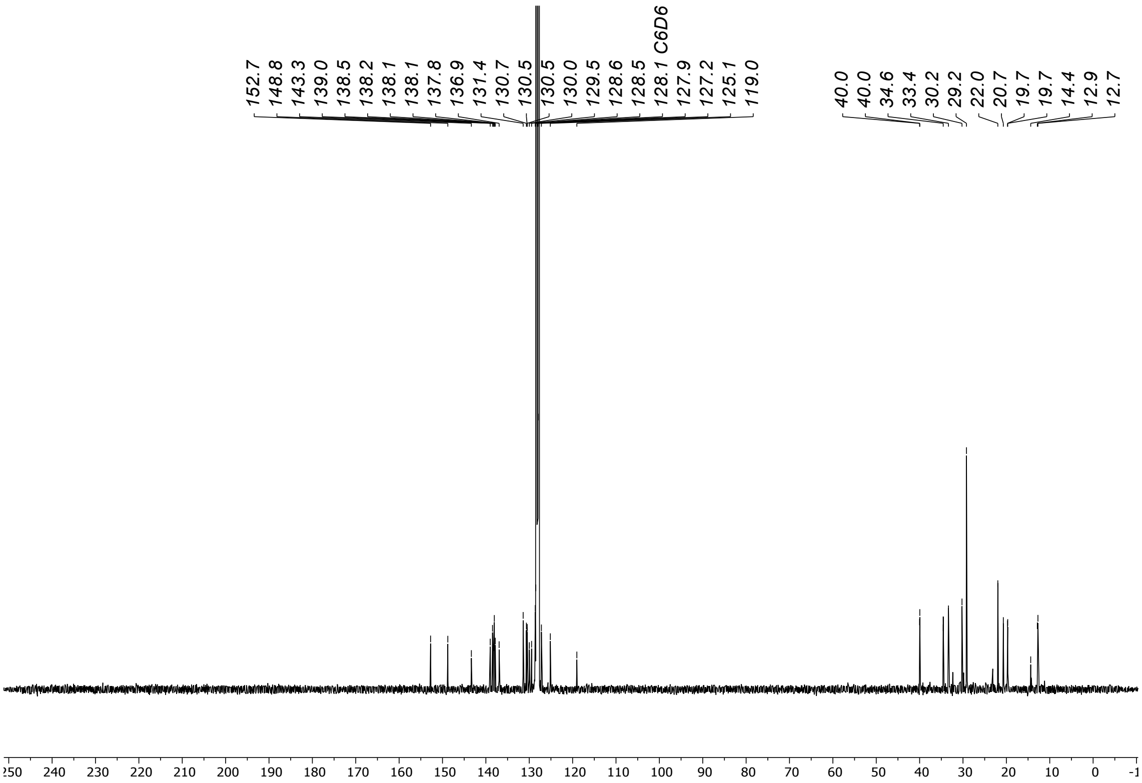

COSY (C_6D_6)

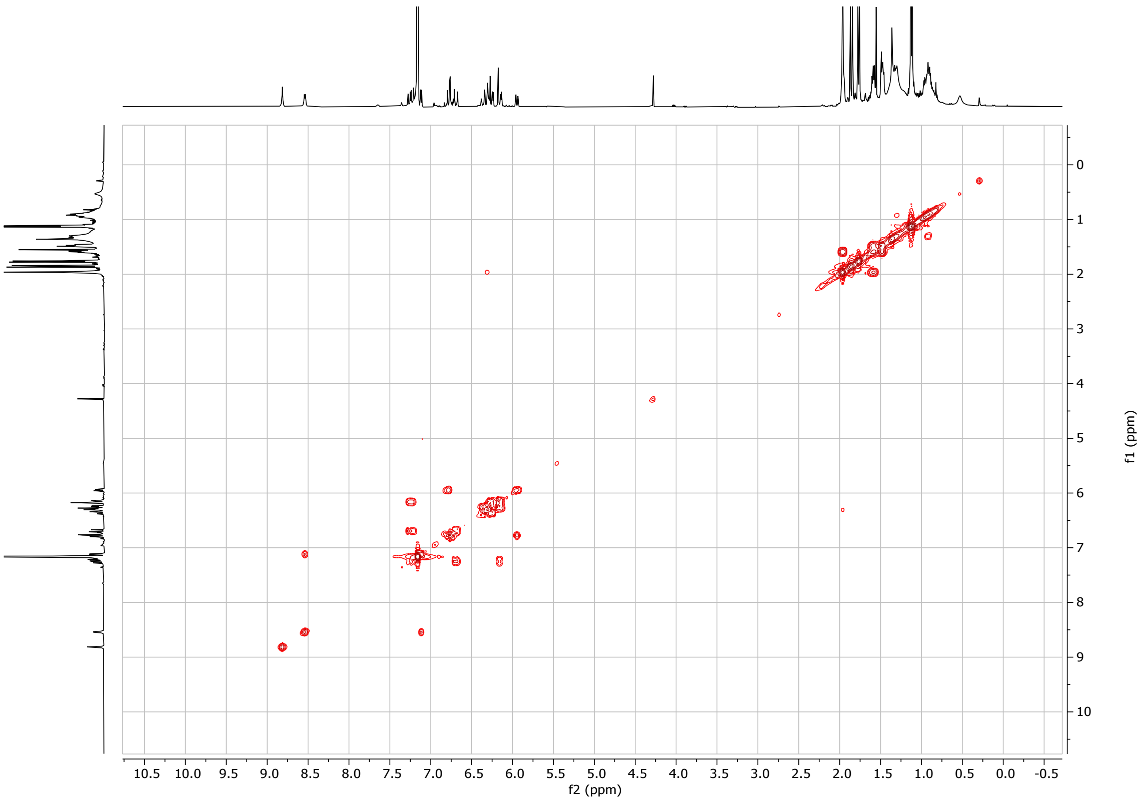

HSQC(C_6D_6)

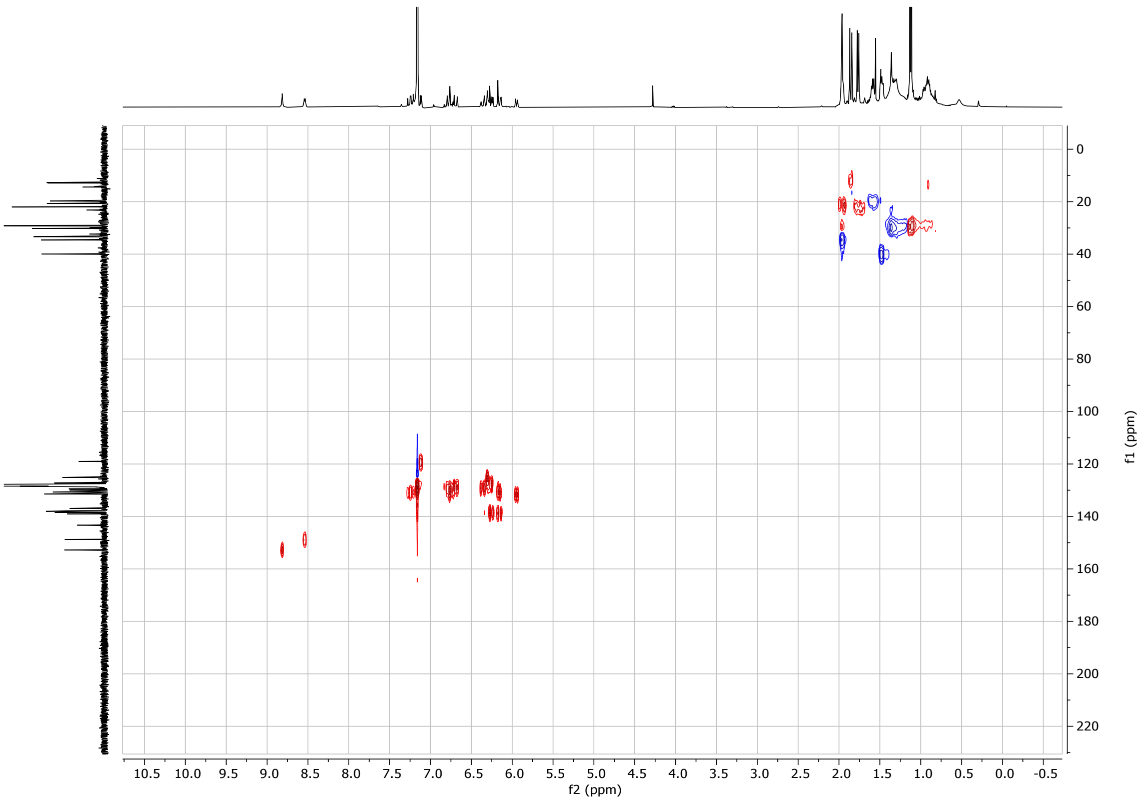

HMBC(C_6D_6)

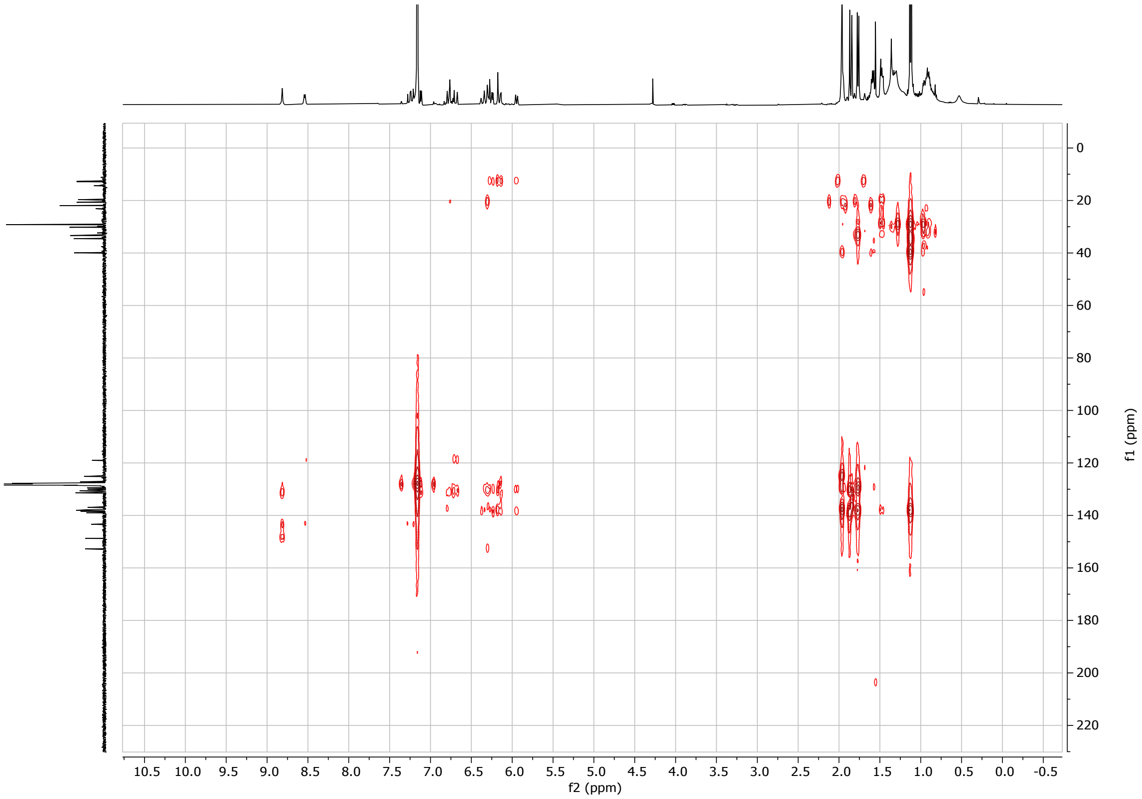

^{1}H-NMR (400.16 MHz, CD_3OD)

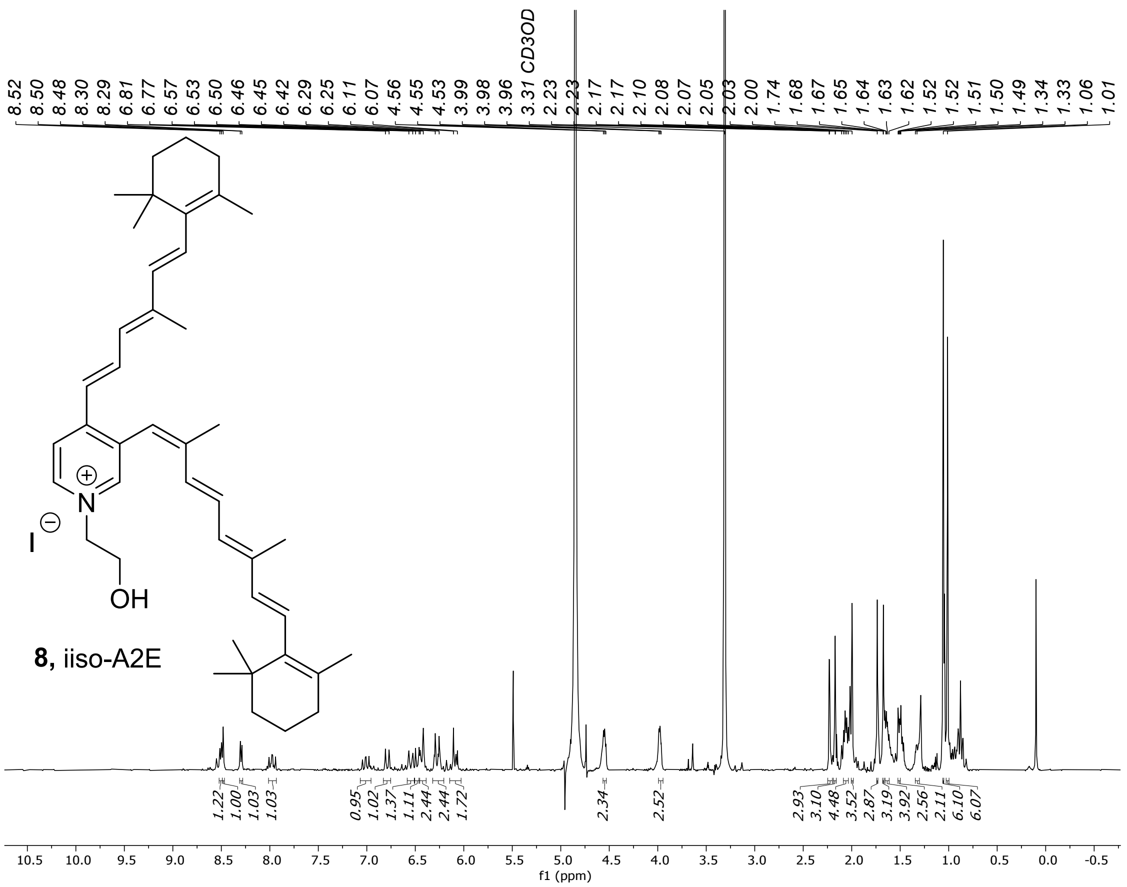

^{13}C-NMR (100.63 MHz, CD_3OD)

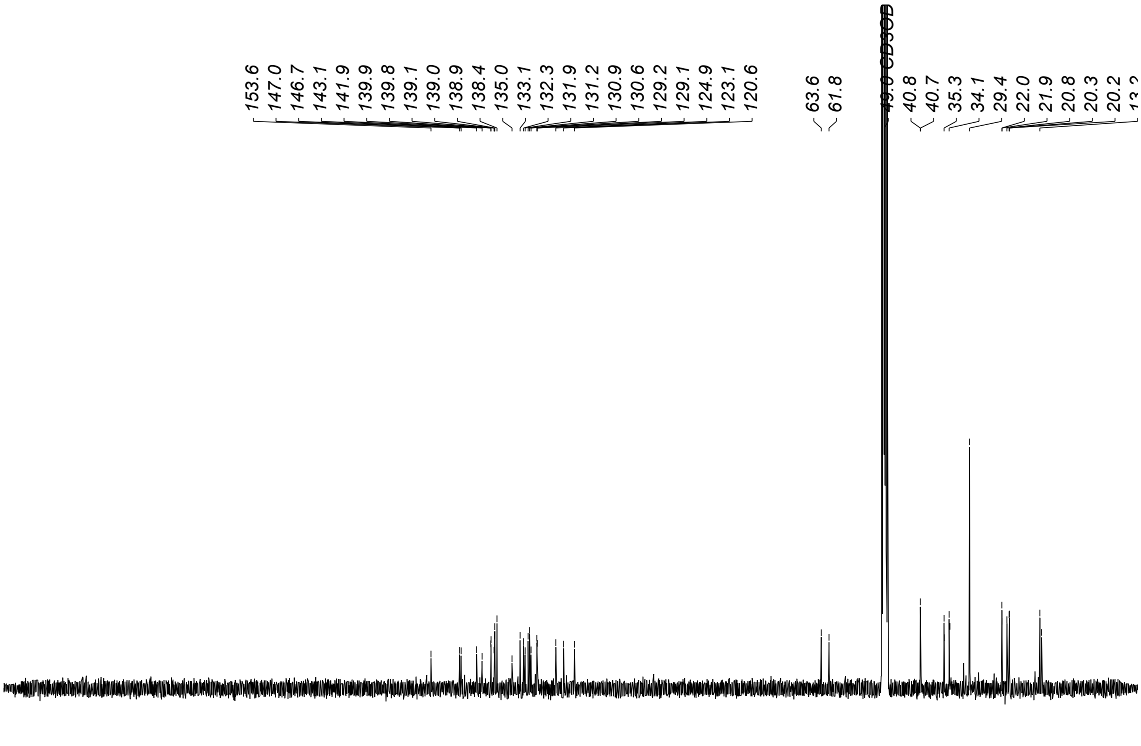

COSY (CD_3OD)

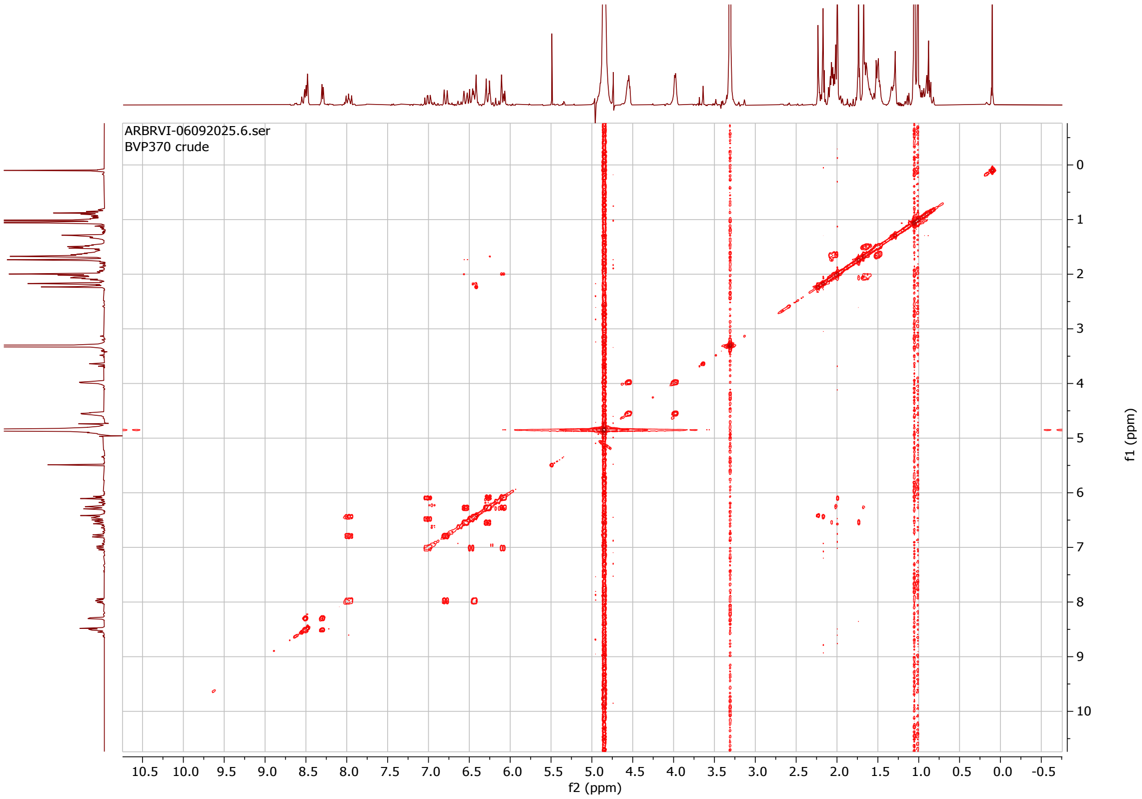

HSQC (CD_3OD)

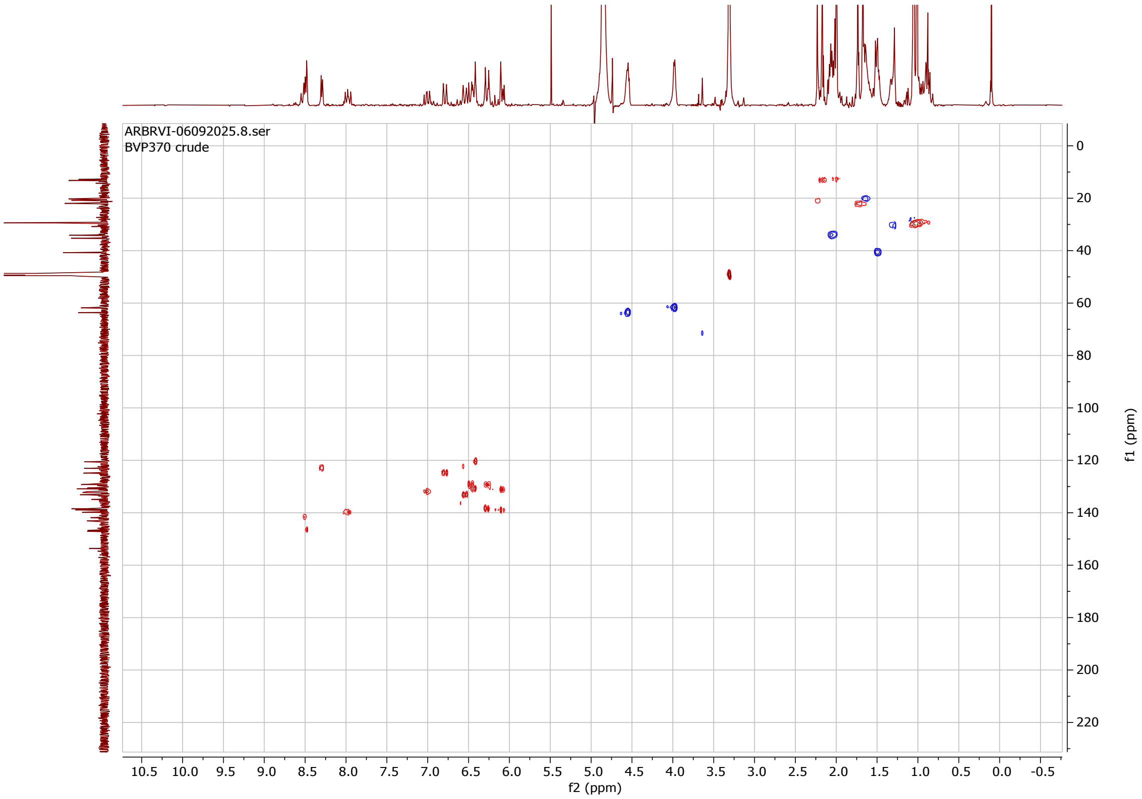

HMBC (CD_3OD)

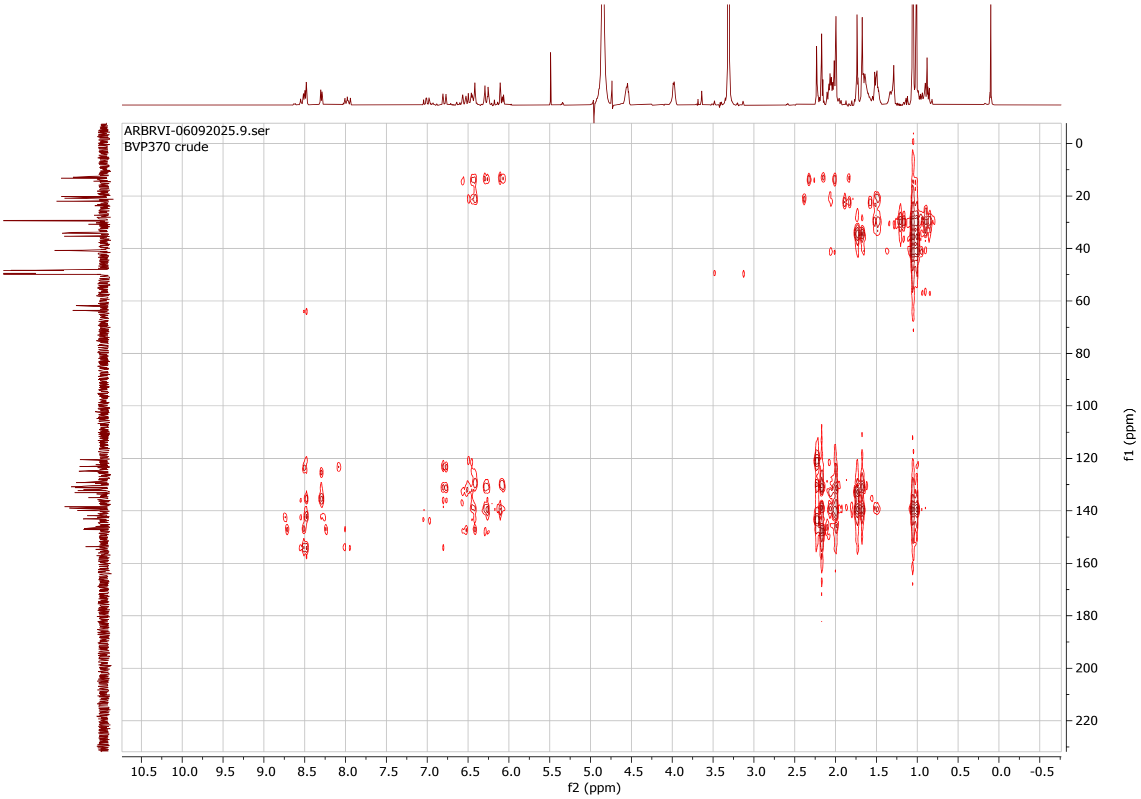

NOE-1D (400.16 MHz, freq. 7.99 ppm, CD_3OD)

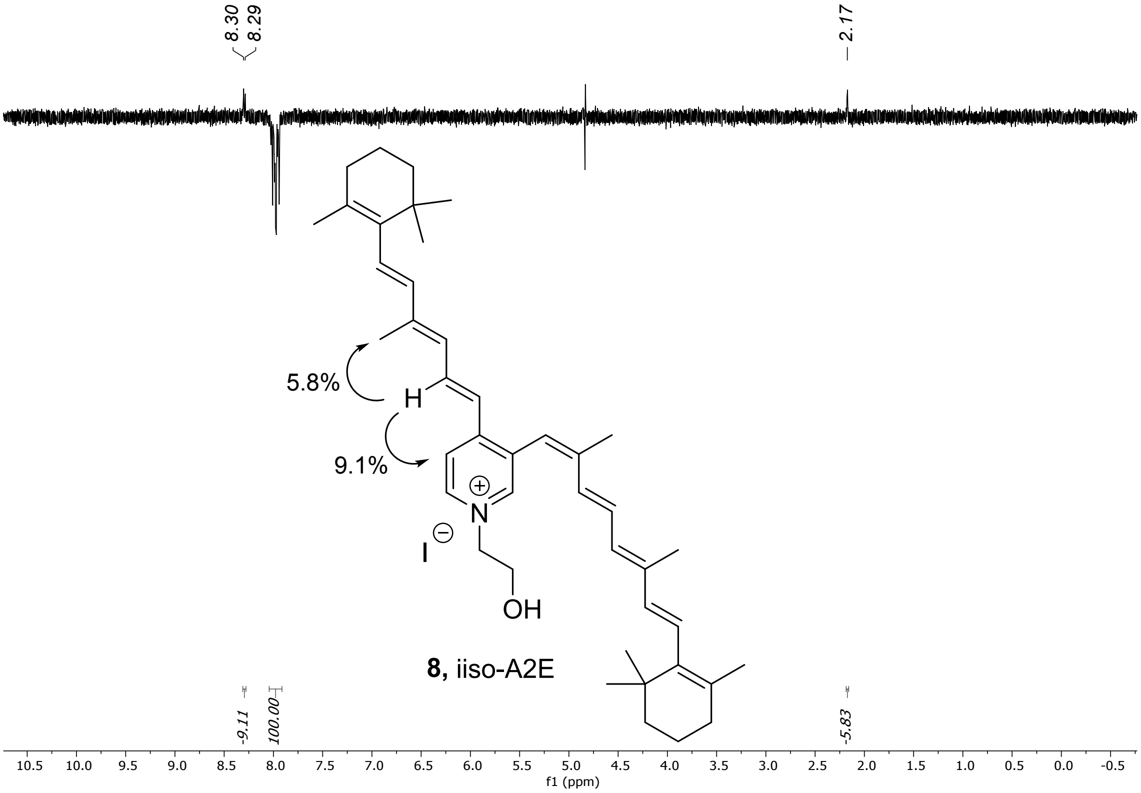

8, iiso-A2E

NOE-1D (400.16 MHz, freq. 2.23 ppm, CD_3OD)

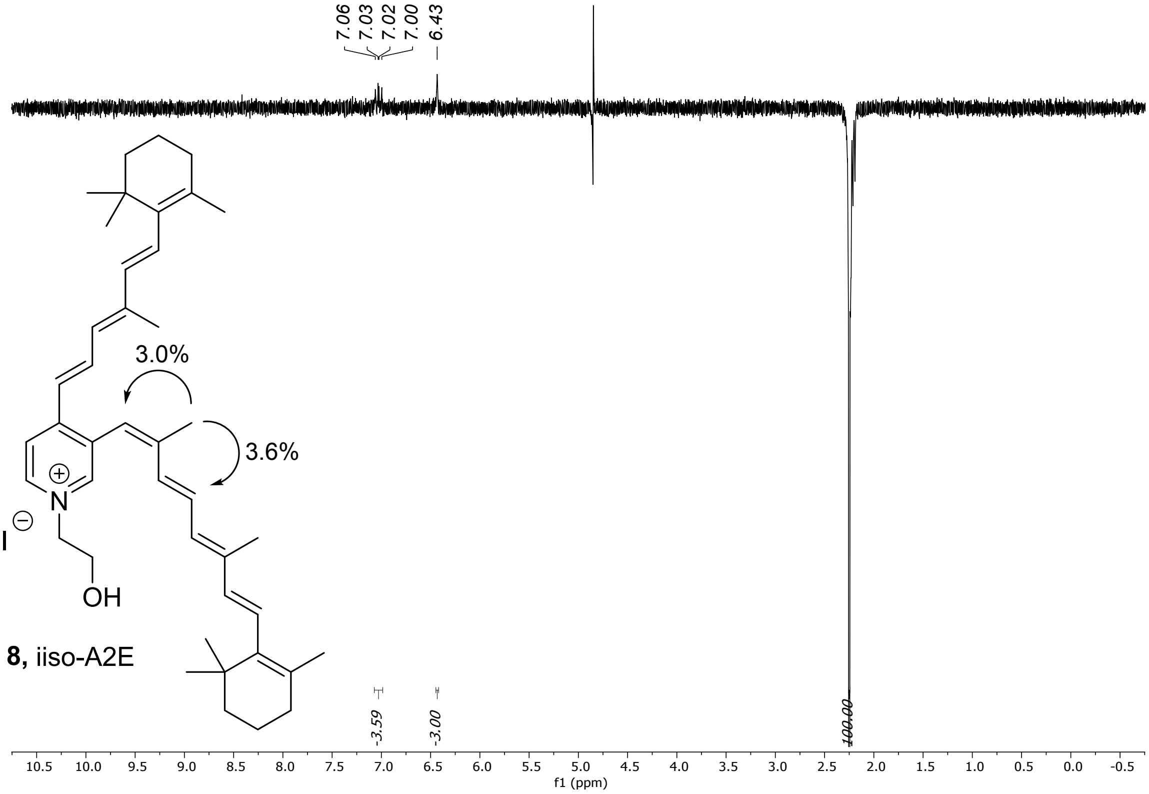

NOE-1D (400.16 MHz, freq. 2.17 ppm, CD_3OD)

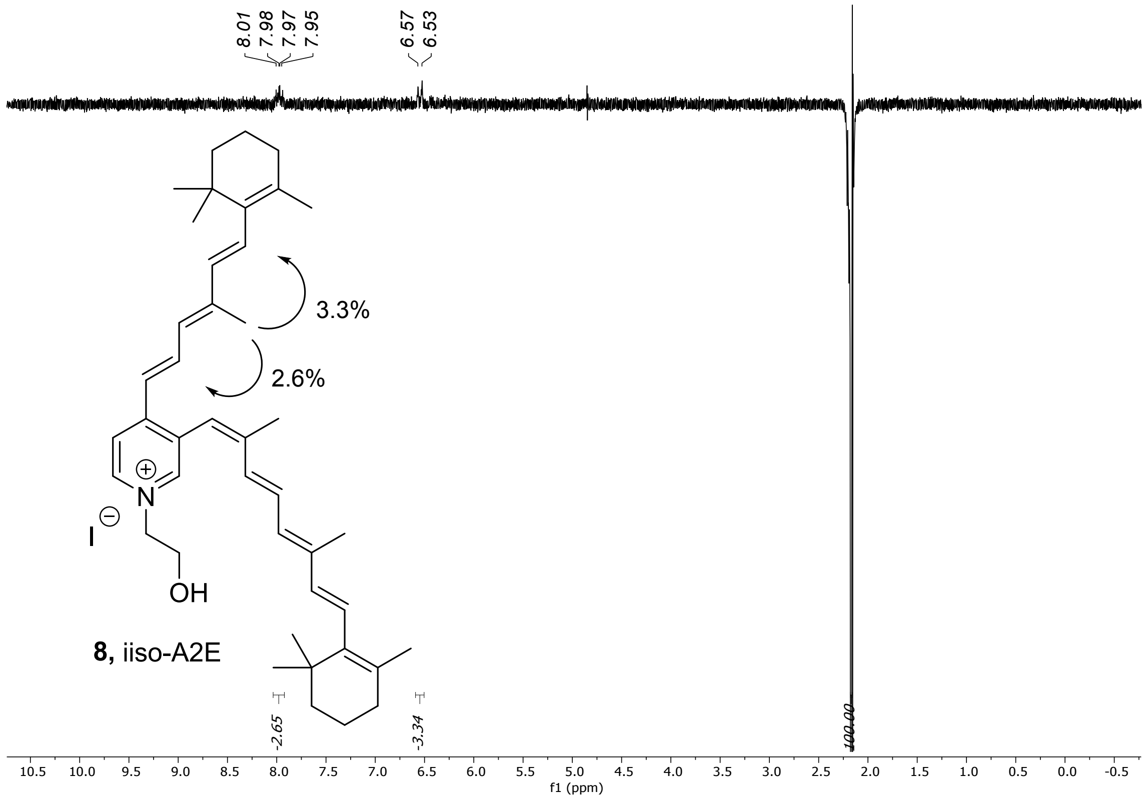

^{1}H-NMR (400.16 MHz, C_6D_6)

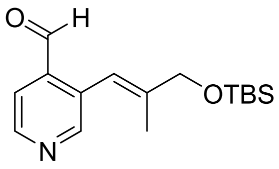

29

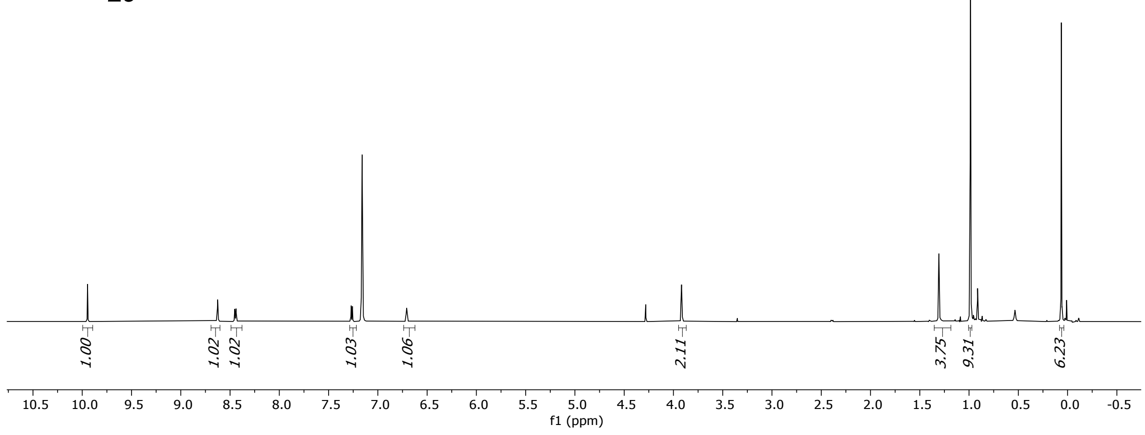

^{13}C-NMR (100.63 MHz, C_6D_6)

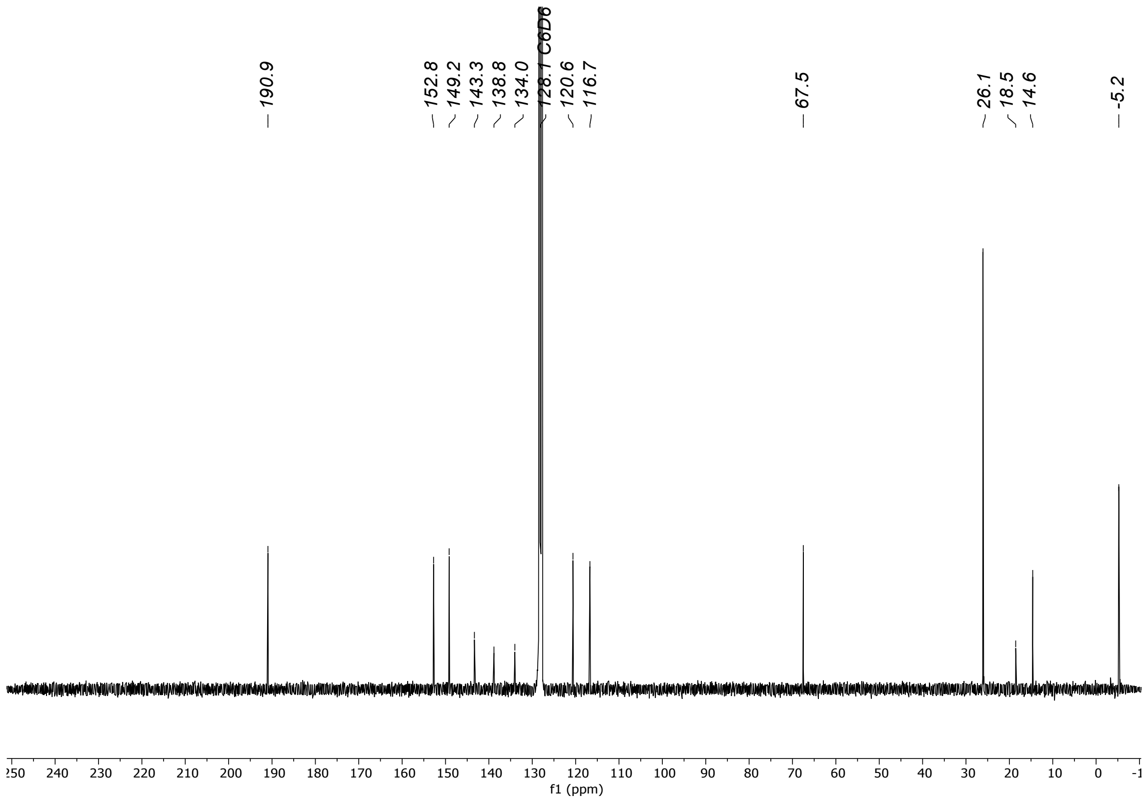

COSY (C_6D_6)

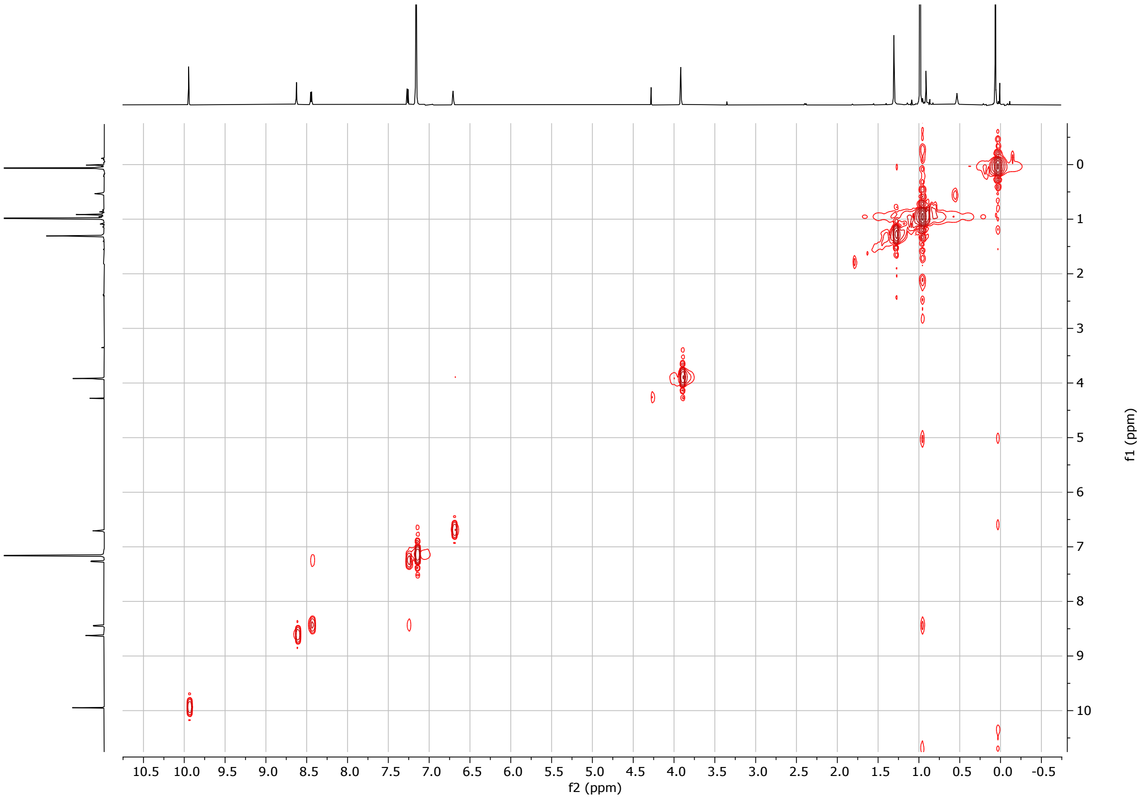

HSQC (C_6D_6)

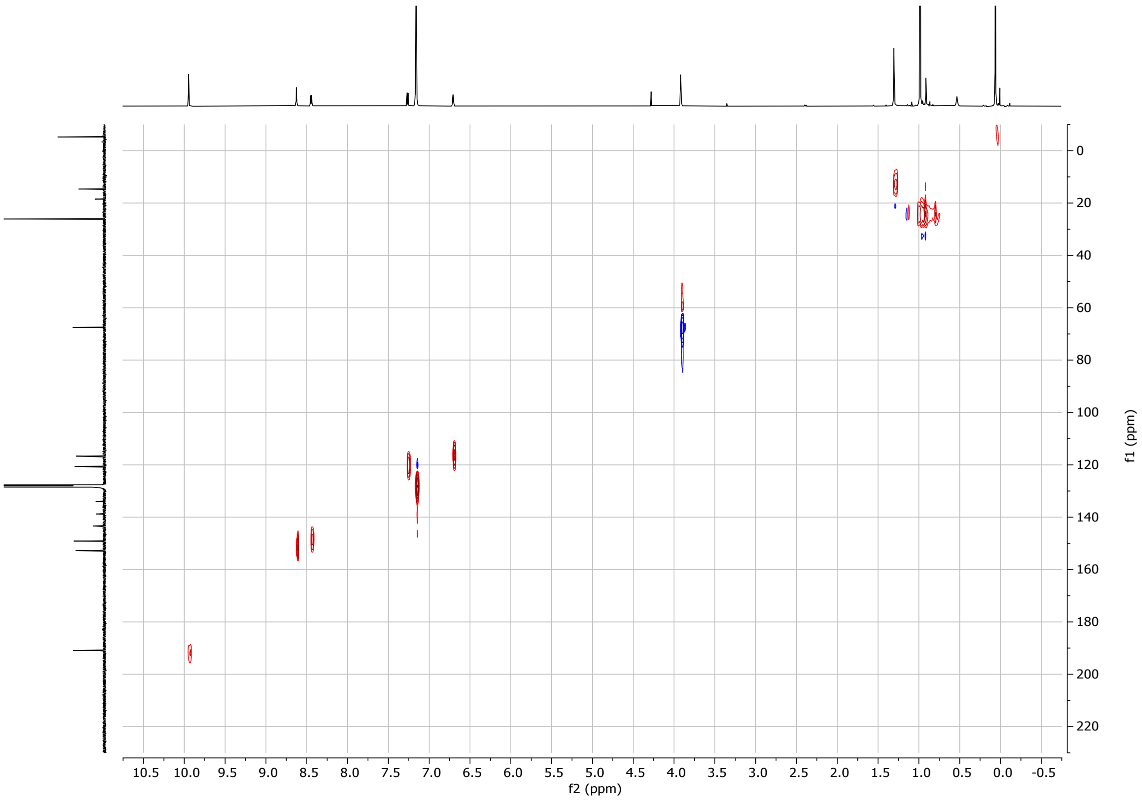

HMBC (C_6D_6)

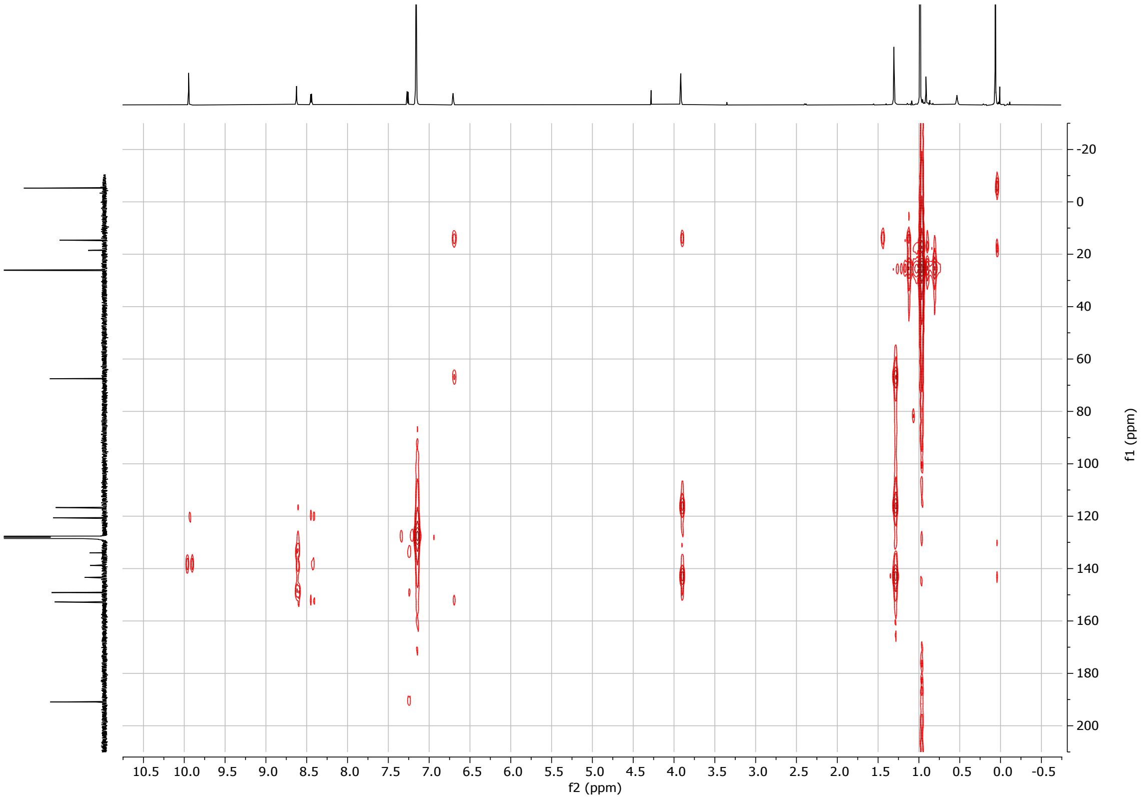

^{1}H-NMR (400.16 MHz, C_6D_6)

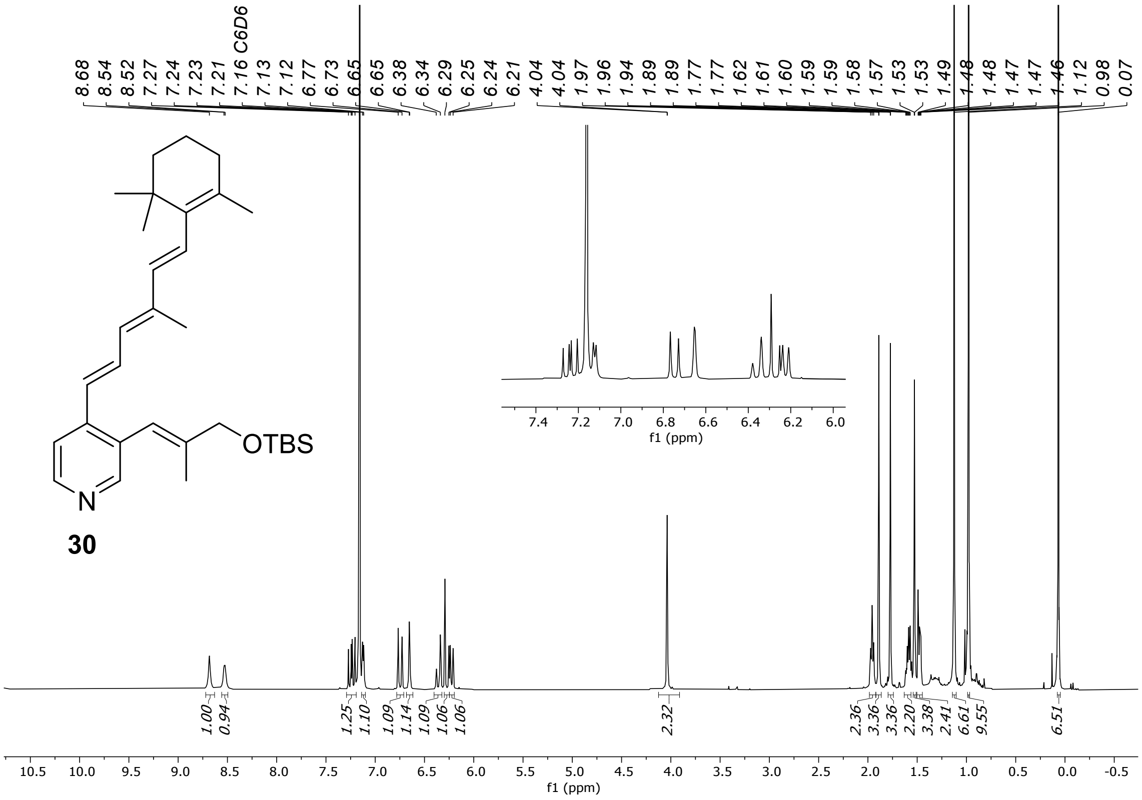

^{13}C-NMR (100.63 MHz, C_6D_6)

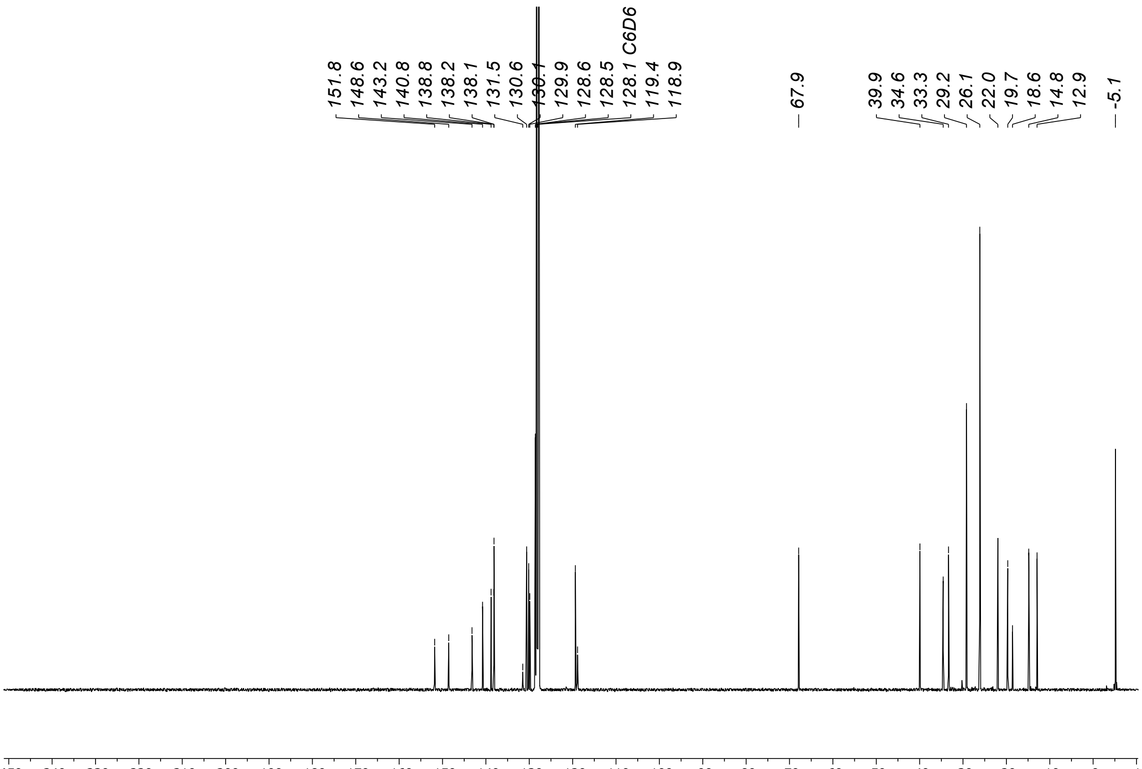

COSY (C_6D_6)

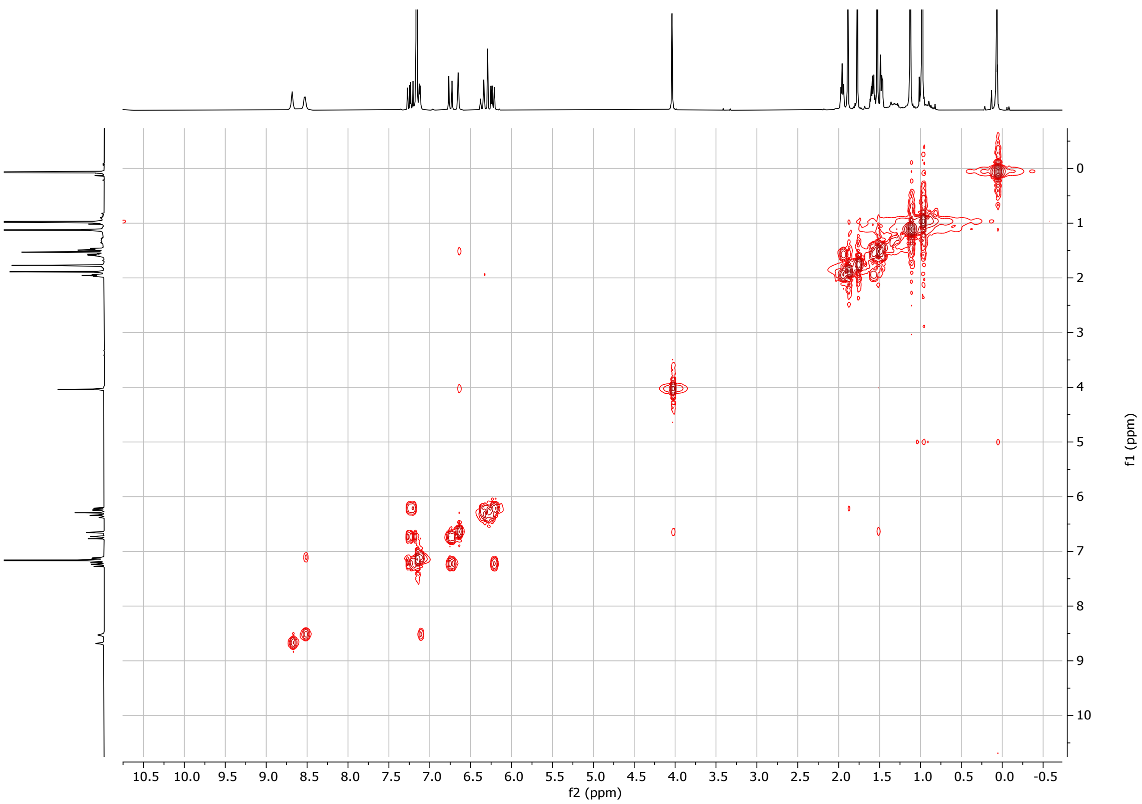

HSQC (C_6D_6)

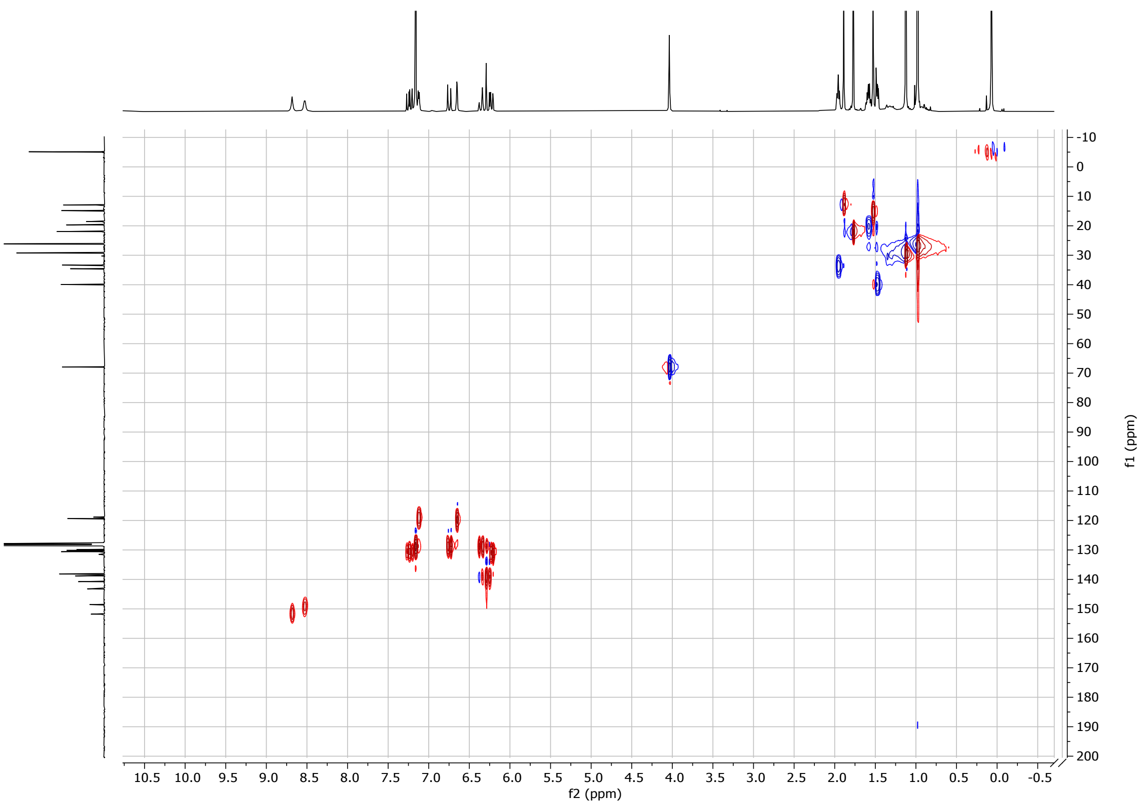

HMBC(C_6D_6)

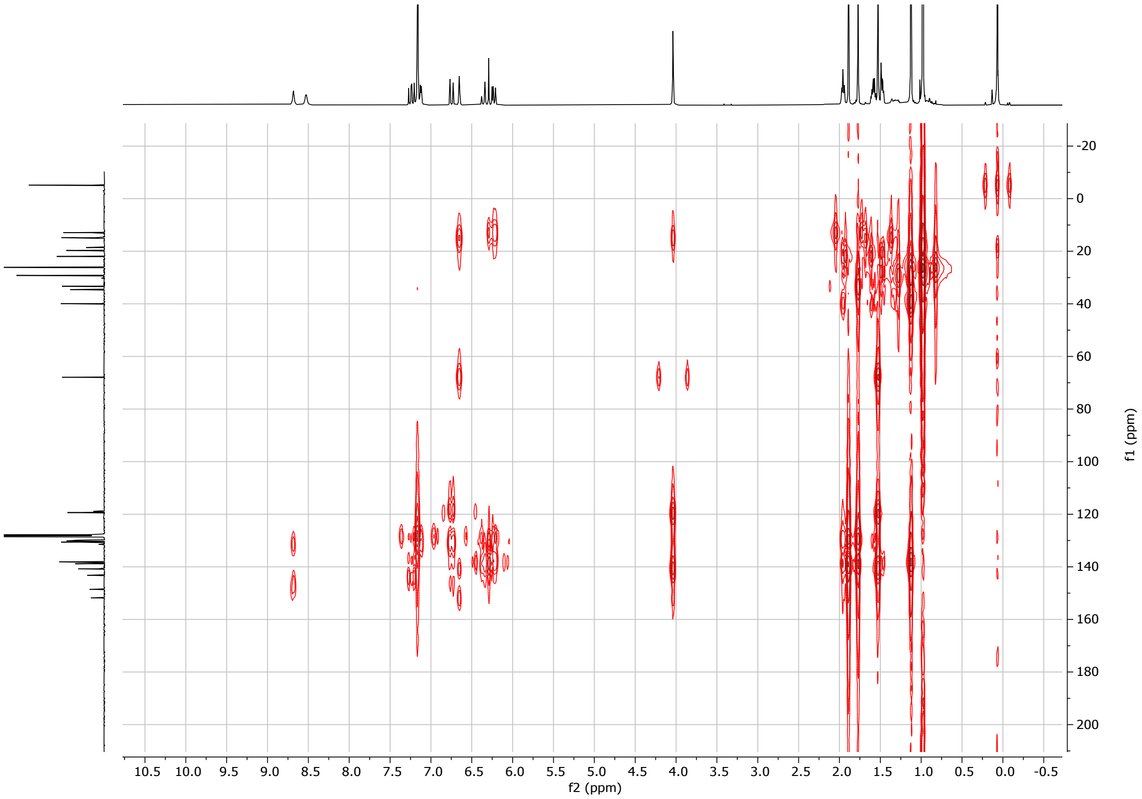

^{1}H-NMR (400.16 MHz, C_6D_6)

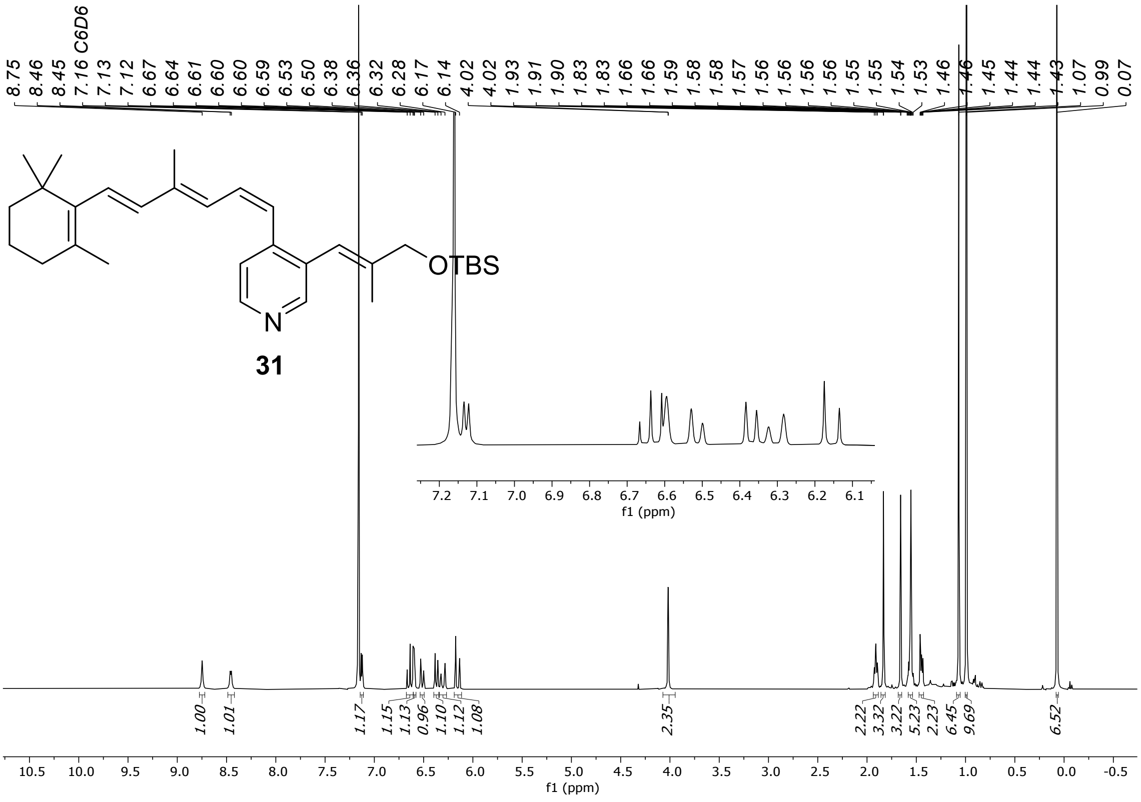

31

^{13}C-NMR (100.63 MHz, C_6D_6)

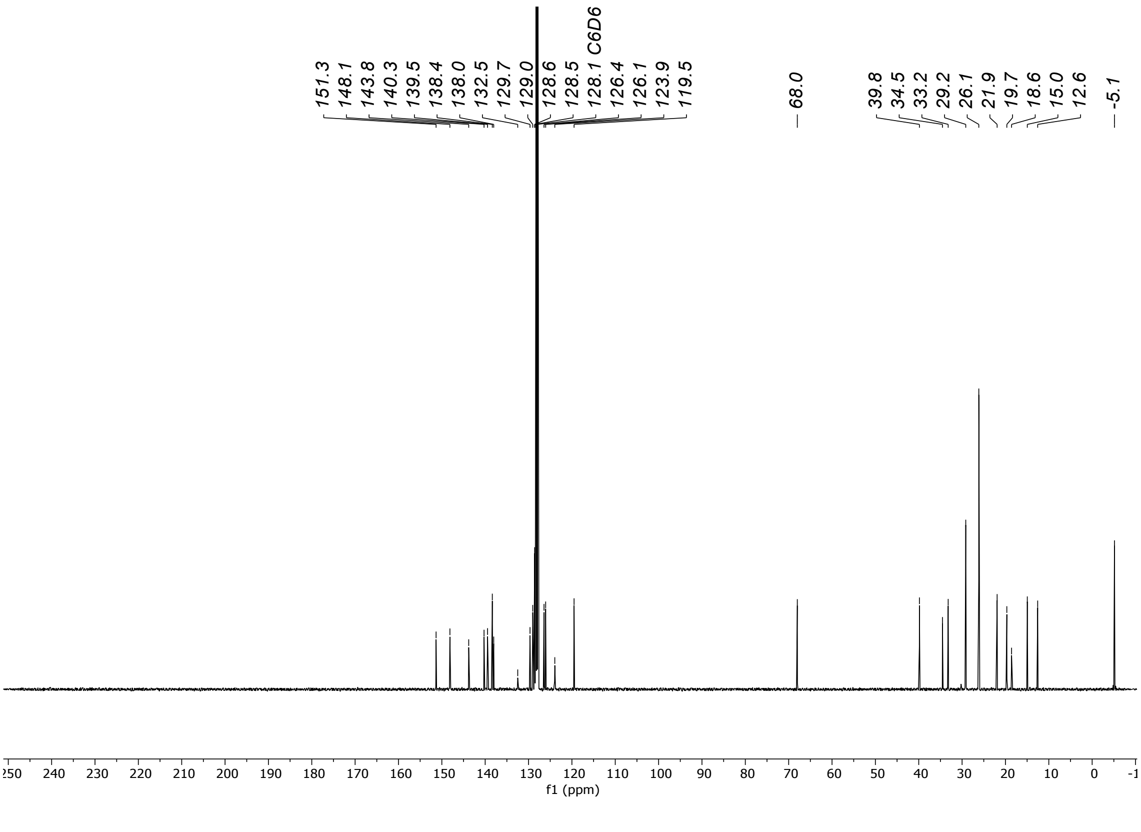

COSY (C_6D_6)

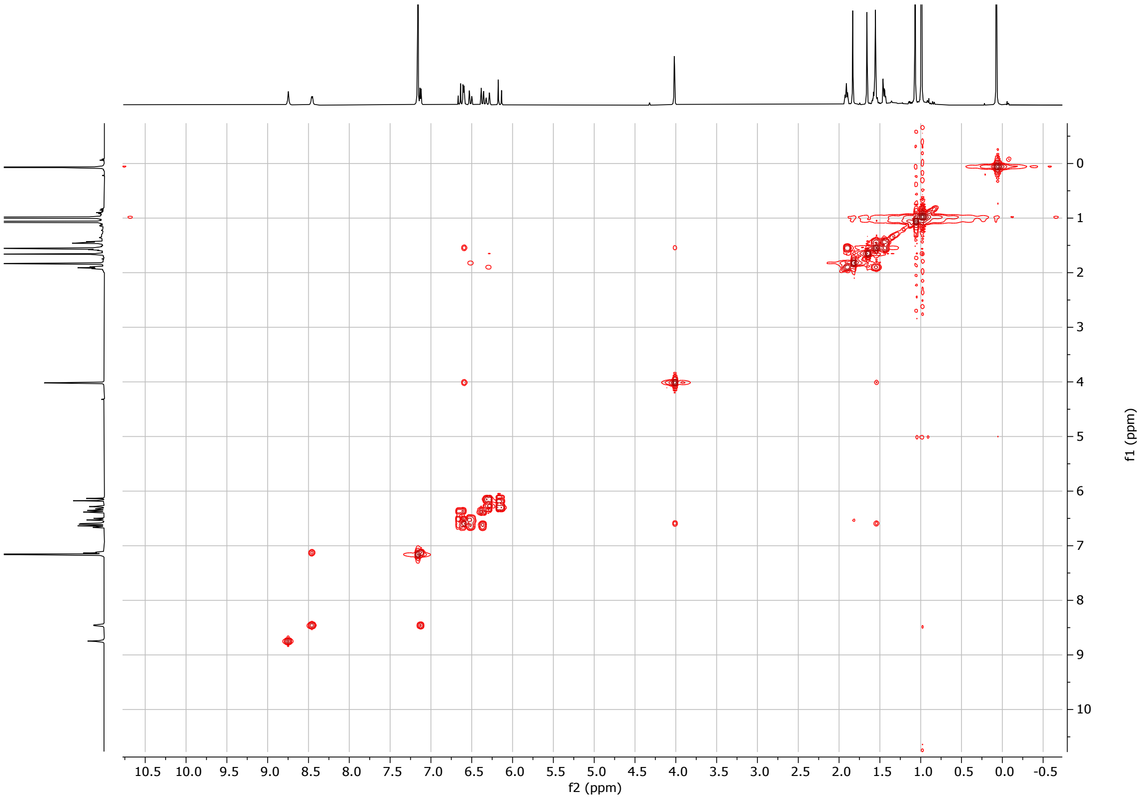

HSQC (C_6D_6)

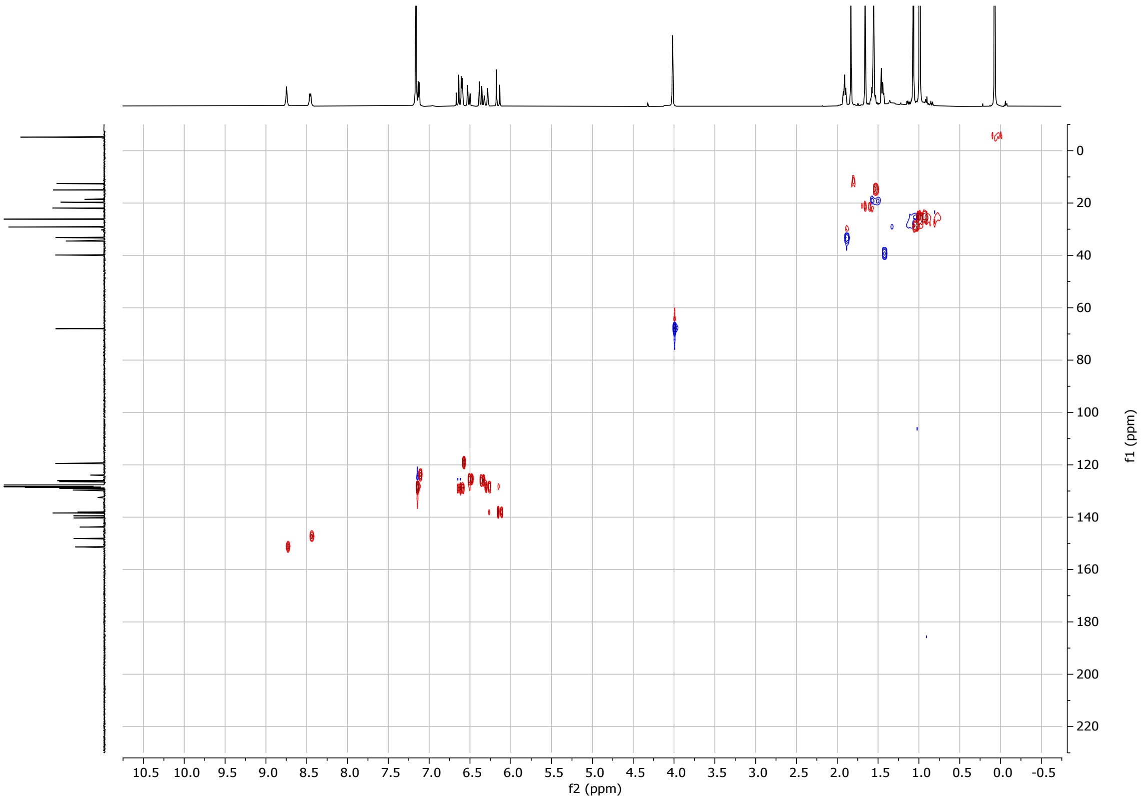

HMBC (C_6D_6)

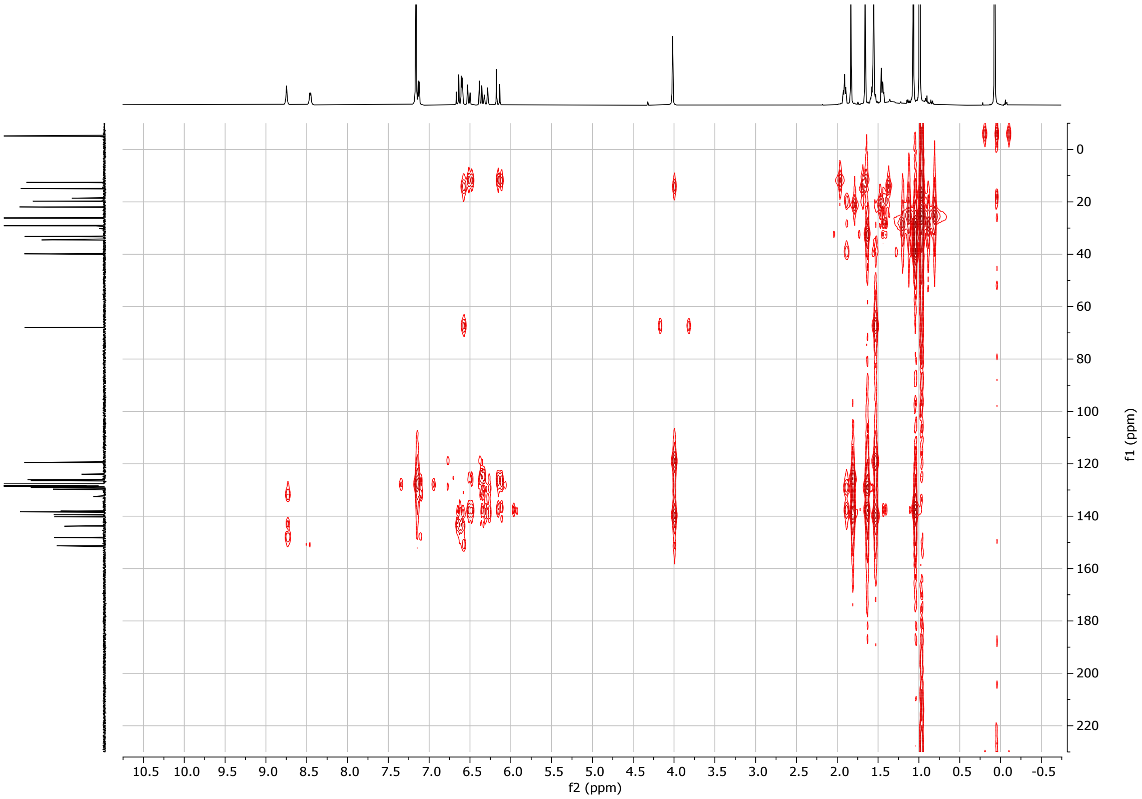

^{1}H-NMR (400.16 MHz, C_6D_6)

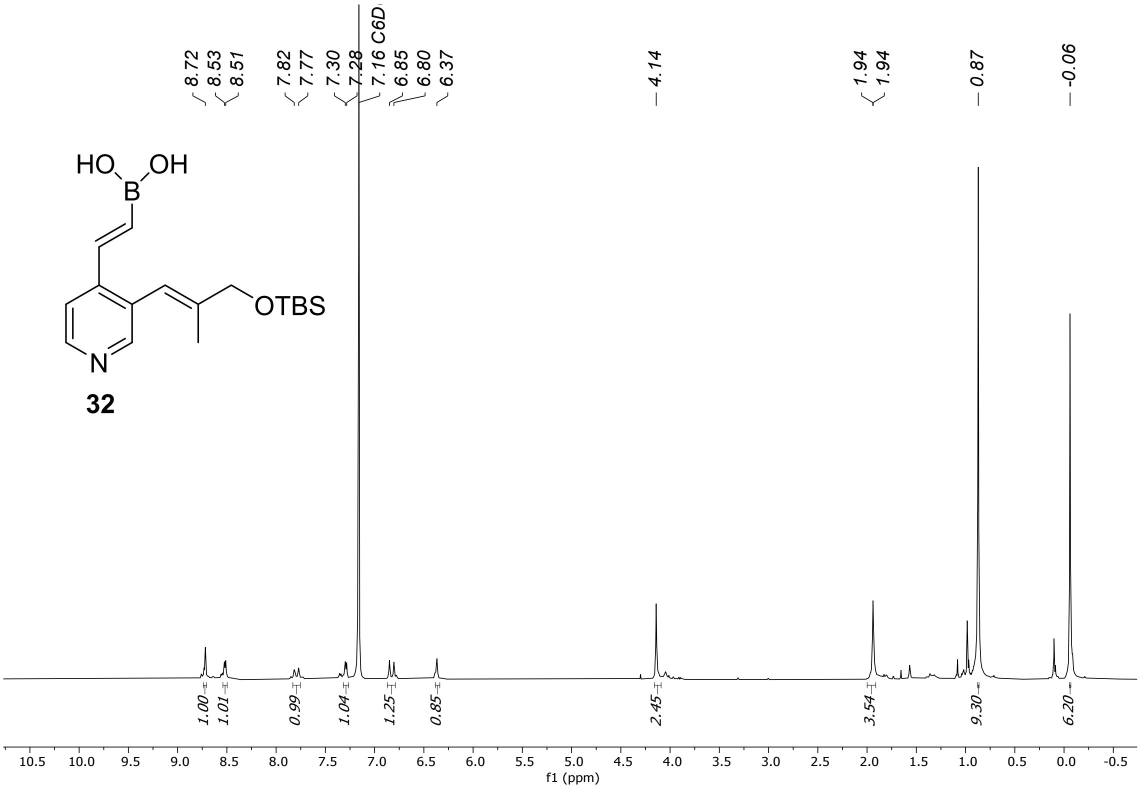

^{13}C-NMR (100.63 MHz, C_6D_6)

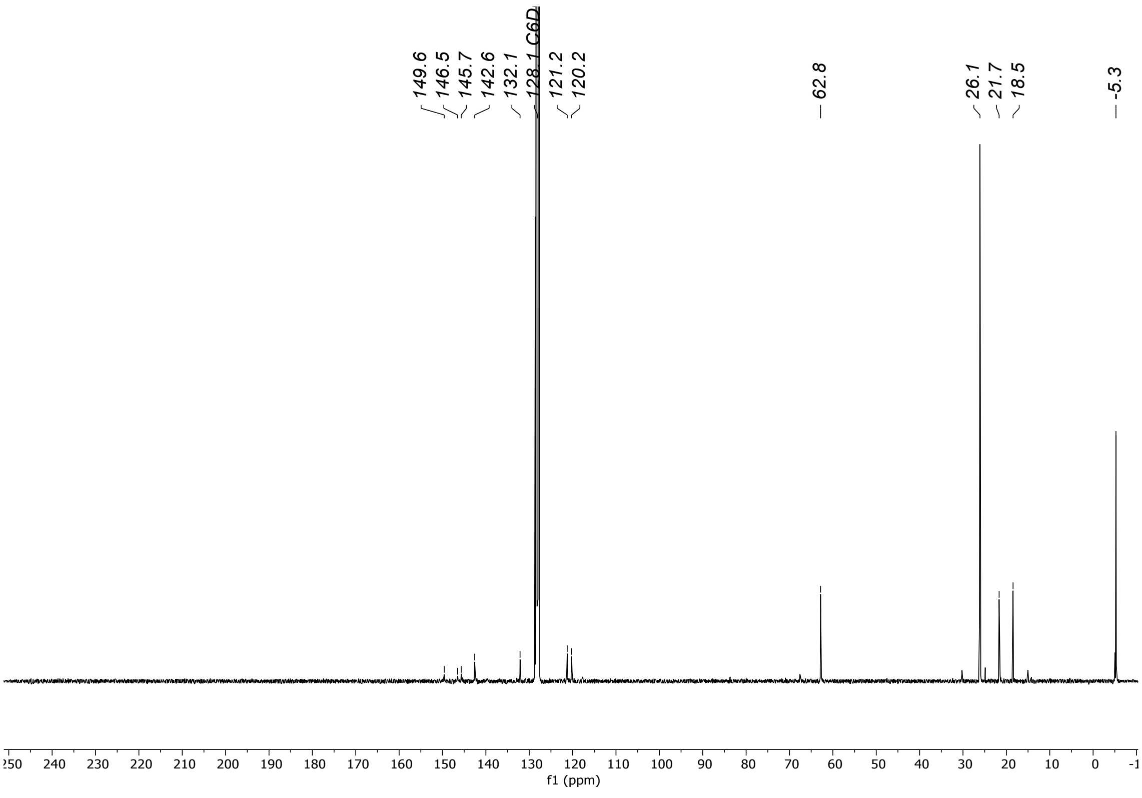

COSY (C_6D_6)

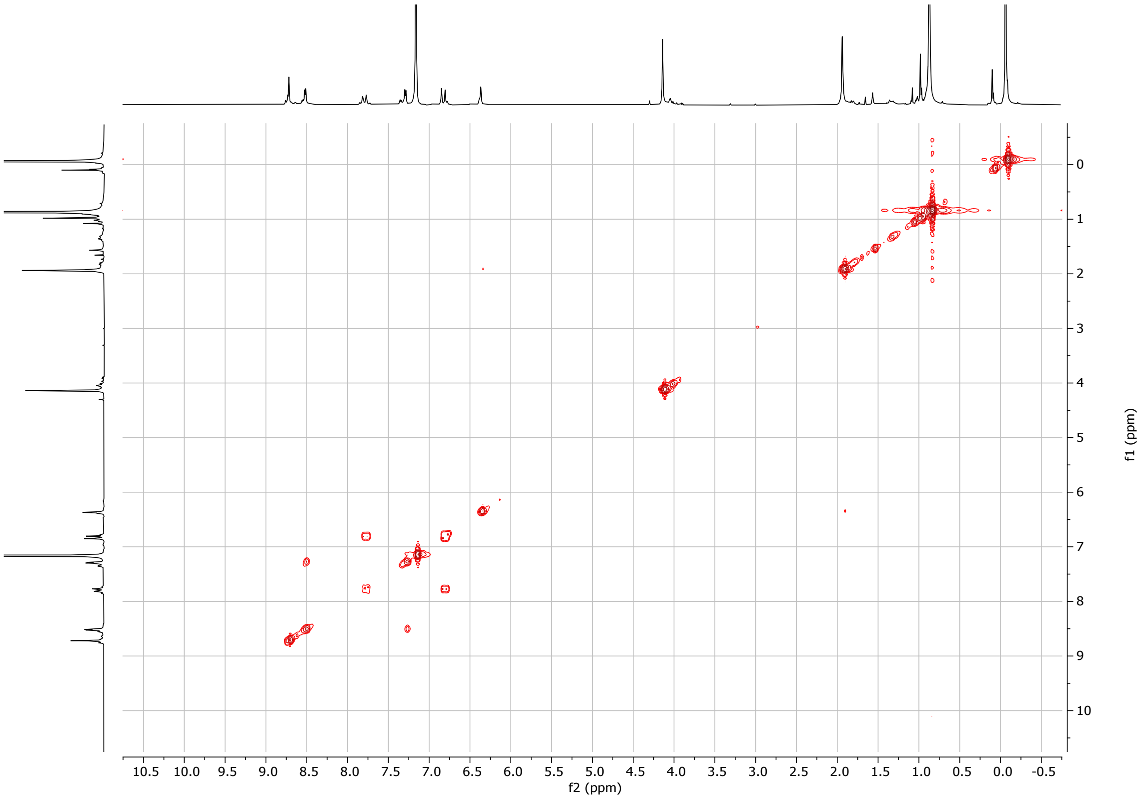

HSQC (C_6D_6)

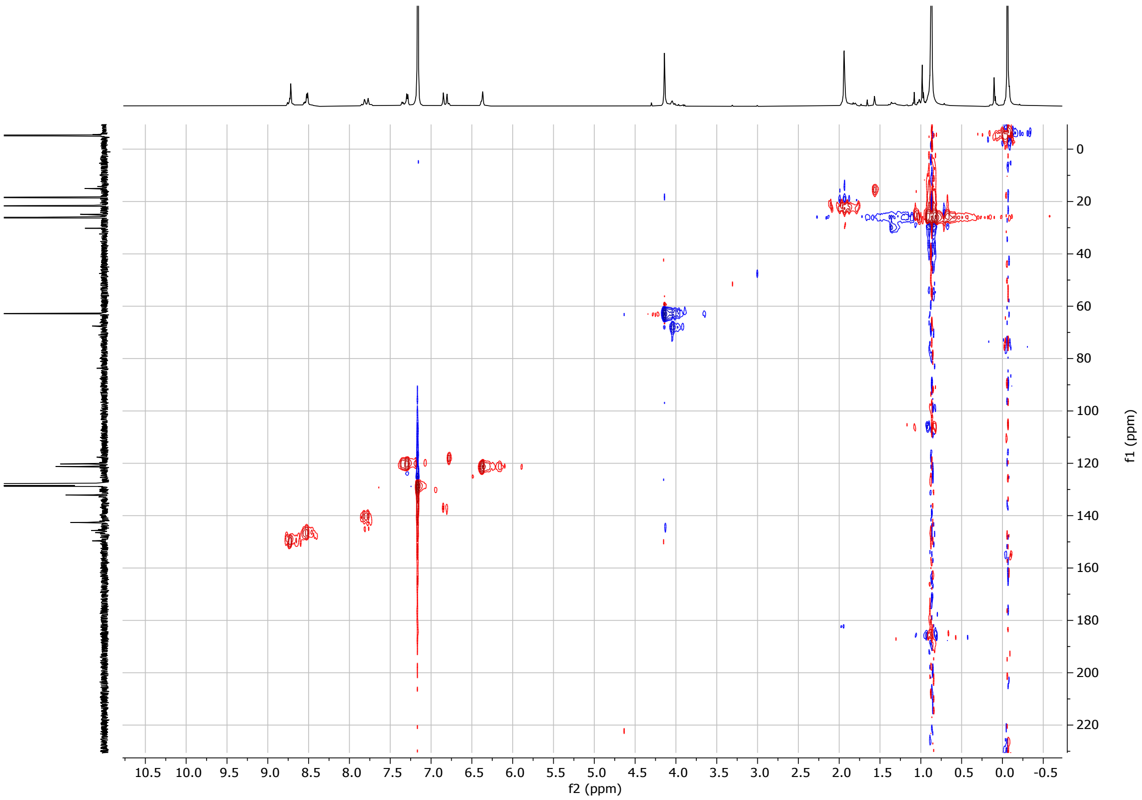

HMBC (C_6D_6)

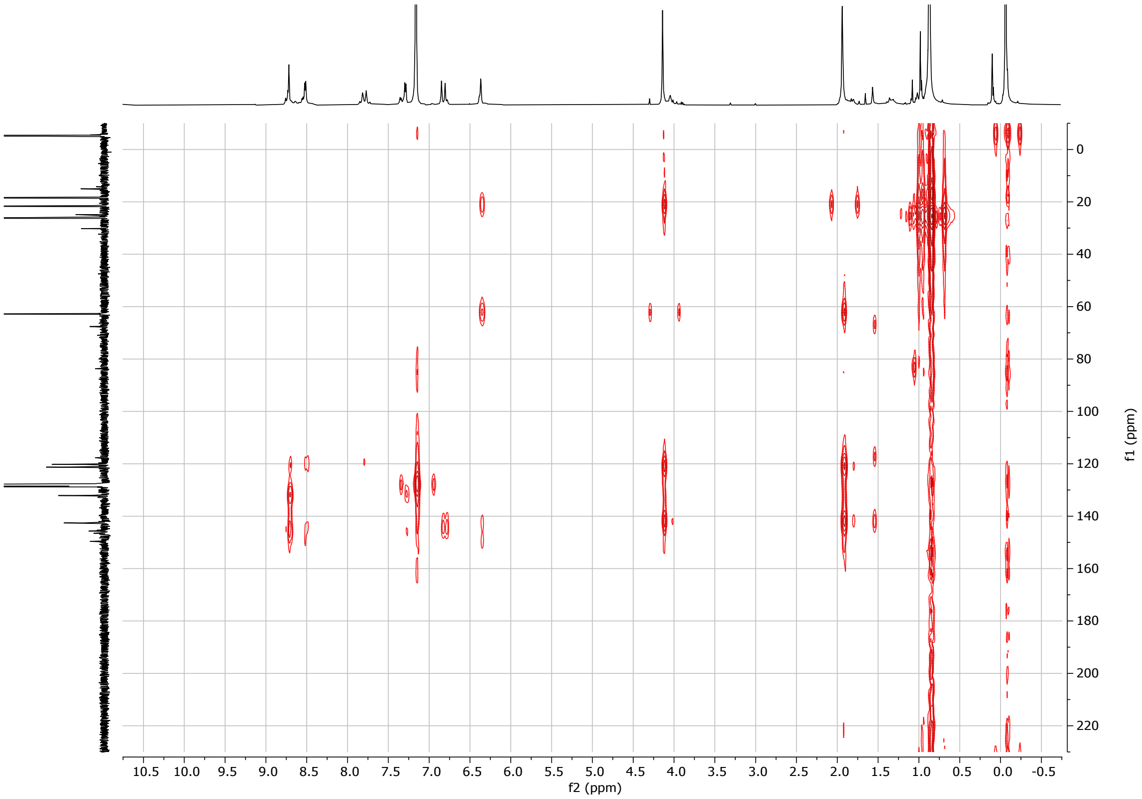

^{1}H-NMR (400.16 MHz, C_6D_6)

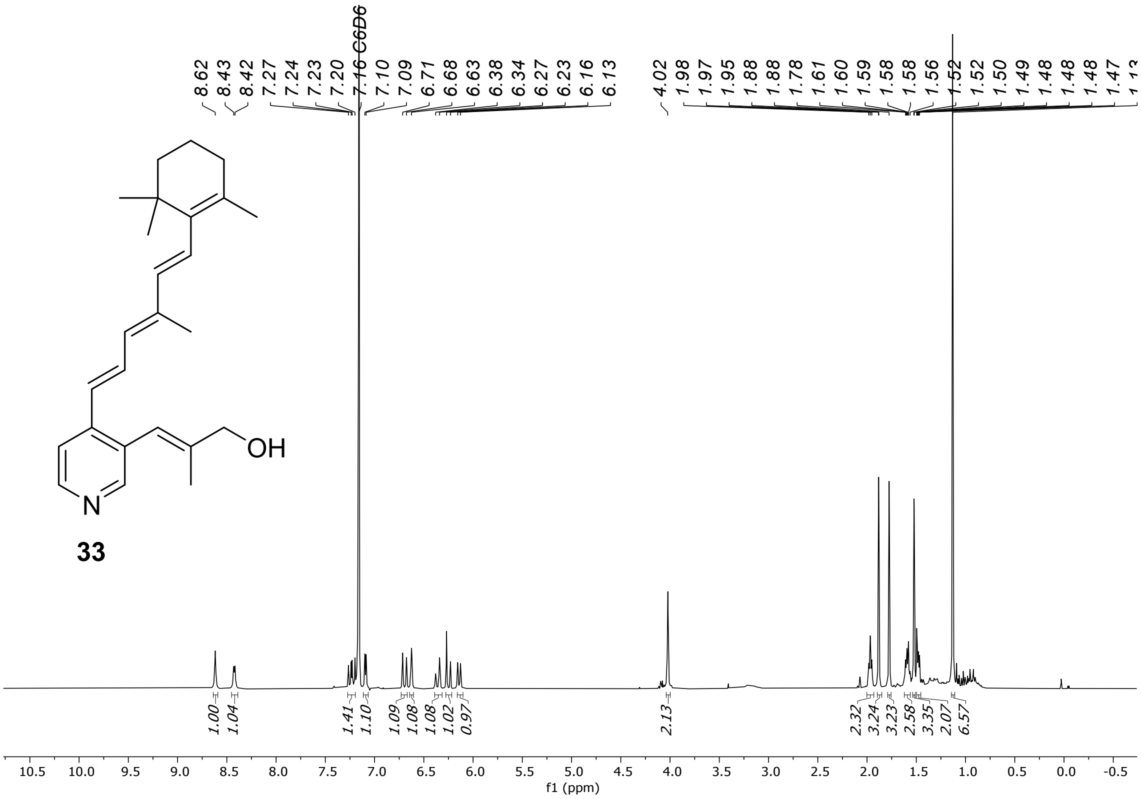

33

^{13}C-NMR (100.63 MHz, C_6D_6)

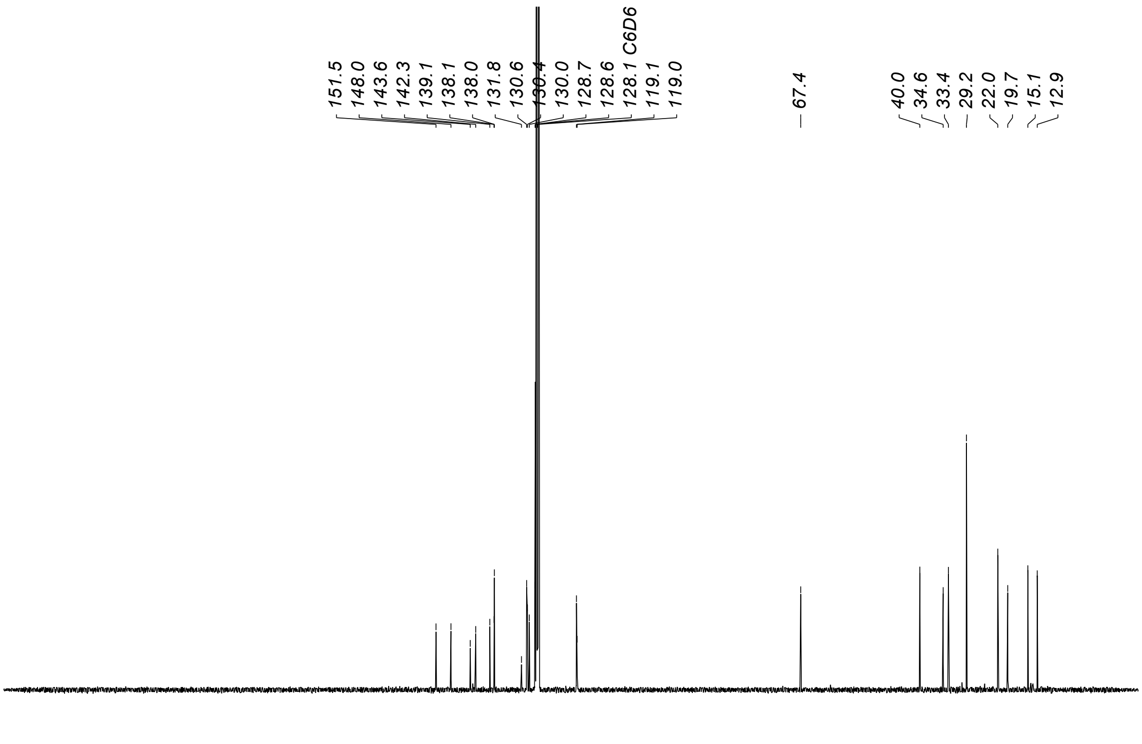

COSY (C_6D_6)

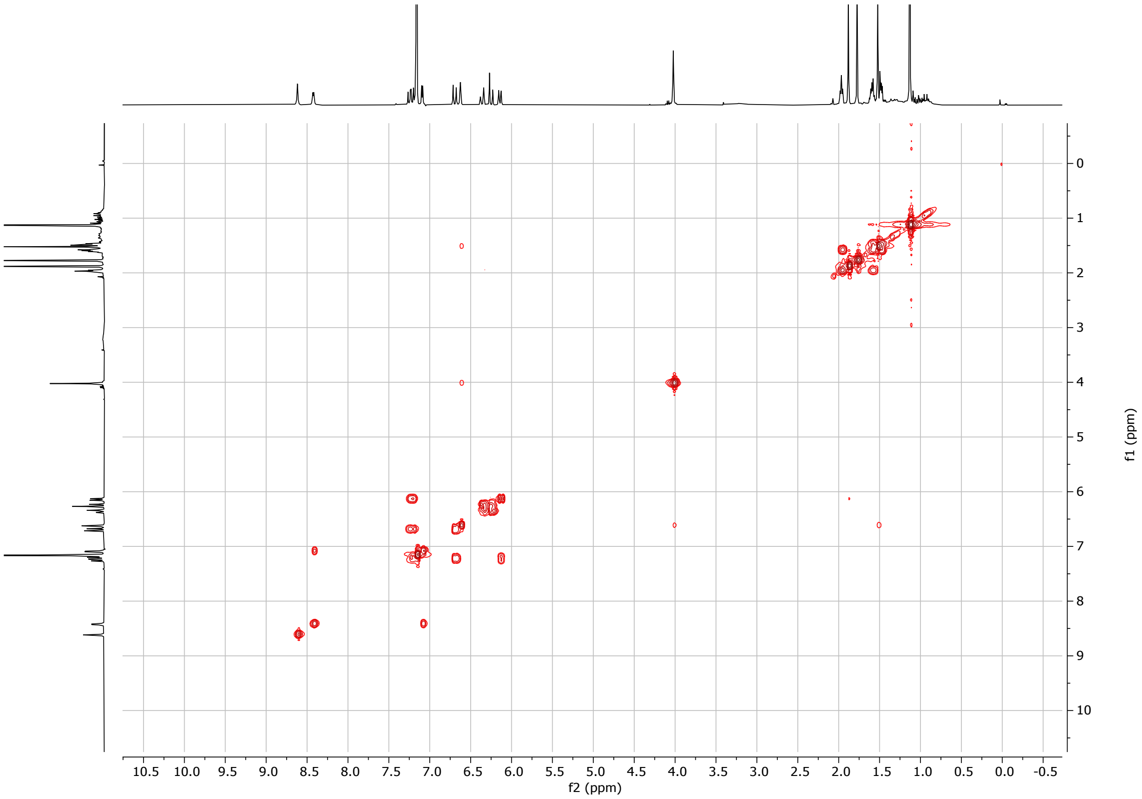

HSQC (C_6D_6)

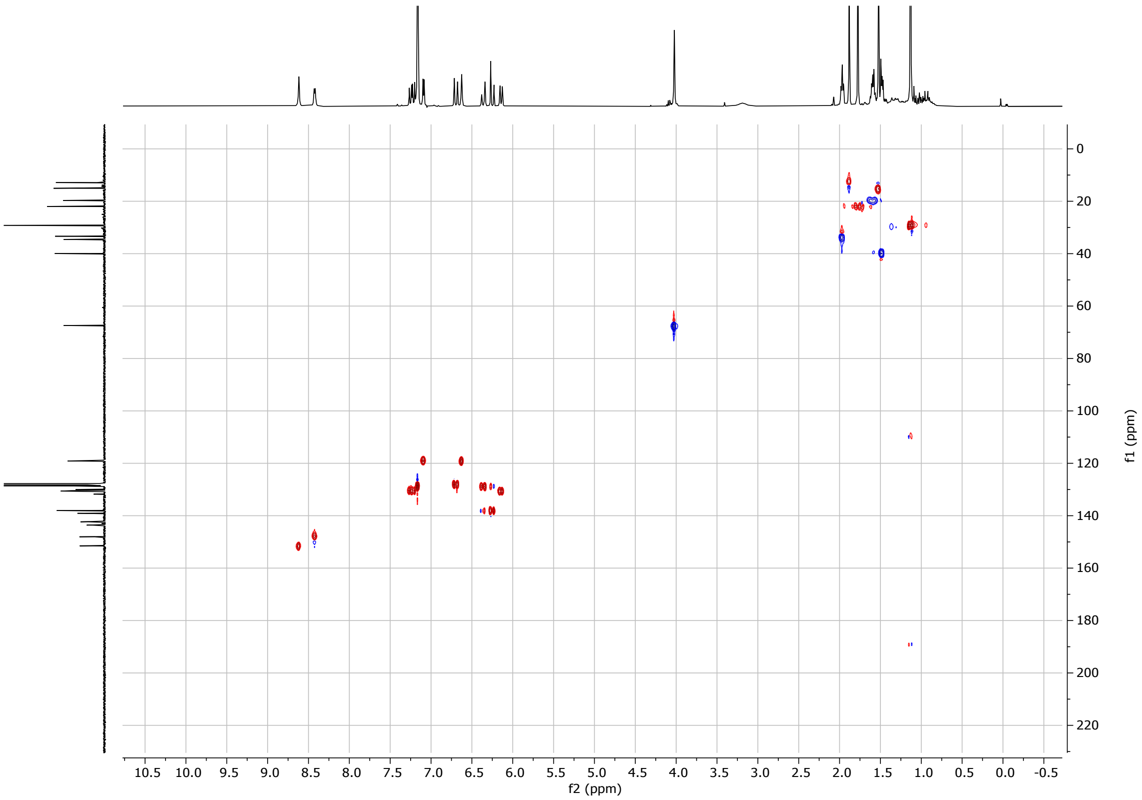

HMBC (C_6D_6)

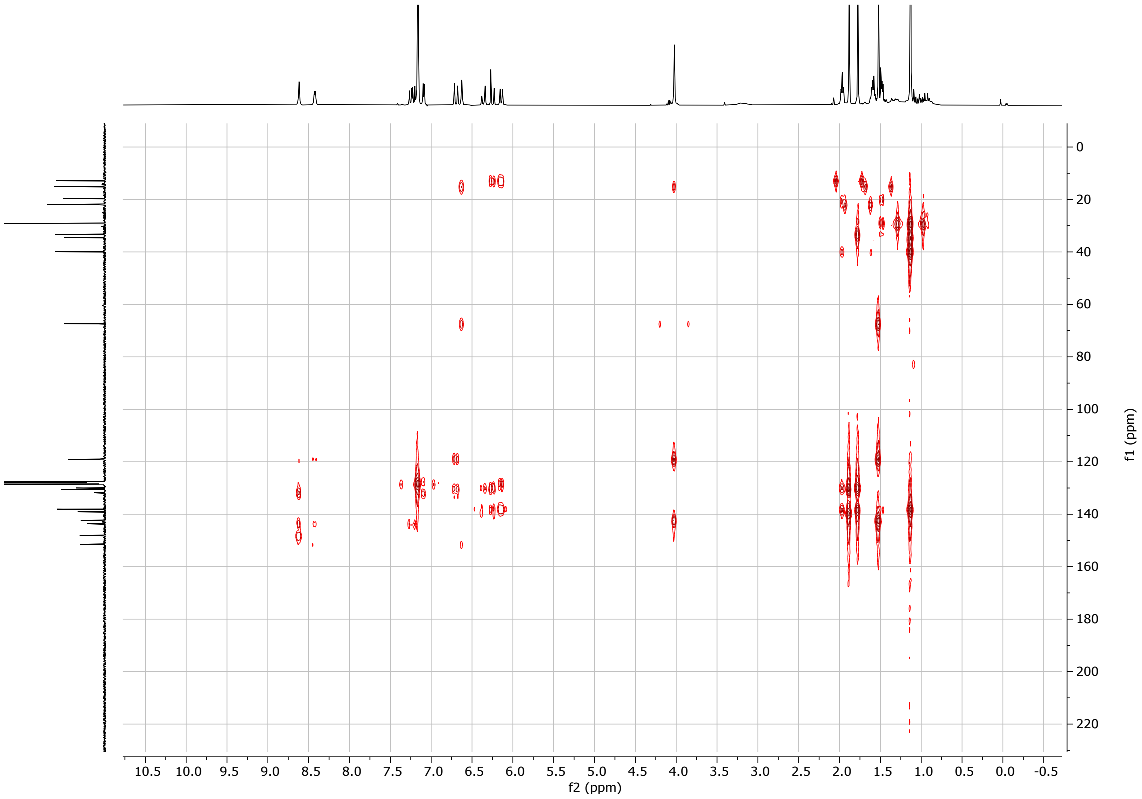

^{1}H-NMR (400.16 MHz, C_6D_6)

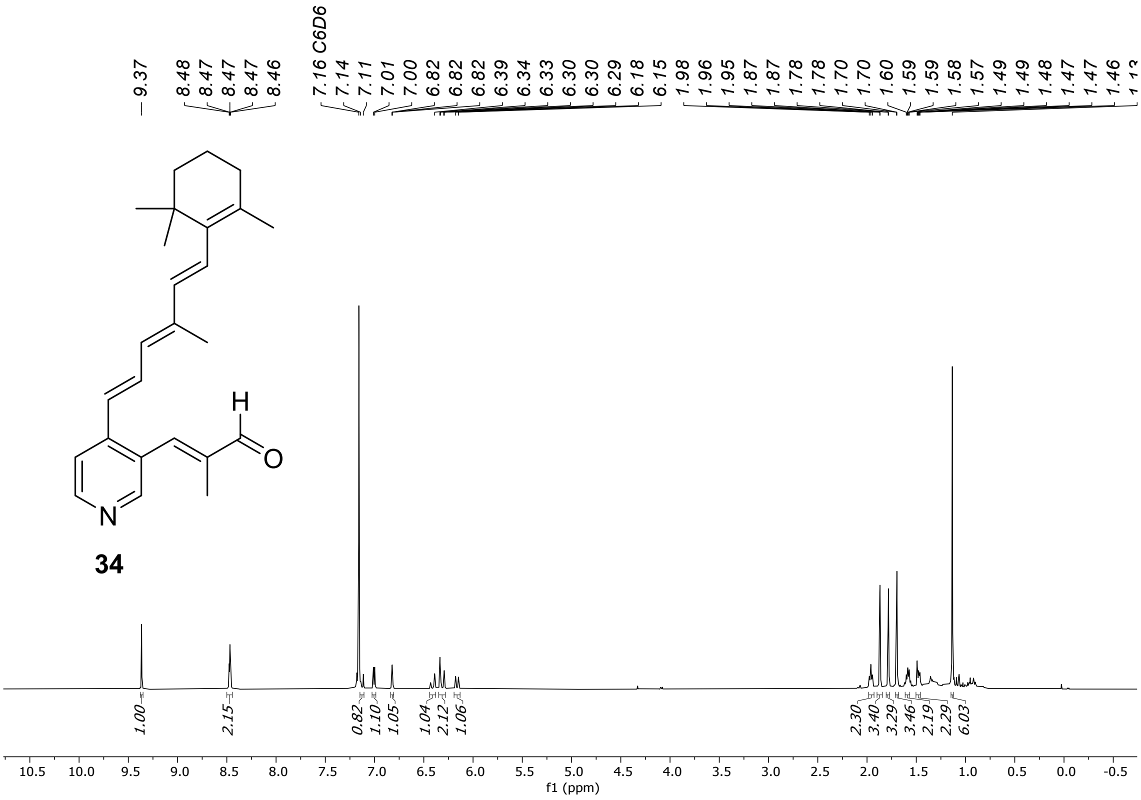

^{13}C-NMR (100.63 MHz, C_6D_6)

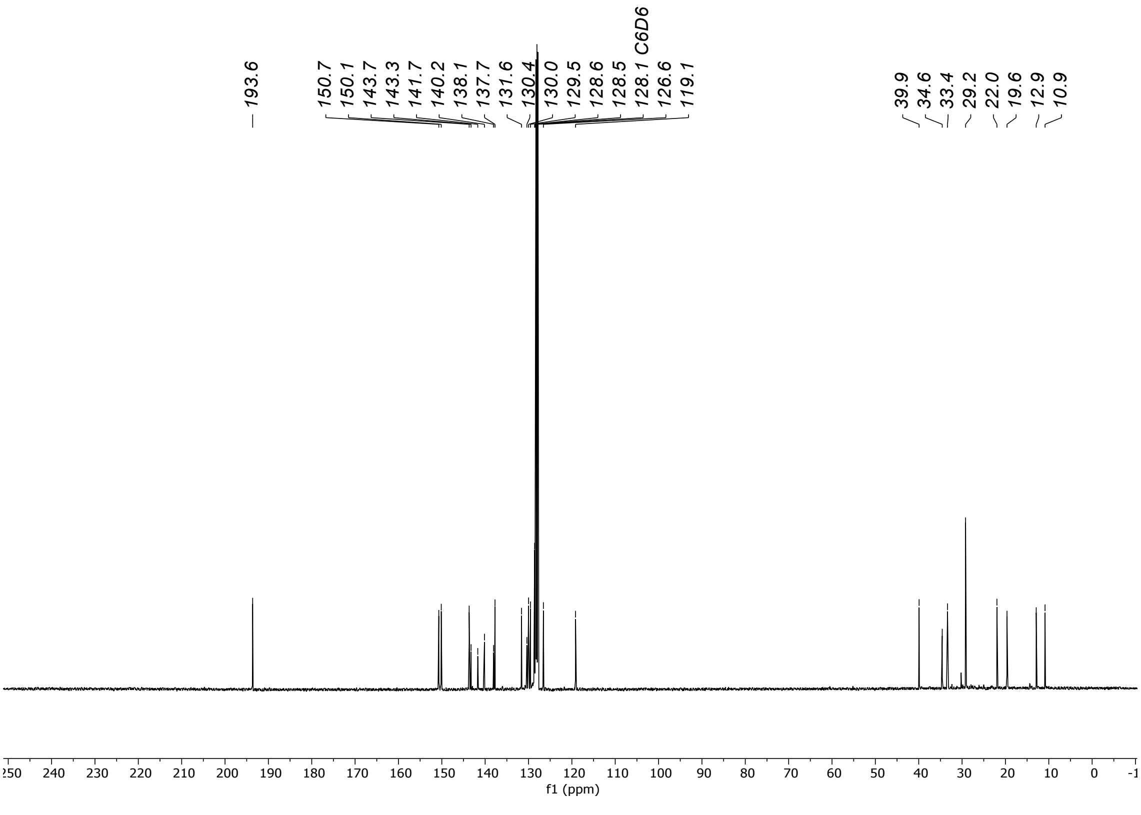

COSY (C_6D_6)

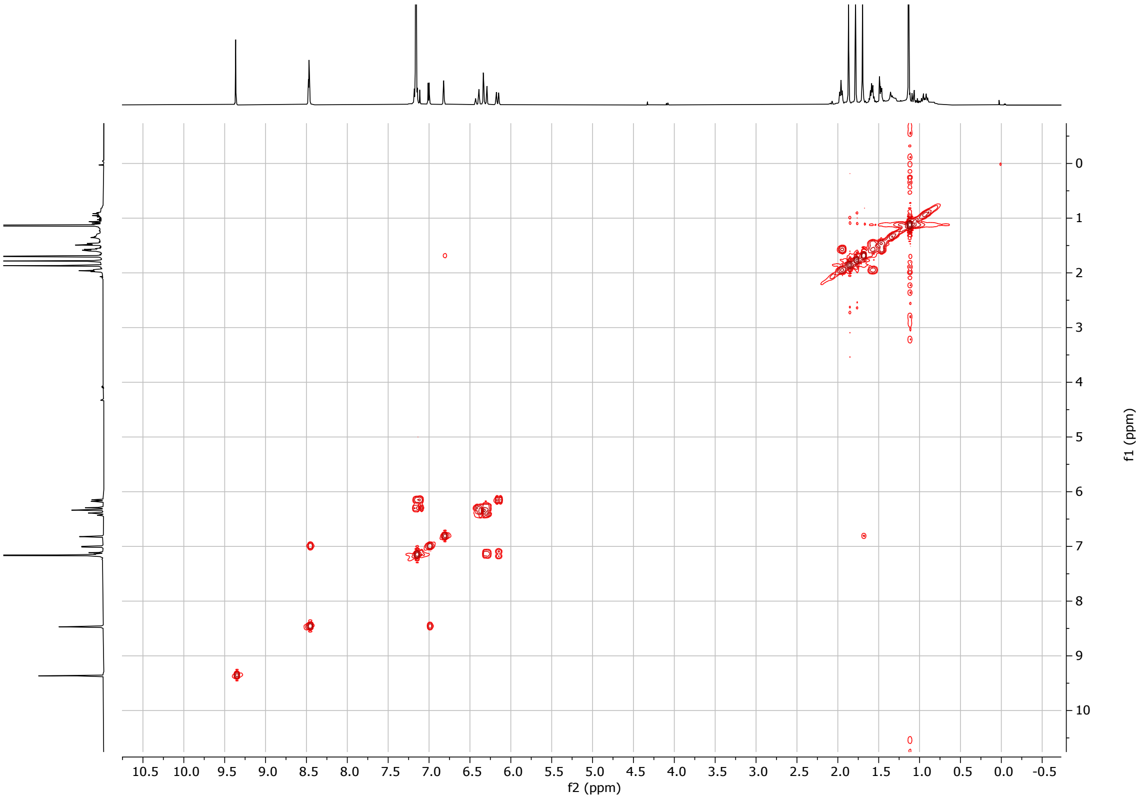

HSQC (C_6D_6)

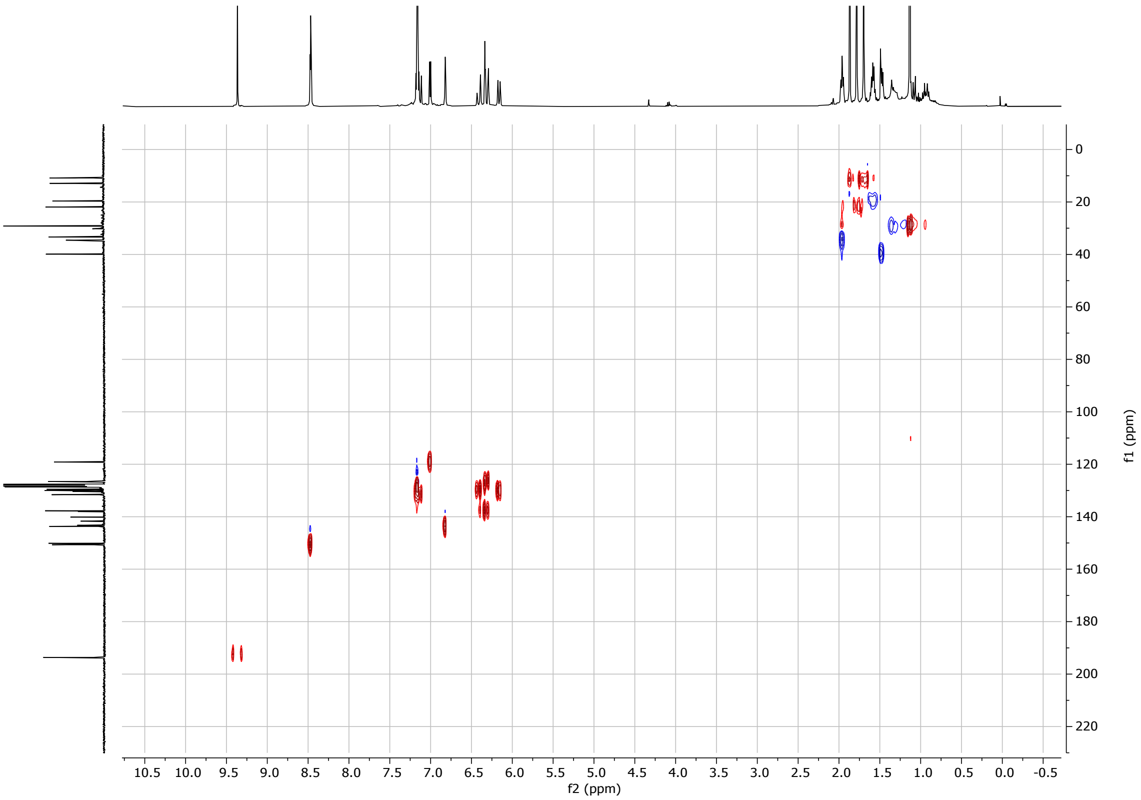

HMBC (C_6D_6)

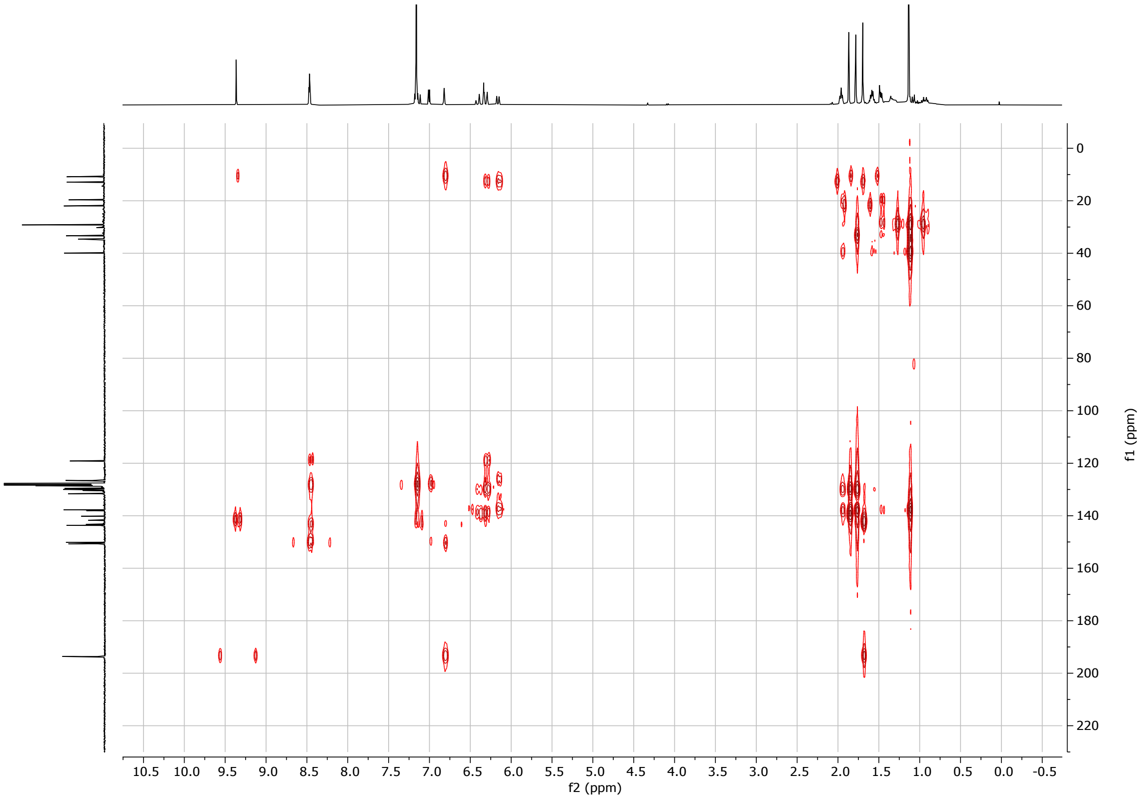

10.5 10.0 9.5 9.0 8.5 8.0 7.5 7.0 6.5 6.0 5.5 5.0 4.5 4.0 3.5 3.0 2.5 2.0 1.5 1.0 0.5 0.0 -0.5
f2 (ppm)
0 20 40 60 80 100 120 140 160 180 200 220
f1 (ppm)

^{1}H-NMR (400.16 MHz, C_6D_6)

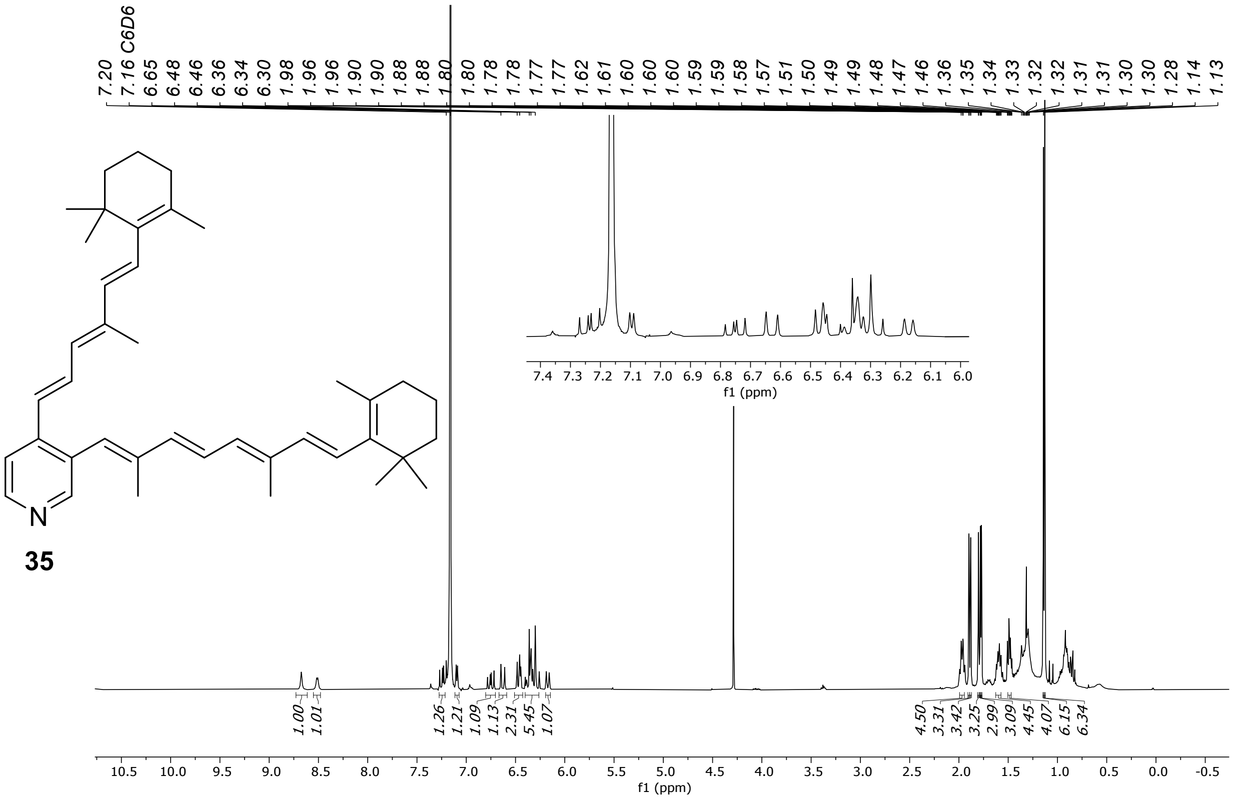

35

^{13}C-NMR (100.63 MHz, C_6D_6)

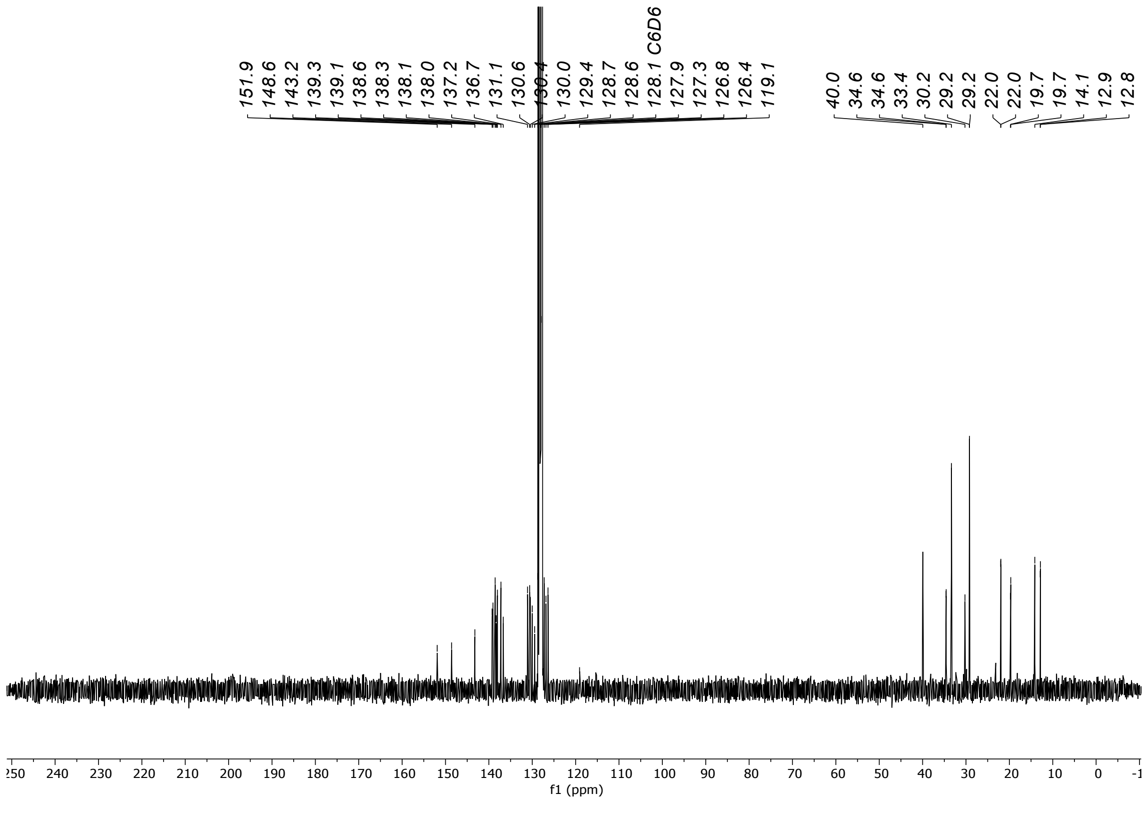

COSY (C_6D_6)

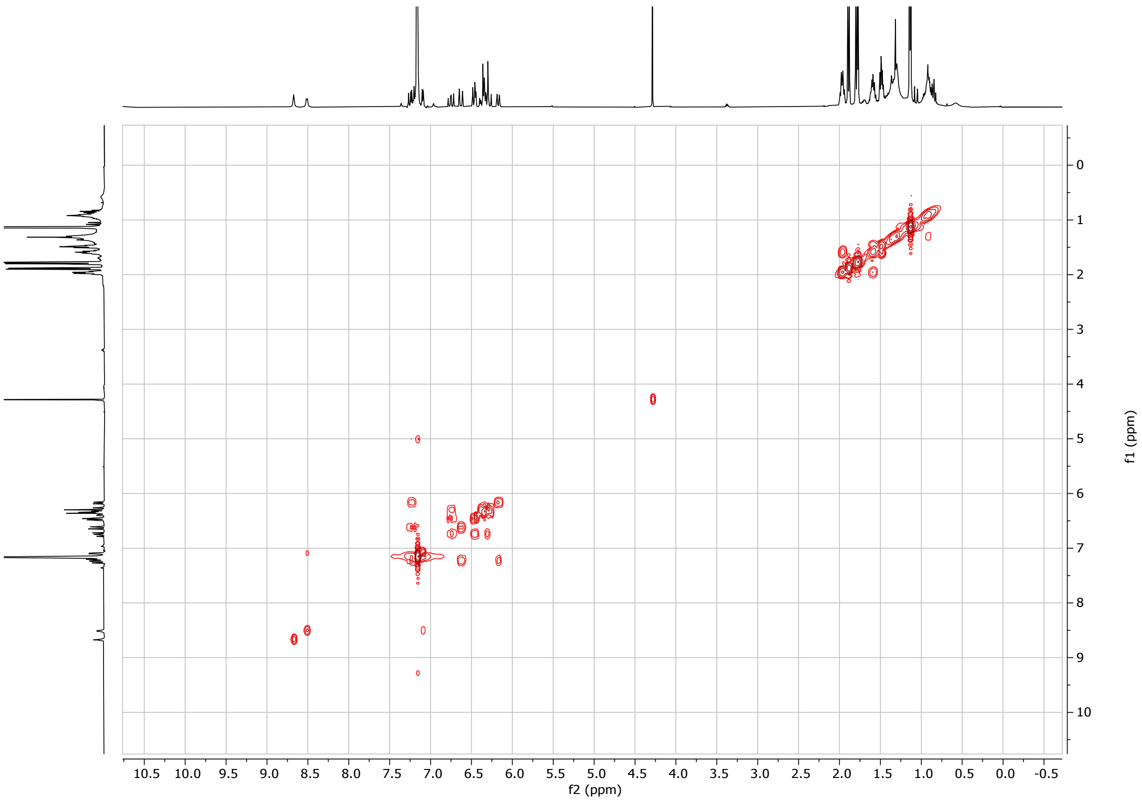

HSQC (C_6D_6)

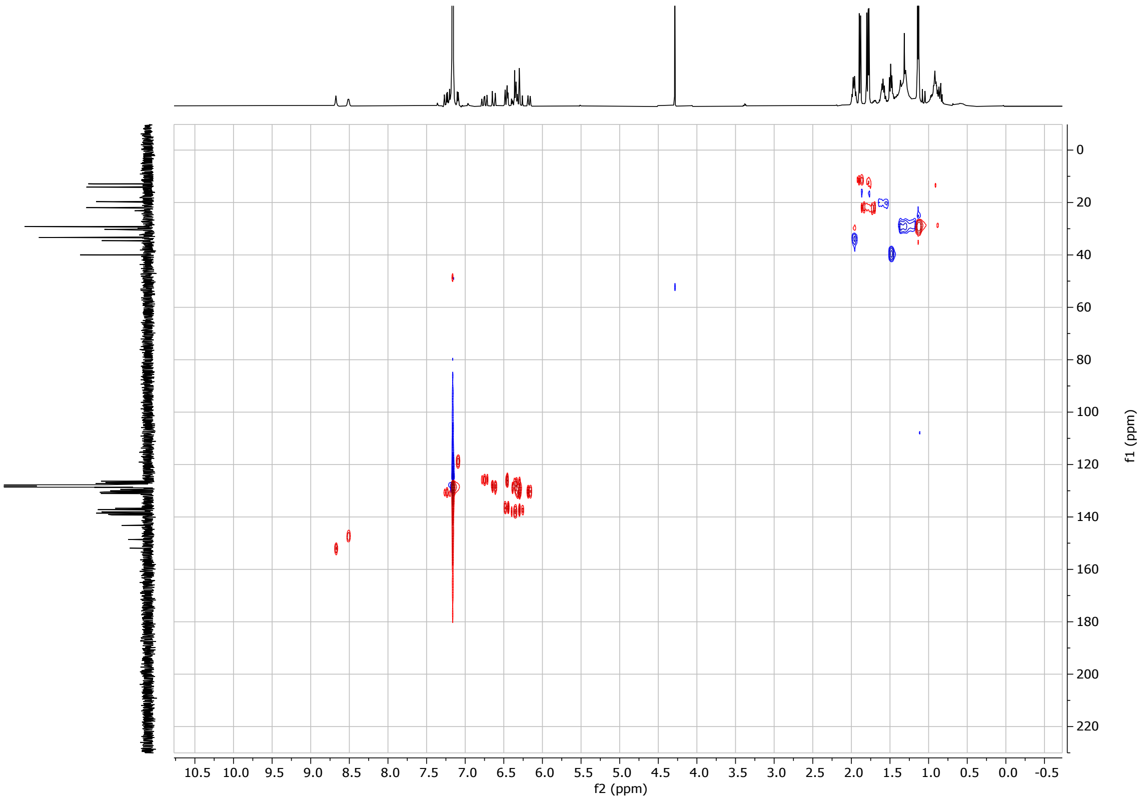

HMBC (C_6D_6)

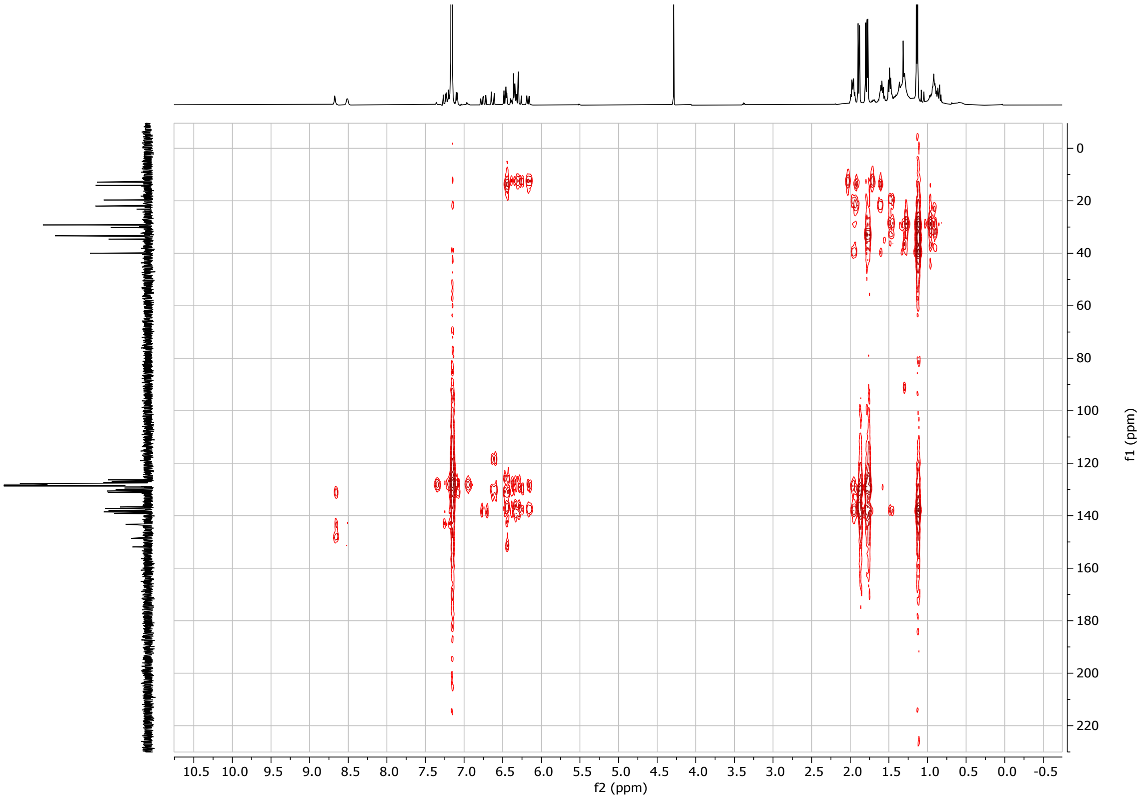

10.5 10.0 9.5 9.0 8.5 8.0 7.5 7.0 6.5 6.0 5.5 5.0 4.5 4.0 3.5 3.0 2.5 2.0 1.5 1.0 0.5 0.0 -0.5
f2 (ppm)
0 20 40 60 80 100 120 140 160 180 200 220
f1 (ppm)

^{1}H-NMR (400.16 MHz, CD_3OD)

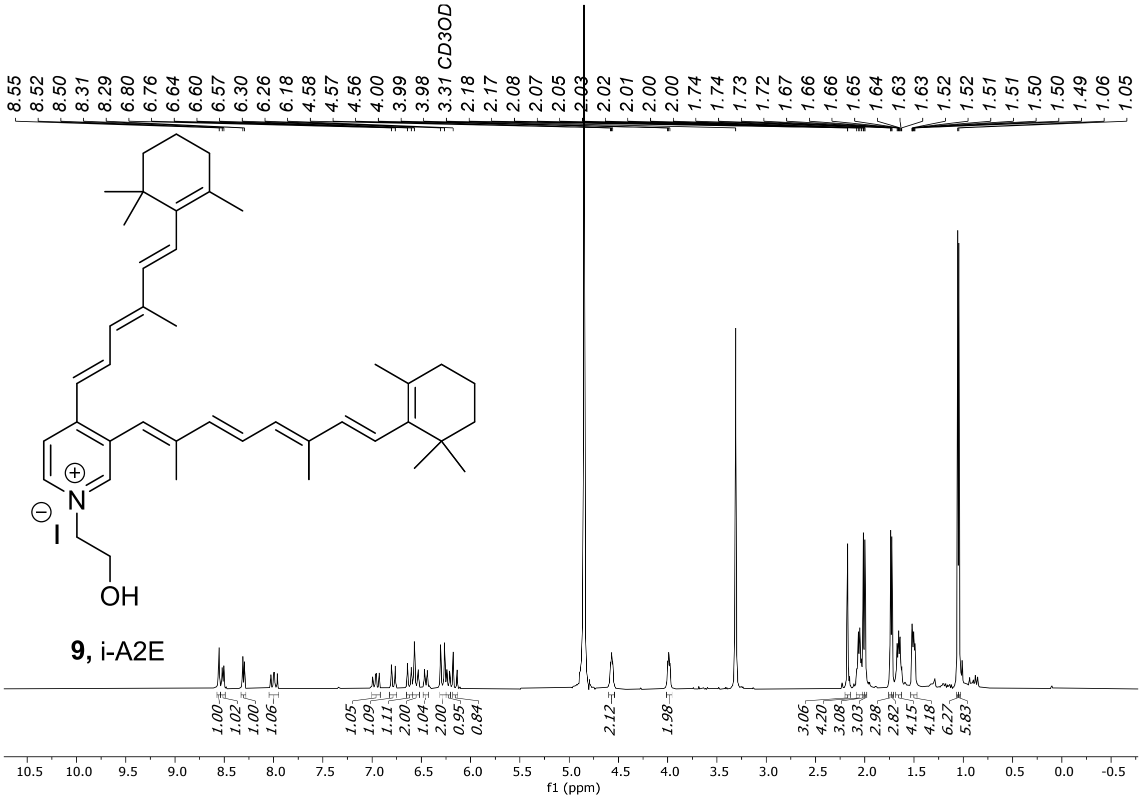

^{13}C-NMR (100.63 MHz, CD_3OD)

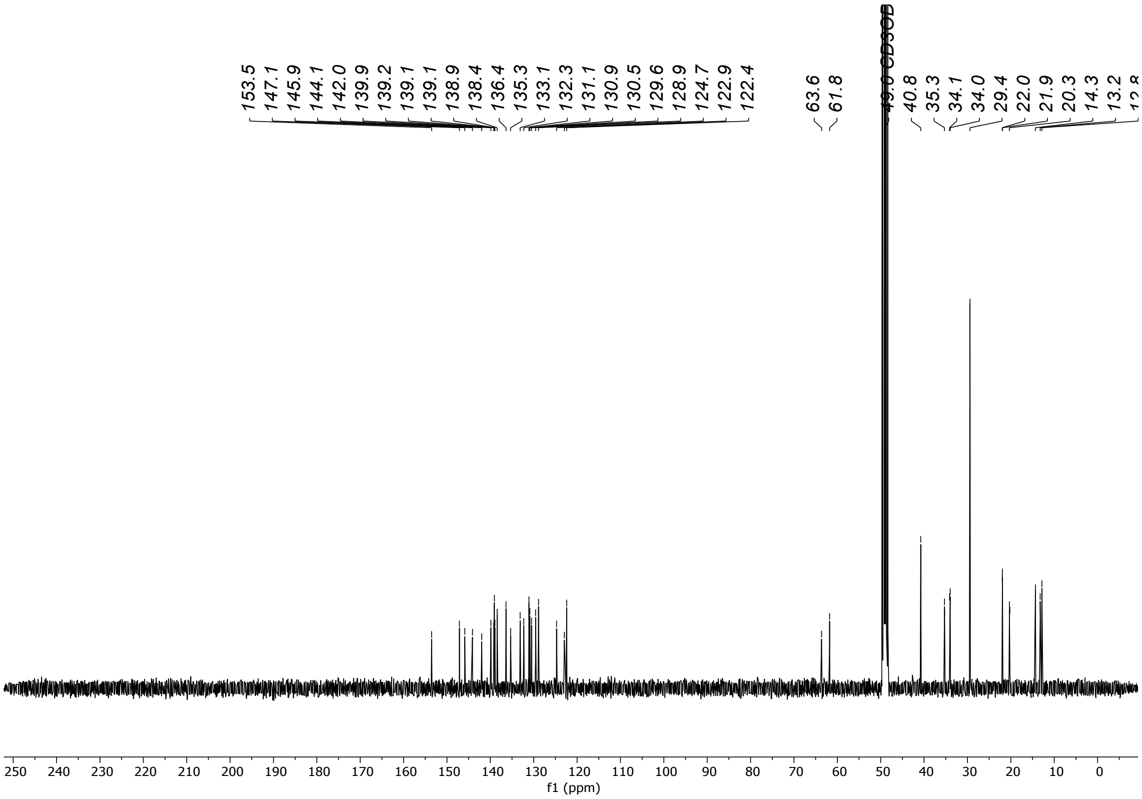

COSY (CD_3OD)

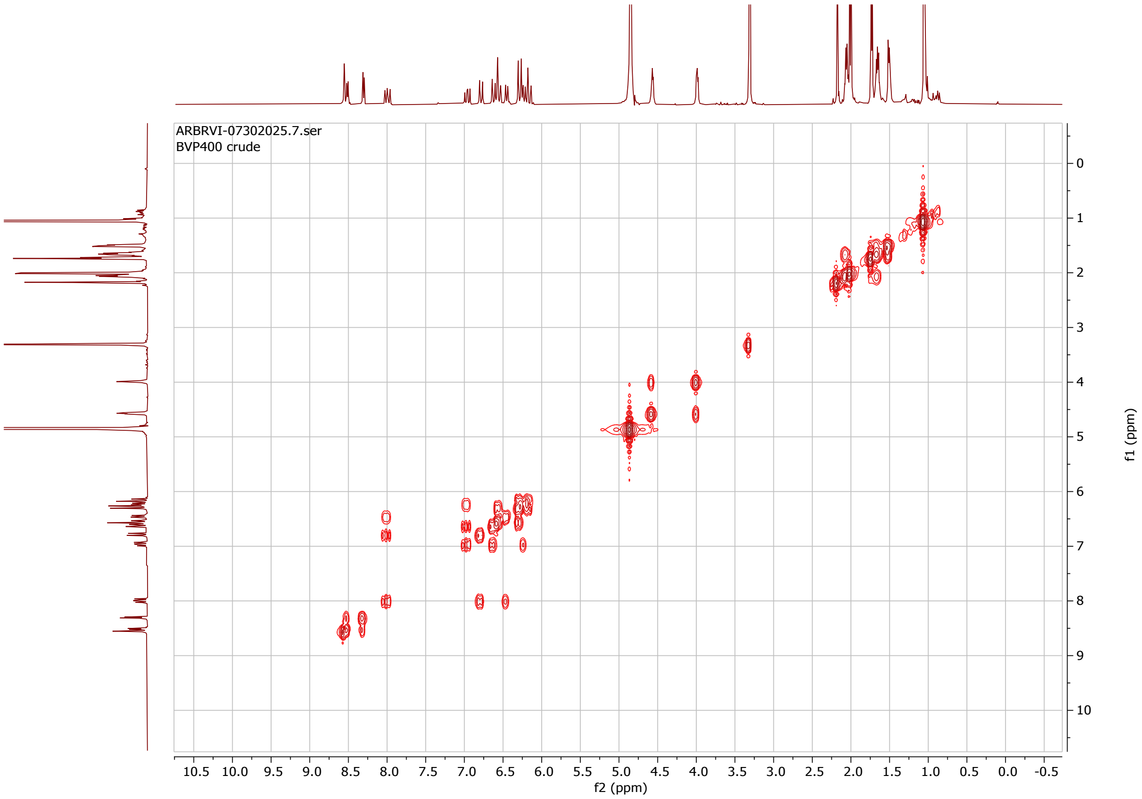

HSQC (CD_3OD)

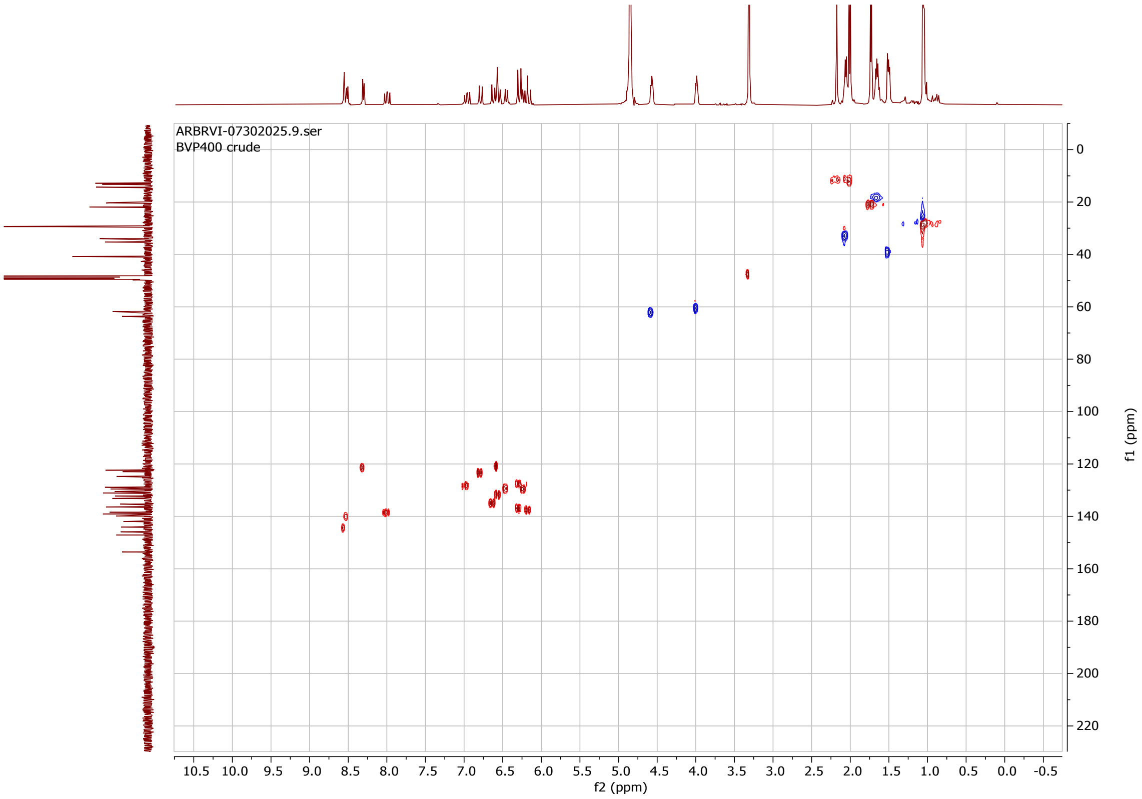

HMBC (CD_3OD)

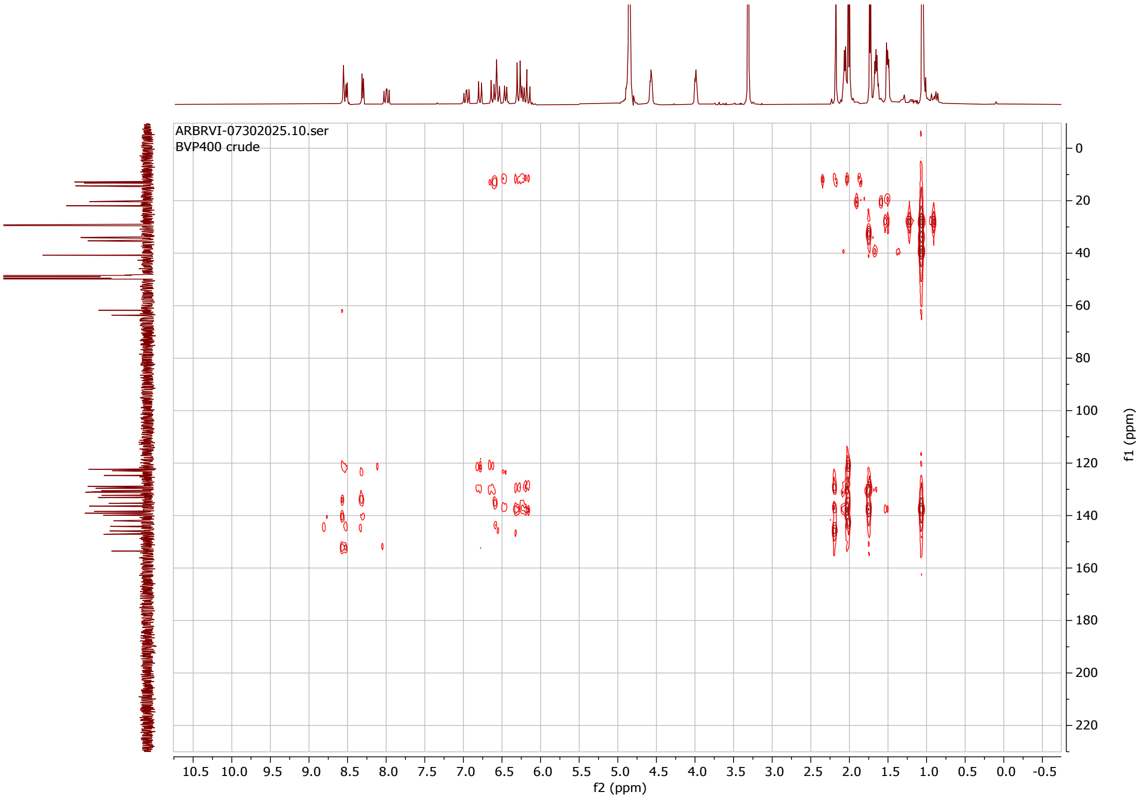

NOE-1D (400.16 MHz, freq. 7.99 ppm, CD_3OD)

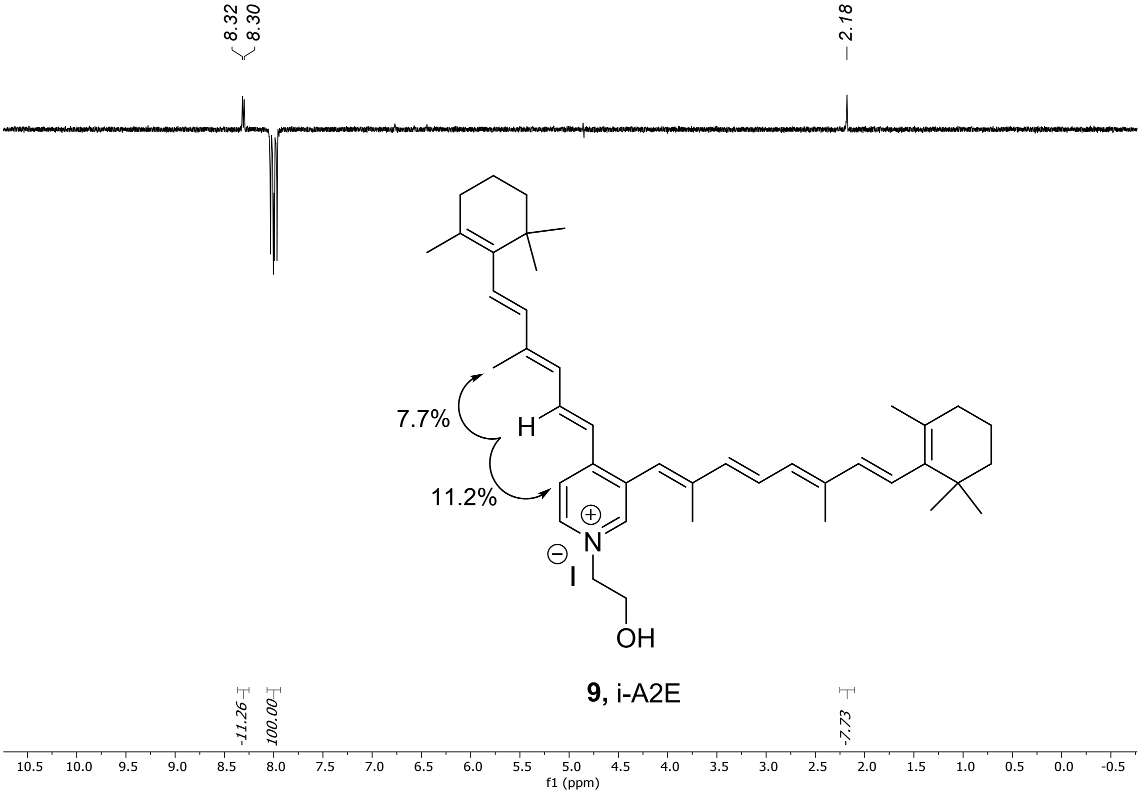

9, i-A2E

NOE-1D (400.16 MHz, freq. 6.96 ppm, CD_3OD)

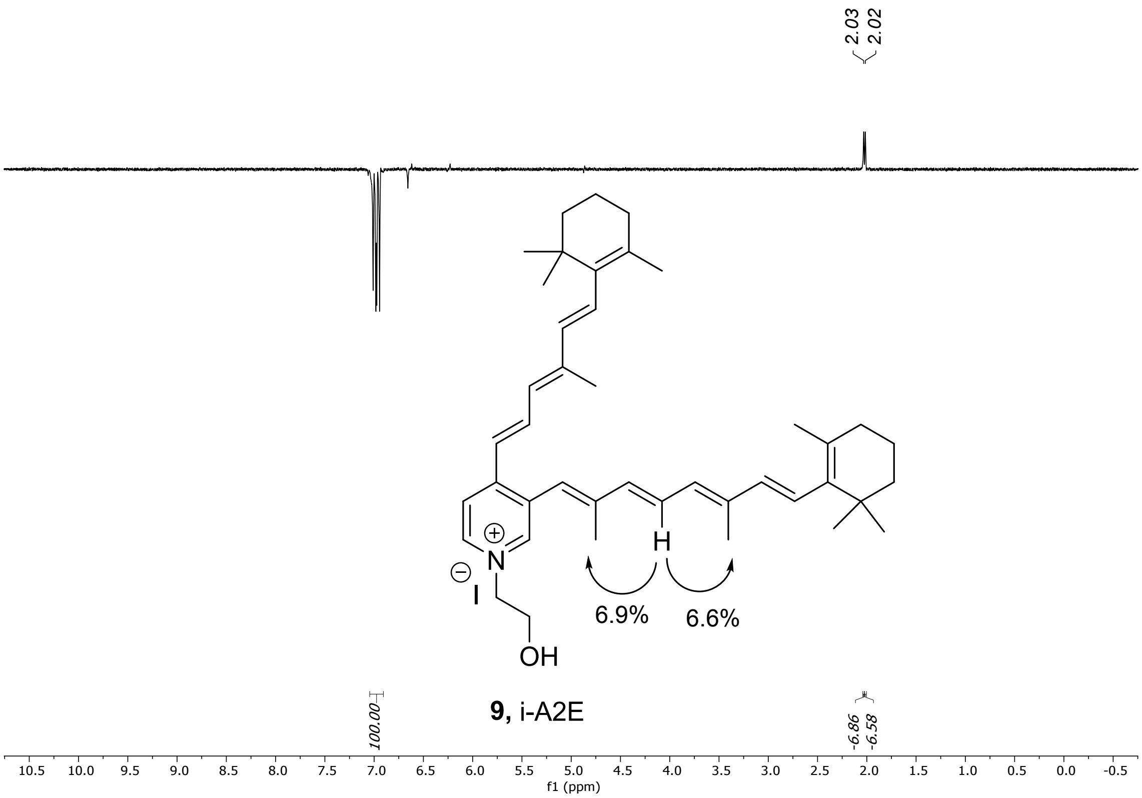

NOE-1D (400.16 MHz, freq. 2.18 ppm, CD_3OD)

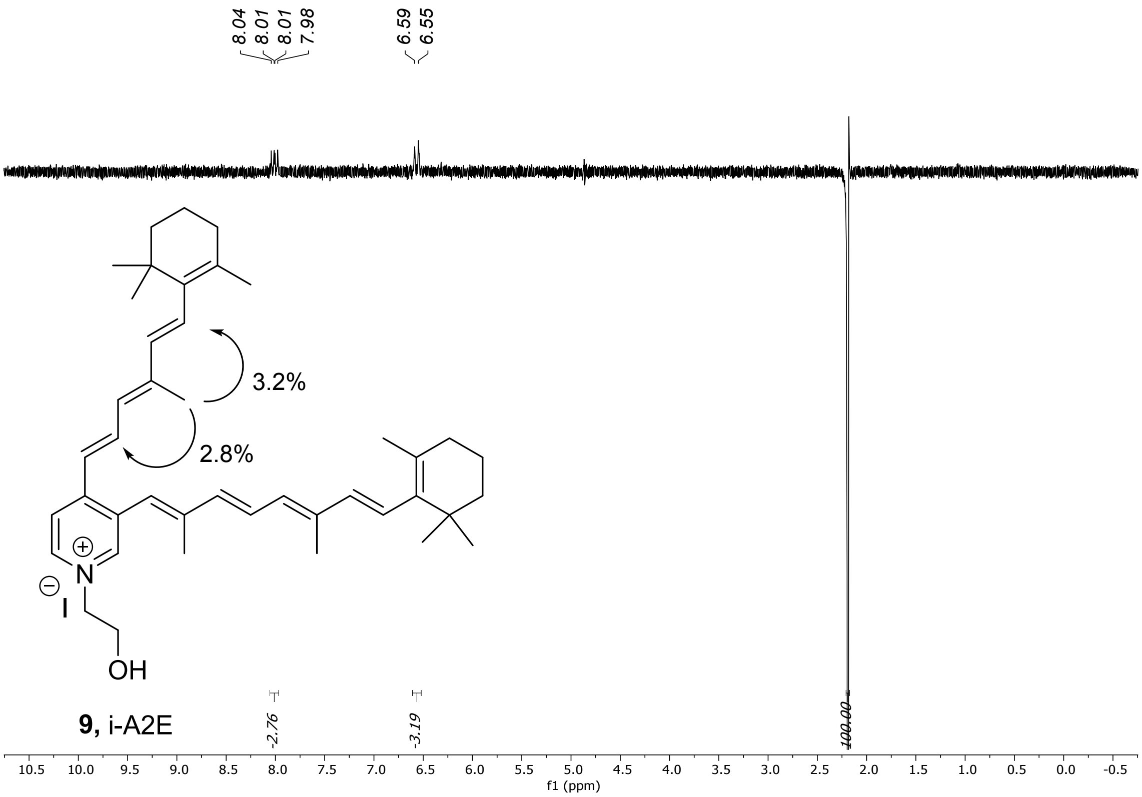

Supplement: Supplementary file 1 [file ol5c04694_si_001.pdf]
